# Endoplasmic reticulum-plasma membrane contact sites integrate sterol and phospholipid regulation

**DOI:** 10.1371/journal.pbio.2003864

**Published:** 2018-05-21

**Authors:** Evan Quon, Yves Y. Sere, Neha Chauhan, Jesper Johansen, David P. Sullivan, Jeremy S. Dittman, William J. Rice, Robin B. Chan, Gilbert Di Paolo, Christopher T. Beh, Anant K. Menon

**Affiliations:** 1 Department of Molecular Biology and Biochemistry, Simon Fraser University, Burnaby, British Columbia, Canada; 2 Department of Biochemistry, Weill Cornell Medical College, New York, New York, United States of America; 3 Simons Electron Microscopy Center at the New York Structural Biology Center, New York, New York, United States of America; 4 Department of Pathology and Cell Biology, Columbia University College of Physicians and Surgeons, New York, New York, United States of America; 5 Denali Therapeutics, South San Francisco, California, United States of America; 6 Centre for Cell Biology, Development, and Disease, Simon Fraser University, Burnaby, British Columbia, Canada; UT Southwestern Medical Center, United States of America

## Abstract

Tether proteins attach the endoplasmic reticulum (ER) to other cellular membranes, thereby creating contact sites that are proposed to form platforms for regulating lipid homeostasis and facilitating non-vesicular lipid exchange. Sterols are synthesized in the ER and transported by non-vesicular mechanisms to the plasma membrane (PM), where they represent almost half of all PM lipids and contribute critically to the barrier function of the PM. To determine whether contact sites are important for both sterol exchange between the ER and PM and intermembrane regulation of lipid metabolism, we generated Δ-super-tether (Δ-s-tether) yeast cells that lack six previously identified tethering proteins (yeast extended synatotagmin [E-Syt], vesicle-associated membrane protein [VAMP]-associated protein [VAP], and TMEM16-anoctamin homologues) as well as the presumptive tether Ice2. Despite the lack of ER-PM contacts in these cells, ER-PM sterol exchange is robust, indicating that the sterol transport machinery is either absent from or not uniquely located at contact sites. Unexpectedly, we found that the transport of exogenously supplied sterol to the ER occurs more slowly in Δ-s-tether cells than in wild-type (WT) cells. We pinpointed this defect to changes in sterol organization and transbilayer movement within the PM bilayer caused by phospholipid dysregulation, evinced by changes in the abundance and organization of PM lipids. Indeed, deletion of either *OSH4*, which encodes a sterol/phosphatidylinositol-4-phosphate (PI4P) exchange protein, or *SAC1*, which encodes a PI4P phosphatase, caused synthetic lethality in Δ-s-tether cells due to disruptions in redundant PI4P and phospholipid regulatory pathways. The growth defect of Δ-s-tether cells was rescued with an artificial "ER-PM staple," a tether assembled from unrelated non-yeast protein domains, indicating that endogenous tether proteins have nonspecific bridging functions. Finally, we discovered that sterols play a role in regulating ER-PM contact site formation. In sterol-depleted cells, levels of the yeast E-Syt tether Tcb3 were induced and ER-PM contact increased dramatically. These results support a model in which ER-PM contact sites provide a nexus for coordinating the complex interrelationship between sterols, sphingolipids, and phospholipids that maintain PM composition and integrity.

## Author summary

Almost half of the inner surface area of the yeast plasma membrane (PM) is covered with closely associated cortical endoplasmic reticulum (ER). In yeast and human cells, it has been proposed that ER-anchored tether proteins staple the ER to the PM, creating membrane contact sites at which lipid transport between the ER and PM and membrane lipid synthesis are coordinately regulated, but the potential mechanisms are unclear. Here, we test this idea by creating yeast cells that lack all ER-PM tethers. We find that whereas the bidirectional transport of sterols between the ER and PM is unaffected in these cells, sterols within the PM are disorganized due to disruptions in phospholipid biosynthesis that alter PM lipid composition. In particular, we show that phosphatidylinositol-4-phosphate, a phospholipid needed for intracellular signaling and membrane trafficking, accumulates within the PM. Some of these defects can be rescued by reinstating membrane contacts via expression of an artificial tether. However, correction is also achieved without the creation of contacts by supplementing the growth medium with a precursor of membrane phospholipids. Based on these results, we propose that ER-PM contacts do not play a major role as physical conduits for lipid exchange but rather serve as regulatory interfaces to integrate lipid synthesis pathways.

## Introduction

Most lipids are synthesized in the endoplasmic reticulum (ER) and distributed to other membranes by non-vesicular mechanisms. These mechanisms act in conjunction with lipid metabolic networks to maintain the unique lipid profile of the plasma membrane (PM) and subcellular organelles, and enable rapid membrane lipid remodeling in response to signals and stresses [1–3]. An attractive hypothesis is that non-vesicular lipid transport and lipid biosynthetic and regulatory pathways intersect at ER-PM membrane contact sites (MCSs), where protein tethers retain the ER and PM within about 15–60 nm of each other [4–9]. In this view, ER-PM MCSs would serve as a nexus, coordinating requirements in the PM for lipids with their production in the ER [3, 9]. How this coordination is accomplished is not well understood. Here, we report on the interplay between sterol and phospholipid homeostasis at ER-PM MCSs.

Cholesterol—and its yeast counterpart ergosterol—are synthesized in the ER and transported by non-vesicular mechanisms to the PM [10, 11], where they are found at high concentrations corresponding to about 40 mole percent of PM lipids, i.e., one out of every two to three lipids in the PM is a sterol. The spontaneous exchange of sterols between membranes is slow in vitro and undetectable in vivo, primarily because sterol desorption from the membrane is energetically expensive [12–14]. To move sterols efficiently between the ER, PM, and other membranes, cells make use of sterol transport proteins (STPs), whose proposed function is mainly to reduce the energy barrier for sterol desorption, thereby extracting sterols into a binding pocket within the protein for transit through the cytoplasm [12]. STPs may operate freely in the cytoplasm or at MCSs. Soluble and membrane-bound STPs might work in parallel to provide redundant mechanisms for sterol exchange. As transport is predicted to be rate-limited by the desorption step rather than diffusion of the STP–sterol complex through the cytoplasm [12], the proximity of the ER to the PM at an MCS may not determine the sterol transport rate unless STPs are restricted to these sites. Because a number of sterol biosynthetic enzymes are enriched in PM-associated ER membrane fractions [4], it is attractive to consider that the biosynthetic and transport machineries may colocalize to ER-PM MCSs, effectively channeling sterol between compartments [9] to facilitate sterol homeostasis.

The identity of STPs is controversial and the role of MCSs in sterol transport is unexplored. STP candidates in yeast include members of two protein families: soluble Osh proteins (related to mammalian oxysterol-binding protein [OSBP] [15]) and membrane-bound lipid transfer proteins anchored at MCSs (Lam) (members of the StARkin superfamily of steroidogenic acute regulatory [StAR] protein–related lipid transfer [StART] proteins [16, 17]). Osh4 binds sterols and phosphatidylinositol-4-phosphate (PI4P) [18] and by toggling between its sterol and PI4P bound states, it has been shown to transport sterol against a concentration gradient between vesicle populations in vitro [19]. While this activity may account for certain aspects of sterol homeostasis, Osh4 is not required for retrograde sterol transport [20], nor is it essential for the high rate of sterol exchange between the ER and PM, as evinced by robust sterol transport in cells where all seven *OSH* genes are inactivated (*osh*Δ) [21]. The seven Osh proteins share overlapping essential activities [22], but because Osh6 and several other Osh proteins do not bind sterols [23–25], sterol transport is not a function shared by the entire family. Lam proteins each have one or two sterol-binding StARkin domains. The purified domains have been shown to catalyze sterol exchange between vesicles in vitro [26–28]. Lam1–Lam4 are integral ER membrane proteins located at the cell cortex, where they might function as sterol transporters, similar to the mammalian StARkin STARD3, which is anchored to endosomal membranes and has been suggested to facilitate endosome–ER cholesterol transfer [29]. Although elimination of Lam proteins does not inhibit the bidirectional transport of newly synthesized ergosterol between the ER and PM, sterol organization at the PM is altered [30]. If Osh and Lam proteins catalyze ER-PM sterol transport, then they must do so redundantly with each other and/or with additional STPs yet to be identified. Our results address this point.

In addition to their proposed role in sterol homeostasis, ER-PM MCSs are known to be involved in phospholipid biosynthesis and turnover. Phosphatidylcholine (PC) is synthesized via Cho2 and Opi3-mediated methylation of phosphatidylethanolamine (PE). Opi3 is an ER-localized membrane protein in yeast that has been proposed to act in *trans* at ER-PM MCSs to convert PM-localized PE to PC [31]. In the absence of Opi3 function (either through lack of the enzyme or disruption of ER-PM MCSs), cells rely on the Kennedy pathway, through which PC is synthesized from choline taken up from the growth medium. It has been proposed that phosphoinositide turnover also occurs at ER-PM MCSs, where the ER-localized PI4P phosphatase Sac1 may act in *trans* to turn over PI4P synthesized in the PM [5]. These examples highlight the possibility that ER-PM MCSs may contribute to a wide range of reactions that underlie cellular phospholipid homeostasis.

**Fig 1:**
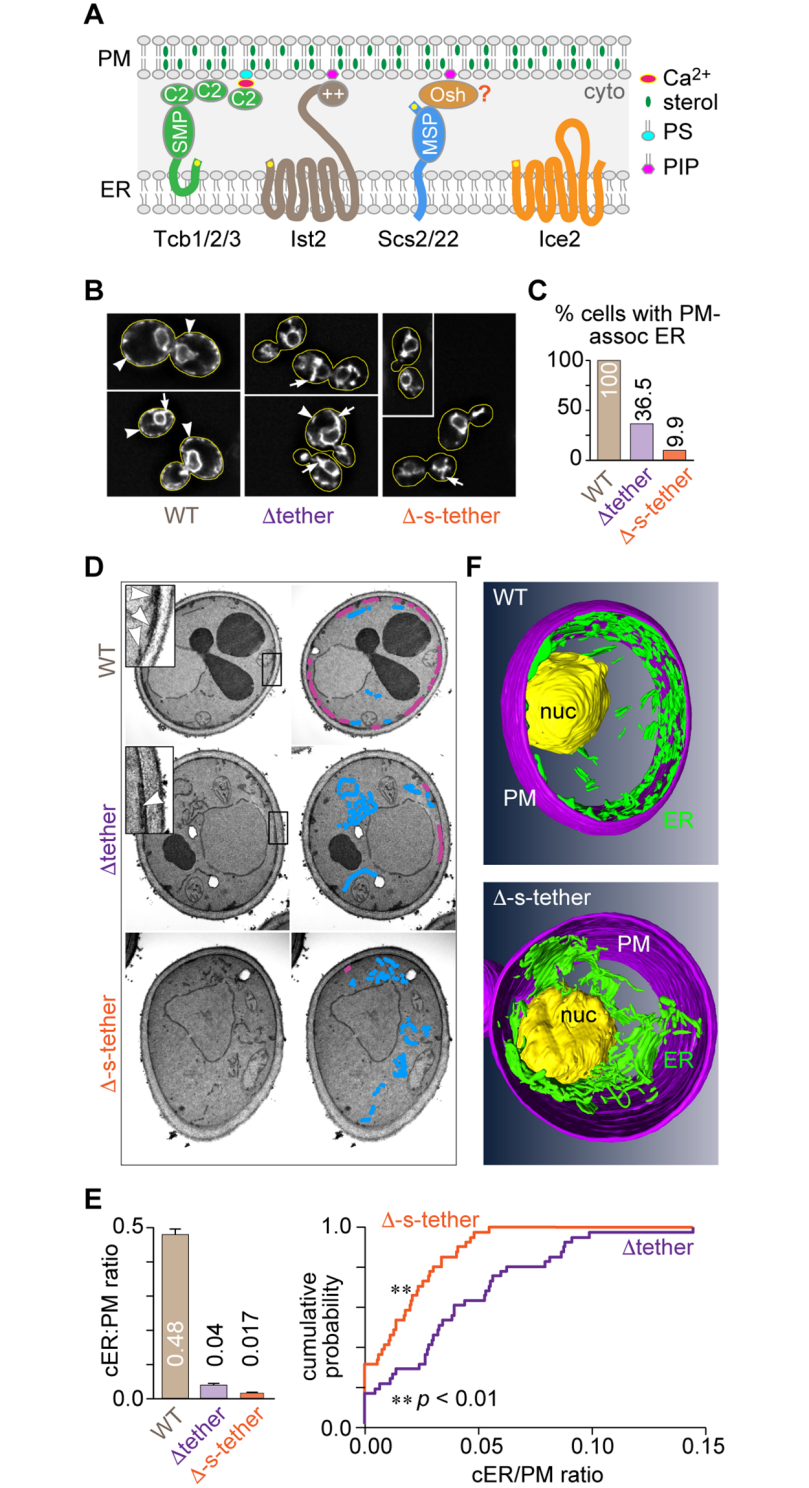
Quantitative disruption of ER-PM contacts in Δ-s-tether cells. **A.** Proposed topology of ER membrane proteins involved in establishing ER-PM contact sites. The yellow dot indicates the N-terminus of the protein. Tcb1/2/3 associate with the PM through lipid-binding C2 domains and possess an SMP domain that is implicated in the exchange of phospholipids and diacylglycerol between the PM and ER [38]. Ist2 is a member of the TMEM16 family of ion channels and lipid scramblases. It interacts with the PM via its C-terminal PI(4,5)P_2_-binding polybasic region (++) [34]. The yeast VAPs Scs2/22 interact with the PM indirectly, likely through Osh proteins (or other proteins) that possess an FFAT motif capable of binding to the MSP domain of the VAPs [41–43] and a PH domain that interacts with phosphoinositides at the PM. Ice2 facilitates cER inheritance from the mother cell along the PM into the bud [33, 44]; the *ICE2* and *SCS2* genes have a negative genetic interaction [31]. **B.** Representative images of WT (SEY6210), Δtether (ANDY198), and Δ-s-tether (CBY5838) cells expressing the ER marker RFP-ER (pCB1024). The PM-associated ER (arrowheads) at the cell cortex (outlined in yellow) observed in WT cells was largely absent in the Δtether and Δ-s-tether mutants, which exhibited prominent extranuclear cytoplasmic ER (arrows). Scale bar = 2 μm. **C.** Quantification of RFP-ER localization comparing the percentage of WT and mutant cells exhibiting cER-PM fluorescence (*n* > 140 cells). **D.** Electron micrographs of WT, Δtether, and Δ-s-tether cells. Inserts correspond to magnifications of boxed regions at the cell cortex, showing PM-associated ER (arrowheads). Cortical PM-associated ER (magenta) was reduced in Δtether cells and all but eliminated in Δ-s-tether cells. Extranuclear/cytoplasmic ER (blue) is prominent in the tether mutant cells. **E.** Left: quantification of cER expressed as a ratio of the length of PM-associated ER per circumference of PM in each cell (*n* = 41 cells; bars are mean ± SEM). Right: comparison of the cumulative distribution of cER/PM ratios for Δtether (purple) versus Δ-s-tether (red) shows a significant decrease in cER across the entire population of cells. ** *p* < 0.01 by Kolmogorov-Smirnov and Wilcoxon Rank Sum tests. See S1 Fig for further details. **F.** Models of the 3D organization of ER membranes within WT and Δ-s-tether cells constructed from sections imaged by focused-ion beam tomography: cER (green) in association with the PM (magenta); nuclear ER (yellow). Numerical data presented in this figure may be found in S1 Data. Δ-s-tether, Δ-super-tether; cER, cortical ER; C2, protein kinase C conserved region 2; ER, endoplasmic reticulum; FFAT; two phenylalanines in an acidic tract; MSP, major sperm protein; nuc, nucleus; Osh, OSBP homologue; PH, Pleckstrin homology; PIP, phosphatidylinositol phosphate; PM, plasma membrane; PS, phosphatidylserine; RFP, red fluorescent protein; SMP, synaptotagmin-like mitochondrial-lipid-binding protein; Tcb, tricalbin; VAMP, vesicle-associated membrane protein; VAP, VAMP-associated protein; WT, wild type.

In yeast, about 45% of the PM retains a closely associated cortical ER (cER) membrane [5, 32], and this association requires a number of tethering proteins that staple the ER and PM together [5, 31, 33–35]. Six ER-PM tethering proteins are currently known (Fig 1A): the three tricalbins (Tcb1–3), which are yeast homologues of the extended synaptotagmin (E-Syt) family of membrane tethers; Ist2, a member of the TMEM16-anoctamin family of ion channels and phospholipid scramblases; and the yeast vesicle-associated membrane protein (VAMP)-associated protein (VAP) homologues Scs2 and Scs22. Several of these tethers appear to be Ca^2+^ regulated in mammalian cells, and from their embedded location within the ER membrane, a number of them make contact with the PM through associations with phosphoinositides and/or other phospholipids [6, 8, 36–40]. By eliminating all six of these tethering proteins, Manford and colleagues [5] created Δtether yeast cells, in which large sections of the PM are devoid of cortically associated ER membrane. However, as these authors noted, additional unknown tethers must still exist in Δtether cells, given that small regions of cER were still associated with the PM [5]. Consistent with this conclusion, the localization of Lam1–Lam4 close to the PM near presumptive MCSs is unaffected in Δtether cells [30]. These results suggest that elimination of the six tether proteins is not sufficient to remove all ER-PM contacts and that additional proteins/mechanisms must exist to account for the remaining ER-PM association.

In order to eliminate residual cER in Δtether cells, we focused on Ice2, an integral ER membrane protein (Fig 1A) with established roles in cER inheritance [33, 44], ER-PM contact [31, 33], phospholipid synthesis from stored neutral lipid [31, 45], and ER quality control [46]. Ice2 was first shown to facilitate cER redistribution and inheritance along the PM from mother cells into daughter buds [44]. In cells lacking both *ICE2* and *SCS2*, cER association at the PM is disrupted more than for each single mutant [31, 33]. The defect in ER-PM membrane association in *scs2*Δ*ice2*Δ cells is linked to dysfunctional PC synthesis, likely because the Opi3 methyltransferase is no longer able to act on its PM-localized lipid substrate in *trans* at contact sites [31]. When cells enter stationary phase, Ice2 has another function, in which it generates a bridge between the ER and lipid droplets [45]. This membrane attachment has been proposed to play a role in channeling droplet-generated diacylglycerol (DAG) to the ER for phospholipid synthesis when cells resume growth [45]. Curiously, the ER-associated degradation (ERAD) substrate carboxypeptidase Y* (CPY*) is stabilized in *ice2*Δ cells compared with wild-type (WT) cells, pointing to a direct or indirect role for Ice2 in ER-associated degradation [46]. We reasoned that because of its various ER functions, specifically including the generation of ER-PM contacts during mitosis, Ice2 might account for the residual cER in Δtether cells.

If ER-PM contact is necessary for non-vesicular sterol transfer, the rate of ER-PM sterol exchange and/or PM sterol organization would be inhibited by the elimination of all ER-PM MCSs. Likewise, if MCSs serve as regulatory interfaces to coordinate pathways for phospholipid metabolism in the ER and PM, then removing ER-PM MCSs would be predicted to alter cellular phospholipid profiles. We now report that disruption of *ICE2* in Δtether cells sharply reduces ER-PM associations to the predicted frequency of randomly finding untethered ER in the vicinity of the PM. The availability of these Δ-super-tether (Δ-s-tether) cells now permits direct tests of hypotheses concerning how ER-PM MCSs impact non-vesicular sterol exchange and inter-membrane lipid regulation.

We now report that the bidirectional movement of sterols between the ER and PM is unaffected in Δ-s-tether cells, indicating clearly that the sterol transfer machinery in yeast is either absent from or not uniquely localized to ER-PM MCSs. Nonetheless, sterol pools within the PM bilayer of Δ-s-tether cells are dramatically altered, and the rate of transbilayer sterol movement within the PM is slowed. We discovered that these defects were associated with changes in the organization and composition of PM lipids and could be largely reversed by supplementing cells with choline or by expressing a nonspecific artificial ER-PM tether. Phospholipid dysregulation in the PM was revealed by changes in the levels of sphingolipids and other PM lipids, as well as by the accumulation of PI4P at the PM of mother Δ-s-tether cells. Interestingly, Δ-s-tether cells were inviable when they also lacked Osh4 or Sac1. After testing the associated roles of Osh6 and the ER-membrane association of Sac1, we conclude that Osh4 and ER-PM MCSs are redundant regulators of PI4P and phospholipid homeostasis. Finally, we found that ER-PM MCS formation is responsive to cellular sterol levels, whereby the tether protein Tcb3 is induced in sterol-depleted cells, resulting in a dramatic increase in membrane association. These results suggest that ER-PM contact sites are dynamic interfaces that adjust and respond to lipid metabolism to maintain PM composition and organization.

## Results

### Yeast cells without ER-PM contact sites

Despite the dramatic reduction in ER-PM contact sites caused by the elimination of six tether proteins, the extent of cER in Δtether cells [5] is both significant and heterogeneous, with >35% of the cells possessing fluorescently labeled ER in the vicinity of the PM (Fig 1B and 1C) and individual cells displaying as much as 20% of the average cER found in WT cells (Fig 1D and 1E and S1A Fig). Residual cER in Δtether cells is also evinced by the cortical localization of green fluorescent protein (GFP)-tagged Ysp2/Lam2/Ltc4 (hereafter called Lam2; S2 Fig) and other Lam proteins [30]. These observations suggest that there are additional mechanisms for generating ER-PM association [8]. Because the gene encoding the ER membrane protein Ice2 (Fig 1A) has a negative genetic interaction with *SCS2*, and Ice2 plays roles in maintaining cER structure and mediating the inheritance of cER from mother cells into daughter buds [33, 44], we hypothesized that Ice2 may contribute to ER-PM association and that its presence in Δtether cells could account for the residual cER seen in these cells.

To explore this possibility, we used confocal fluorescence microscopy to determine the subcellular distribution of Ice2-GFP in Δtether cells (S3 Fig). Ice2-GFP was observed throughout the ER, including cytoplasmic strands radiating from nuclear ER towards the cell periphery (S3A Fig). Notably, Ice2-GFP fluorescence was observed at the cell cortex, visualized by co-expressing the PM marker red fluorescent protein (RFP)-Ras2 (S3A Fig). Optical sections focused at the cell surface showed a concentration of Ice2-GFP fluorescence at remaining ER cortical spots that were visualized using the fluorescent pan-ER marker RFP-ER (dsRED-*SCS2*^220–244^) (S3B Fig). This localization pattern resembles that of Scs2 and differs from that of the Tcbs and Ist2 that are restricted to ER-PM MCS spots [5, 35, 42, 47]. These data suggest that Ice2 is correctly localized to contribute to ER-PM tethering. To eliminate residual cER in Δtether cells, we therefore deleted *ICE2* in tandem with the other Δtether mutations. We predicted that the resulting Δ-s-tether cells would be largely devoid of ER-PM contact sites and this was indeed the case.

We confirmed the near absence of PM-associated cER in Δ-s-tether cells as follows. First, expression of the pan-ER marker RFP-ER revealed that, unlike WT and Δtether cells, in which cortical fluorescence was observed in 100% and >35% of cells, respectively, fewer than 10% of Δ-s-tether cells had fluorescence at the cell cortex (Fig 1B and 1C). Whereas cortical fluorescence in WT cells and many Δtether cells occurred in the form of linear strands running parallel to the cell perimeter in equatorial views (Fig 1B, arrowheads), the occasional fluorescence seen at the cortex of a small fraction (<10%) of Δ-s-tether cells was in the form of punctae, possibly corresponding to the ends of ER tubules or coincidental positioning of the ER near the PM in the focal plane chosen for imaging (Fig 1B, arrows). Second, whereas GFP-Lam2 is localized exclusively in cortical punctae in WT and Δtether cells (about 15 cortical punctae per cell, on average, for both strains), cortical expression of Lam2 is considerably reduced in Δ-s-tether cells (about four cortical punctae per cell, on average; S2A and S2B Fig), even though the expression level of the protein is unaffected (S2C Fig). Third, cER association along the PM in Δ-s-tether cells was all but absent, as quantified by measuring the cER/PM length ratio in equatorial views of individual cells obtained by transmission electron microscopy (Fig 1D). The average cER/PM ratio was 0.48 and 0.04 in WT and Δtether cells, respectively [5], but only 0.017 in Δ-s-tether cells (Fig 1E). The decrease in the cER/PM ratio in Δ-s-tether versus Δtether cells was statistically significant (Fig 1E, right panel), representing not only an approximately 60% lower average value but also a considerable tightening of the distribution of cER/PM values (Fig 1E and S1A Fig). Finally, we generated 3D models of WT and Δ-s-tether cells by reconstructing images obtained with a focused ion beam–scanning electron microscope (FIB-SEM). These models (Fig 1F) illustrate that the extensive cER coverage of the PM in WT cells is clearly absent in Δ-s-tether cells, where a spaghetti-like accumulation of cytoplasmic tubular ER is observed instead. We estimate that the low amount of cER in Δ-s-tether cells (Fig 1E and S1B Fig) can be accounted for by the random chance of finding untethered ER at the cortex (Materials and methods).

**Fig 2:**
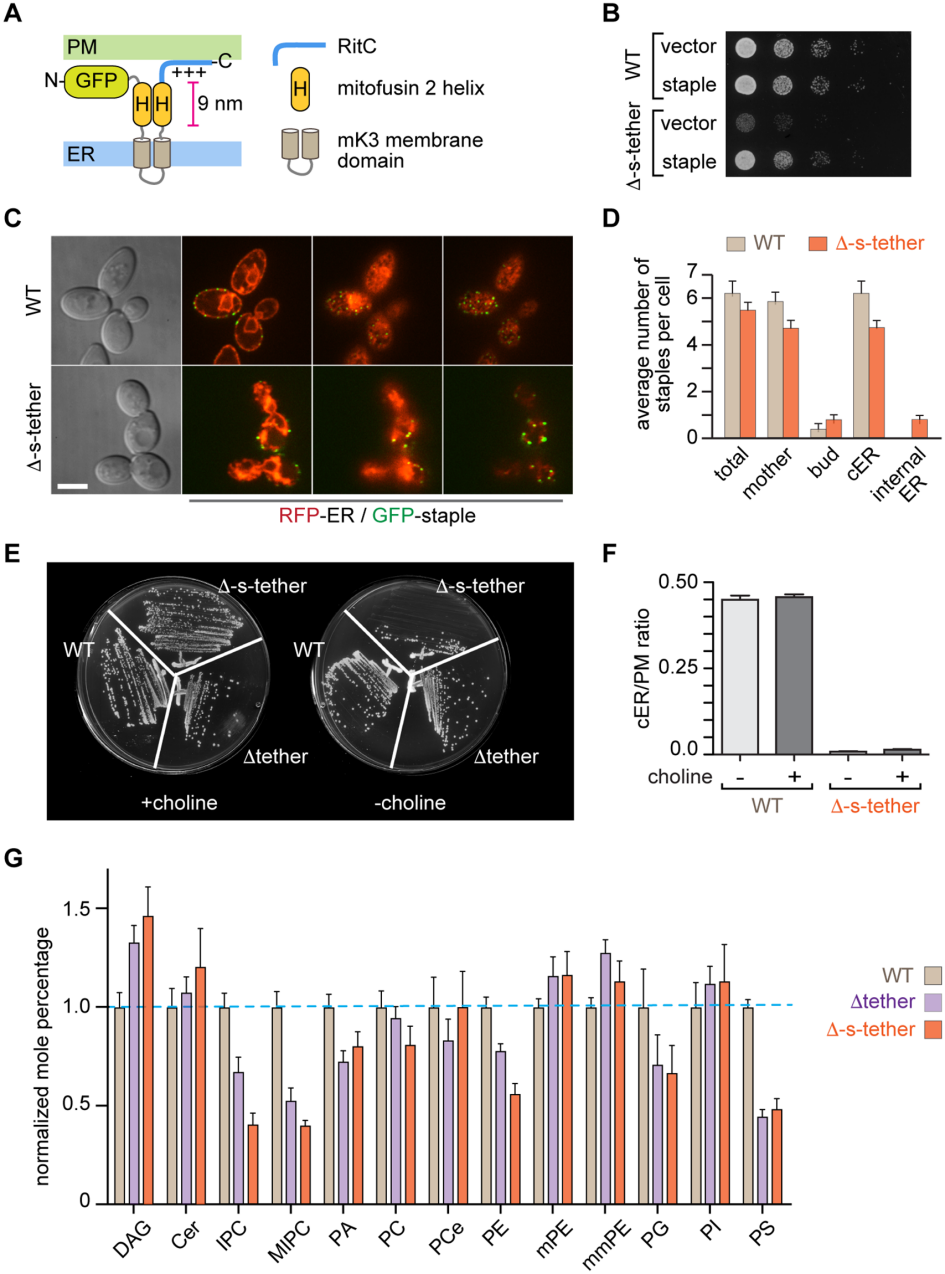
Slow growth of Δ-s-tether cells is rescued by expression of an artificial ER-PM tether or choline. **A.** The “ER-PM staple" has a modular architecture consisting of an N-terminal GFP, an ER anchor comprising two transmembrane domains and a lumenal loop from herpes virus (MVH68) mK3 E3 ubiquitin ligase, two helices from mitofusin 2 that are predicted to adopt an antiparallel arrangement about 9 nm long, and the polybasic domain from RitC that targets the PM. **B.** Tenfold serial dilutions of WT (SEY6210) and Δ-s-tether (CBY5838) cells, transformed with either the vector control (YCplac111) or a plasmid expressing the artificial staple (pCB1185), spotted on solid growth medium, and incubated for 2 d at 30 °C. **C.** DIC images of WT and Δ-s-tether cells and the corresponding spinning disc confocal fluorescence microscopy images showing the colocalization of RFP-ER (pCB1024) and the GFP-marked artificial staple (pCB1185) at three different optical focal planes. Scale bar = 5 μm. **D.** Quantification of the staple distribution within mother and buds and at cER versus internal cytoplasmic ER. **E.** Choline-dependent growth of Δ-s-tether cells. WT, Δtether (ANDY198), and Δ-s-tether (CBY5838) cells were streaked onto solid growth medium supplemented with 1 mM choline chloride, as indicated, and incubated for 3 d at 30 °C. **F.** Quantification of ER-RFP localization in WT and Δ-s-tether cells, with and without 1 mM choline, represented as a ratio of the length of PM-associated ER per circumference of PM in each cell (*n* > 50 cells; error bars represent SEM). **G**. Lipid composition of WT, Δtether, and Δ-s-tether cells represented as a normalized mole percentage relative to WT (set to 1.0). The data represent the mean ± SEM derived from the analysis of five independent samples. Numerical data presented in this figure may be found in S1 Data. Δ-s-tether, Δ-super-tether; cER, cortical ER; DAG, diacylglycerol; DIC, differential interference contrast; ER, endoplasmic reticulum; GFP, green fluorescent protein; IPC, inositol-phosphoceramide; MIPC, mannosylinositol phosphoceramide; mmPE, dimethyl PE; mPE, monomethyl PE; PA, phosphatidic acid; PC, phosphatidylcholine; PCe, ether phosphatidylcholine; PE, phosphatidylethanolamine; PG, phosphatidylglycerol; PI, phosphatidylinositol; PM, plasma membrane; PS, phosphatidylserine; RFP, red fluorescent protein, RitC; C-terminal polybasic region from mammalian Rit1; WT, wild type.

Δ-s-tether cells grow normally on rich media but poorly on minimal media (Fig 2B and 2E), suggesting that the lack of ER-PM contact sites disrupts cell metabolism. If this were indeed the case, then an artificial ER-PM tethering protein might allow the cells to grow normally. Several of the natural ER-PM tethers, e.g., Tcb1–3 and Ist2 (Fig 1A), have a modular architecture, and this design principle was used to assemble an artificial tether ("ER-PM staple") from unrelated non-yeast proteins. As building blocks for the ER-PM staple, we used (i) two ER-anchoring *Trans*-membrane domains from herpes virus mK3 E3 ubiquitin ligase, (ii) extended helices from mammalian mitofusin 2 to span the gap between the PM and ER, and (iii) the C-terminal polybasic region from mammalian Rit1 (RitC) that targets the PM (Fig 2A). We fused GFP to the *N*-terminus in order to visualize the ER-PM staple in cells. Expression of the ER-PM staple from the yeast actin promoter largely rescued the growth defect of Δ-s-tether cells cultured on solid medium (Fig 2B), indicating that the artificial staple is a functional substitute for the endogenous tether proteins. Fluorescence microscopy revealed that the ER-PM staple localizes to cER in both WT and Δ-s-tether cells, consistent with the idea that it generates ER-PM contact sites, albeit fewer than endogenous tethers (Fig 2C and 2D). The overall distribution of the ER-PM staples was similar in WT and Δ-s-tether cells, although the staples in Δ-s-tether cells aggregated in larger spots with greater fluorescence. The finding that a wholly heterologous construct can replace endogenous tether proteins in rescuing the poor growth of Δ-s-tether cells indicates importantly that the tethers (Fig 1A) perform a nonspecific bridging function relevant to cell growth that is exclusive of any tether-specific activities. A further conclusion from this result is that the proposed lipid transfer function of the synaptotagmin-like mitochondrial-lipid-binding protein (SMP) domains of the Tcbs (Fig 1A) [48] is not required for cell growth, consistent with observations in HeLa cells and mice lacking E-Syts [49, 50].

### ER-PM contacts have multifactorial effects on several lipid biosynthetic pathways

Why would the absence of ER-PM tethers cause cells to grow slowly, with growth rescue being achieved by an artificial tether? Tavassoli and colleagues [31] suggested that the ER-anchored phospholipid methyltransferase Opi3 acts at ER-PM contact sites in *trans* to generate a pool of PC at the PM that is necessary for growth. If Opi3 is unable to act on the PM, as would be expected for cells lacking ER-PM contact sites, then a choline supplement must be provided to generate the necessary PC via the Kennedy pathway [31]. Indeed, we found that Δ-s-tether cells achieve normal growth when the medium is supplemented with choline (Fig 2E). Importantly, the extent of cER was not detectably different in choline-grown Δ-s-tether cells (Fig 2F). We conclude that choline supplementation bypasses the requirement for ER-PM contact sites to support cell growth.

Consistent with these findings, the deletion of either *OPI3* or *CHO2* in Δ-s-tether cells severely exacerbated the growth defect of the cells unless choline was provided (S4A Fig). However, in the absence of choline, Opi3 overexpression effectively suppressed the choline-dependent growth defect of Δ-s-tether cells (S4B Fig), potentially by providing an alternative route to generate PC pools at the PM. Unlike choline supplementation to the growth medium, the addition of ethanolamine or inositol, which promote PE and PI synthesis, respectively, did not rescue Δ-s-tether growth defects (S5 Fig).

The ability of choline to rescue the poor growth of Δ-s-tether cells, and the functional requirements of Δ-s-tether cells for *CHO2* and *OPI3*, suggested that PC synthesis/levels might be dysregulated in these cells. Surprisingly, whole cell lipidomics (Fig 2G) revealed that PC levels were only about 20% lower in Δ-s-tether cells compared with WT cells, but the relative amounts of a number of other lipids, notably PE, phosphatidylserine (PS), and the yeast sphingolipids inositol-phosphoceramide (IPC) and mannosylinositol phosphoceramide (MIPC), were considerably reduced. Reduced levels of these lipids were also found in Δtether cells that grow almost as well as WT cells, but the lipid compositional effects were generally more pronounced in Δ-s-tether cells. For example, we found the mole percentage of IPC content in Δtether cells to be about 67% of that in WT cells, whereas in Δ-s-tether cells, the level of this lipid fell to about 40% of that in WT cells. Increases in some lipids were also measured, most notably DAG, which was 1.3-fold higher in Δ-s-tether compared with WT cells. These results suggest a possible threshold effect, in which the lipid compositional changes in Δ-s-tether cells have a severe impact on growth, whereas the somewhat lesser changes in Δtether cells do not.

Comparison of the lipid composition of Δ-s-tether cells cultured with or without choline supplementation revealed changes that could be predicted based on the deployment of the Kennedy pathway because of the availability of choline (S6A Fig). Thus, PC and PS levels increased in the choline-supplemented cells, bringing the levels of these lipids closer to those in WT, whereas levels of mono- and dimethyl-PE (mPE and mmPE, respectively) fell. Choline supplementation of Δ-s-tether cells also resulted in an increase in MIPC levels, although other sphingolipids and their precursors were only slightly affected. Unlike lipid compositional changes seen upon choline addition, rescue of Δ-s-tether growth defects by the artificial tether indicated a different mechanism. The lipidomic profile of Δ-s-tether cells expressing the artificial tether was more consistent with a restoration of normal phospholipid synthesis through the cytidine diphosphate diacylglycerol (CDP-DAG) pathway (S6B Fig). The artificial tether increased PS, PI, and mmPE levels, although levels of PC were not appreciably changed from Δ-s-tether cells cultured without choline. The artificial tether also affected storage lipids: esterified ergosterol and triacylglycerol (TG) showed especially large increases as a proportion compared to WT. To our surprise, expression of the artificial tether in Δ-s-tether cells did not restore levels of sphingolipids or their immediate precursors. We conclude that the molecular basis of growth rescue in Δ-s-tether cells by choline and the artificial tether is multifactorial and is likely finely tuned to the precise pools and relative abundance of several lipids, depending on the mode of suppression.

### Retrograde transport of sterols is defective in Δ-s-tether cells

**Fig 3:**
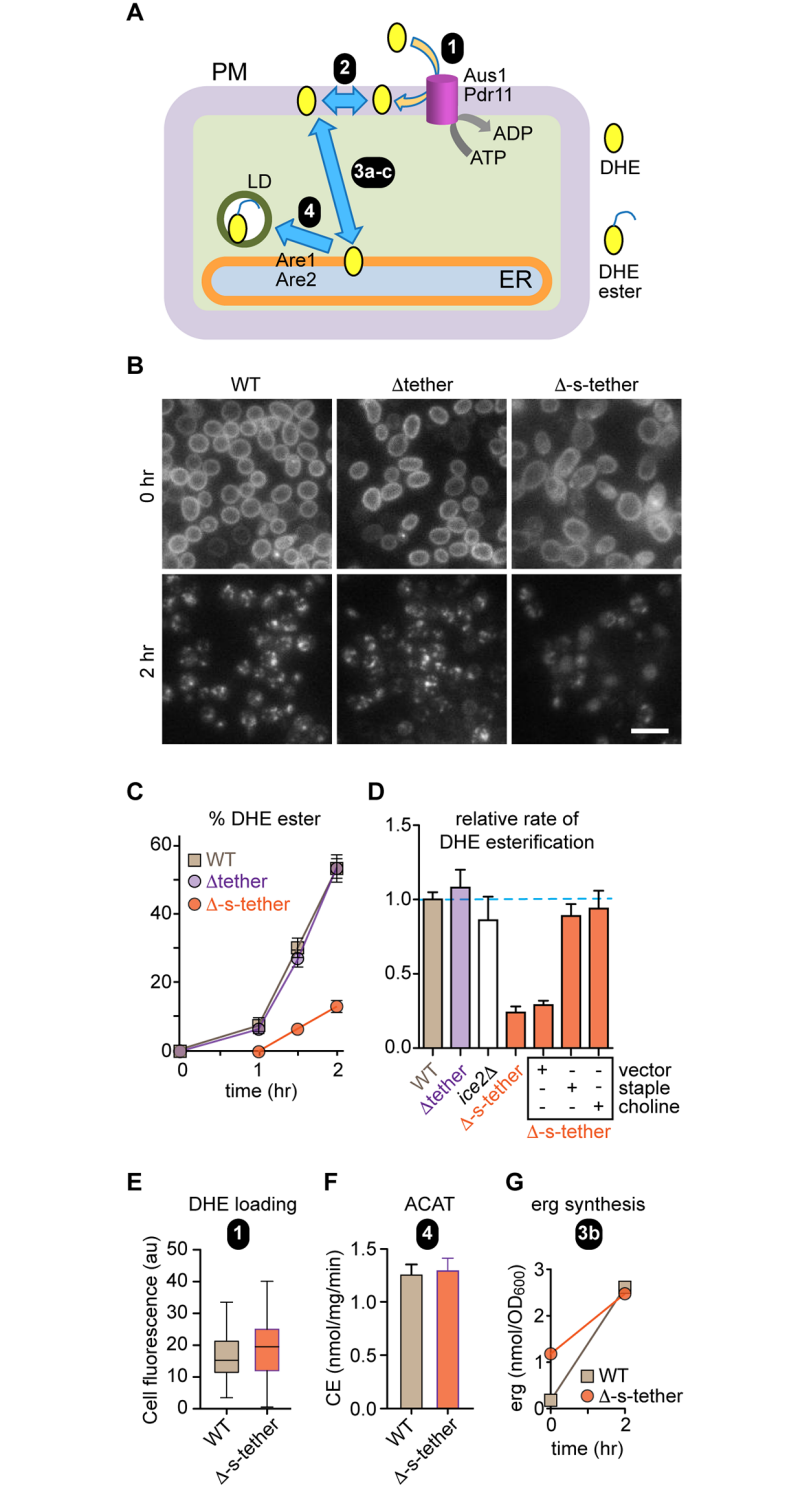
Retrograde transport of exogenously supplied DHE is slowed about 4-fold in Δ-s-tether cells; rescue by expression of an artificial ER-PM tether or choline. **A.** Schematic illustration of the retrograde sterol transport assay. The assay measures transport-coupled esterification of exogenously supplied DHE. Cells are incubated with DHE for 36 h under hypoxic conditions to load the sterol into the PM (step 1, mediated by the ABC transporters Aus1 and Pdr11). Further incubation (chase period) after exposing the cells to air results in the exchange of DHE between pools in the PM (step 2) and its transfer to the ER (step 3), where it is esterified (step 4) by the sterol esterification enzymes Are1 and Are2. DHE esters that are sequestered in LDs. **B.** Representative images of WT, Δtether, and Δ-s-tether cells obtained immediately after DHE loading (chase time = 0 h) and 2 h after incubation under aerobic conditions. The punctae seen in the 2 h chase images correspond to LDs. Scale bar = 10 μm. **C.** DHE esters were quantified at different times during the aerobic chase period by analyzing hexane/isopropanol extracts of the cells by HPLC equipped with an in-line UV detector. The data are represented as percentage of DHE ester recovered (= DHE ester/(DHE + DHE ester)). Linear regression of the data points between 1 and 2 h indicates relative slopes of 1 (for WT and Δtether cells) and 0.24 ± 0.05 for Δ-s-tether cells (also see panel D). **D.** Transport-coupled esterification of exogenously supplied DHE. The bar chart presents the mean ± SEM (*n* = 3) of the relative rate of DHE esterification after the 1 h lag period at the start of the aerobic chase. The mean esterification rate for WT cells is set at 1.0. **E.** Incorporation of DHE into the PM (corresponding to step 1 in panel A), quantified using fluorescence images acquired immediately after the hypoxic incubation period. The area, integrated fluorescence, and the CTCF were calculated for individual cells using Image J. At least 40 cells were analyzed. CTCF = integrated density − (area of selected cell × mean fluorescence of background reading). The box and whiskers plot shows the mean of the measurements, with whiskers ranging from the minimum to the maximum value measured. **F.** Microsomes from WT and Δ-s-tether cells were assayed for their ability to esterify [^3^H]cholesterol (supplied as a complex with methyl-β-cyclodextrin) on addition of oleoyl-CoA. Esterification, assessed by organic solvent extraction and thin layer chromatography, proceeded linearly for at least 10 min. The bar chart shows the mean ± SEM (*n* = 4) of ACAT activity as the rate of production of CE per mg microsomal protein per minute. This measurement corresponds to step 4 in panel A. **G.** The amount of ergosterol in WT and Δ-s-tether cells (nmol per OD_600_ of cell suspension) was measured by lipid extraction and HPLC at the start and end of the aerobic chase period. This measurement corresponds to step 3a in panel A (see text for details). Numerical data presented in this figure may be found in S1 Data. Δ-s-tether, Δ-super-tether; ABC, ATP-binding cassette; ACAT, acetyl-CoA acetyltransferase; ADP, adenosine diphosphate; CE, cholesteryl ester; CoA, coenzyme A; CTCF, corrected total cell fluorescence; DHE, dehydroergosterol; ER, endoplasmic reticulum; HPLC, high-performance liquid chromatography; LD, lipid droplet; PM, plasma membrane; UV, ultraviolet; WT, wild type.

To determine whether ER-PM contact sites play a role in sterol exchange between the two membranes, we compared the rate of retrograde transport of dehydroergosterol (DHE) from the PM to the ER in WT, Δtether, and Δ-s-tether cells using a previously described assay (Fig 3A) [21, 51]. DHE is a fluorescent sterol that is widely used as a reporter of intracellular sterol transport and distribution [52]; it is particularly appropriate as a sterol reporter in yeast cells, as it is closely related to ergosterol and as effective as ergosterol in supporting the growth of *hem1Δ* cells that cannot synthesize sterols [21]. To load DHE into the PM, the cells are incubated under hypoxic conditions to overcome "aerobic sterol exclusion," which represses endogenous sterol synthesis in favor of exogenous sterol import. When the DHE-loaded cells are transferred to aerobic conditions, ergosterol synthesis resumes and DHE is displaced from the PM. On reaching the ER, DHE becomes esterified by ER-localized sterol acyltransferases. The extent of esterification—detected by the appearance of lipid droplets containing fluorescent DHE or direct measurement of DHE esters by high-performance liquid chromatography (HPLC) analysis of lipid extracts from the cells [21]—provides a measure of retrograde transport.

At the start of the chase period, DHE fluorescence was observed as a “ring stain” in WT, Δtether, and Δ-s-tether cells (Fig 3B), indicating insertion of the fluorescent sterol into the PM [21, 53]. After a 2 h incubation (“chase”) under aerobic conditions, fluorescence was concentrated in lipid droplets in WT and Δtether cells, but the same punctate fluorescence was not observed in Δ-s-tether cells (Fig 3B). To quantify retrograde transport, the amount of imported DHE that was converted into DHE esters was measured at different times following the aerobic chase (Fig 3C). DHE esterification proceeds linearly after a lag period of about 1 h, during which the cells adapt to aerobic conditions, allowing resumption of ergosterol synthesis [21]. Compared to WT or Δtether cells, we observed an approximately 4-fold decrease in the rate of transport-coupled esterification of DHE in Δ-s-tether cells (Fig 3C and 3D). This reduction in esterification rate was not seen in the progenitor strains Δtether or *ice2*Δ and could be restored to WT levels by expressing the ER-PM staple, or by growing the cells in choline (Fig 3D, S7A and S7B Fig). The latter result (i) suggests that sterol transport between the PM and ER does not depend on ER-PM MCSs, as these structures are equally absent in Δ-s-tether cells grown with or without choline (Fig 2F), and (ii) argues against a recent proposal [54] that the sterol acyl transferases Are1 and Are2 act in *trans* at ER-PM MCSs, directly receiving sterols from the ATP-binding cassette (ABC) transporters Aus1 and Pdr11, thereby eliminating the need for STP-mediated sterol transport between the PM and ER.

Transport-coupled esterification of DHE is a complex process that can be separated into a series of discrete mechanistic steps (Fig 3A): (1) insertion of DHE into the PM, requiring the ABC transporters Aus1 and Pdr11; (2) equilibration of DHE amongst PM sterol pools, e.g., pools located in the outer and inner leaflets; (3) non-vesicular transport of DHE from the cytoplasmic face of the PM to the ER (3a), a process that requires resumption of ergosterol synthesis (3b) as the cells recover from hypoxia, and transport of ergosterol to the PM (3c); and, finally, (4) esterification of DHE at the ER by the acetyl-CoA acyltransferase (ACAT) enzymes Are1 and Are2. Defects in one or more of these steps could account for the slowdown in DHE esterification seen in Δ-s-tether cells. We verified that DHE loading (step 1) (Fig 3E) and ACAT activity (step 4) (Fig 3F) were similar in WT and Δ-s-tether cells grown in the absence of choline, and the same was true when the cells were grown in the presence of choline (S7 Fig [panels C and D]). However, the level of endogenous ergosterol in Δ-s-tether cells at the start of the aerobic chase was higher than in WT cells on a per cell basis, although it reached the same value at the end of the chase, indicating that ergosterol resynthesis (step 3b) occurs normally (Fig 3G). No difference between ergosterol content and resynthesis was seen when the cells were grown in the presence of choline (S7E Fig). As ergosterol synthesis is largely abolished under hypoxic conditions, the ergosterol content of each cell diminishes with each cell division and is replaced in our protocol by DHE. Because Δ-s-tether cells grow slowly in the absence of choline, the ergosterol “wash-out” is less complete for these cells than for WT cells. The presence of a significant amount of residual ergosterol in Δ-s-tether cells at the start of the aerobic chase could conceivably reduce the rate at which newly synthesized ergosterol is able to displace DHE from the PM, resulting in an apparently slower DHE esterification rate and obscuring information on whether sterol transport between the PM and ER is indeed affected. Thus, our results suggest that the slow rate of esterification observed in Δ-s-tether cells could be due to a defect in steps 2 (DHE equilibration within the PM) and/or 3 (sterol [DHE and ergosterol] exchange between the PM and the ER) (Fig 3A).

### Bidirectional exchange of newly synthesized ergosterol between the ER and PM is normal in Δ-s-tether cells

**Fig 4:**
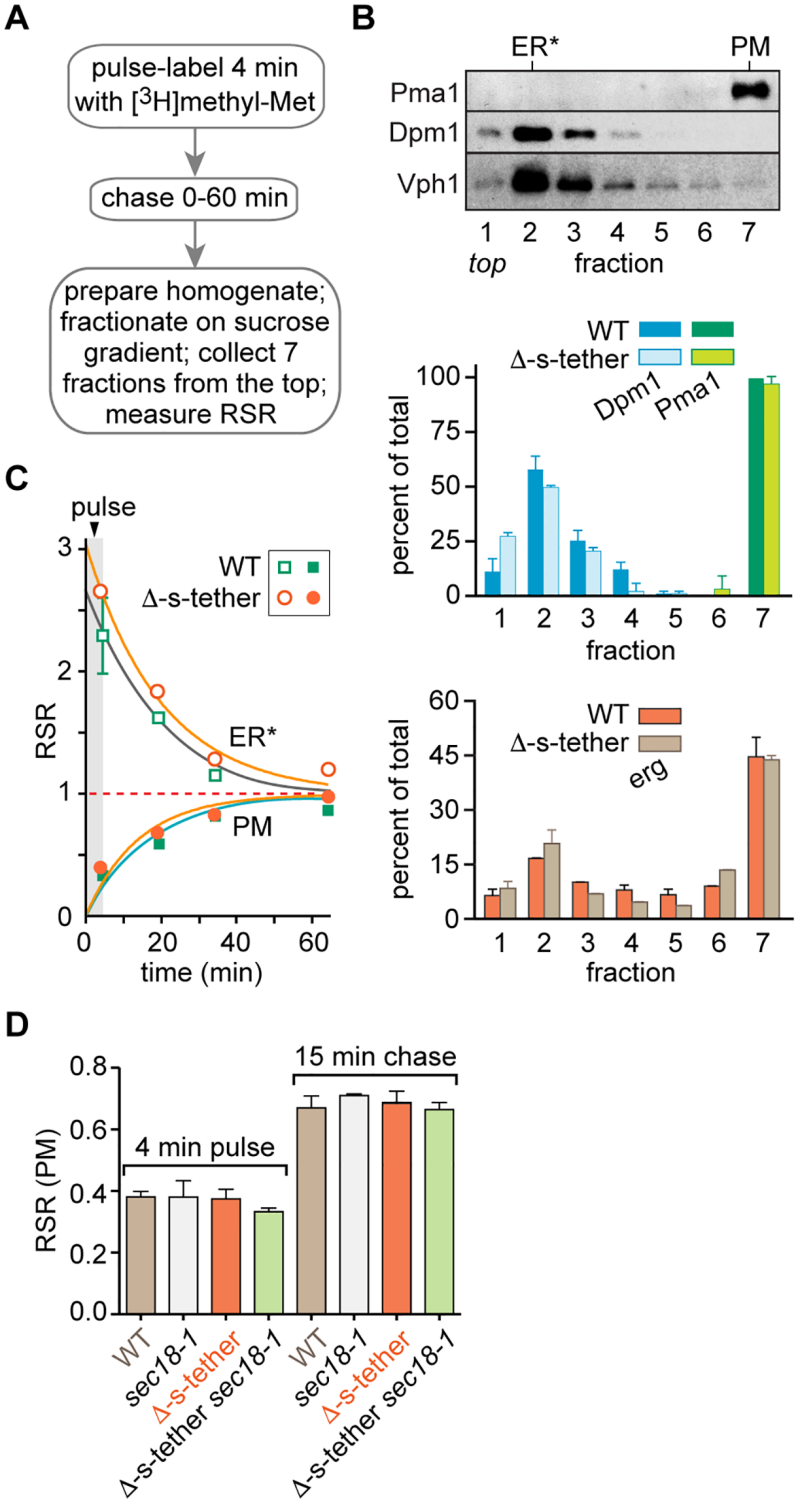
Bidirectional transport of ergosterol from the ER to the PM is unaffected in Δ-s-tether cells. **A.** Outline of the transport assay. **B.** Characterization of subcellular fractions. Top, immunoblots using antibodies against Pma1 (PM), Dpm1 (ER), and Vph1 (vacuole). Fraction 2 is designated ER* to indicate that it contains membranes in addition to ER membranes. Data correspond to fractionation of a homogenate of WT cells prepared at the end of the labeling pulse. Middle, quantification of Dpm1 and Pma1 in fractions prepared from homogenates of WT and Δ-s-tether cells taken after a 30 min chase period. The blots were quantified by ImageJ. Bottom, quantification of ergosterol in the different fractions from the middle panel. **C.** WT and Δ-s-tether cells were processed as in panel A. The SR of ergosterol in each fraction ([^3^H]ergosterol [cpm] ÷ ergosterol mass) was normalized to the SR of the total homogenate at each time point to obtain an RSR. The figure shows RSR versus time (t = 0 min is the start of the labeling pulse). The lower portion of the graph (solid symbols) is based on 3–4 independent experiments; the upper portion (open symbols) is based on 2–5 independent experiments. The lines are mono-exponential fits of the data that plateau at RSR = 1. **D.** Transport of ergosterol in Δ-s-tether cells with block in vesicular transport. Mid-log cultures of WT, *sec18-1*^*ts*^ (CBY2859), Δ-s-tether (CBY5838), and Δ-s-tether *sec18-1*^*ts*^ (CBY5851) cells were grown at 24 °C, shifted to the restrictive temperature of 37 °C for 20 min, pulse-labeled with [^3^H]methyl-methionine for 4 min and chased for 15 min at the same temperature. The bar chart shows the RSR of the PM fraction from samples taken at the end of the pulse and chase periods. Data are mean ± SEM (*n* = 3). Numerical data presented in this figure may be found in S1 Data. Δ-s-tether, Δ-super-tether; cpm, counts per minute; ER, endoplasmic reticulum; PM, plasma membrane; RSR, relative specific radioactivity; SR, specific radioactivity; WT, wild type.

To test directly whether sterol exchange between the ER and PM is affected in Δ-s-tether cells, one of the possibilities suggested by our results on retrograde sterol transport (Fig 3C and 3D), we used a pulse-chase protocol (Fig 4A) to compare the rate at which newly synthesized ergosterol is transported from the ER to the PM [14, 21]. The assay was performed as described previously, using [^3^H]methyl-methionine to pulse-radiolabel ergosterol in the ER [21]. Aliquots of cells taken at different chase time points were homogenized, and the PM was separated from the ER and other internal membranes by sucrose gradient centrifugation. For each time point, the specific radioactivity of [^3^H]ergosterol (SR = scintillation counts [cpm] ÷ absorbance at 280 nm) was determined for the unfractionated cell homogenate and specific fractions after resolving the corresponding lipid extracts by HPLC, and the relative specific radioactivity for each fraction (RSR_fraction_ = SR_fraction_ ÷ SR_cell_) was calculated.

Identical subcellular fractionation profiles were obtained with WT and Δ-s-tether homogenates (Fig 4B), displaying clear separation of the PM from internal membranes, as judged by immunoblotting using antibodies against organelle-specific proteins. The quality of the fractionation was exactly as reported in a previous study, in which a wide spectrum of antibodies was used to confirm the separation of the PM from other membranes [21]. The majority of ergosterol was recovered in the PM fraction from WT cells, as expected, and this was also the case for Δ-s-tether cells, indicating that the subcellular distribution of ergosterol is not affected by the absence of ER-PM contact sites (Fig 4B, bottom panel).

We analyzed fractions 7 (PM) and 2 (ER-enriched; we designated this fraction ER* to indicate that it contains other intracellular membranes [Fig 4B]). The results are shown in Fig 4C. For both WT and Δ-s-tether cells, RSR for ER* was high (>2.0) on completion of the labeling pulse because [^3^H]ergosterol is synthesized in the ER before declining over the chase period to reach a value of 1.0. Conversely, RSR for the PM started at a low level (<0.5; the nonzero value indicates that [^3^H]ergosterol is transported to the PM even as it is being synthesized during the pulse-labeling period) and increased to 1.0 by the end of the chase. The final RSR values of 1.0 for both fractions indicate equilibration of the ergosterol pulse between the ER and PM, as previously reported [14, 21, 55]. Mono-exponential fits of the data indicate that [^3^H]ergosterol is exchanged between the ER and PM with a half time of about 10 min for both WT and Δ-s-tether cells. Thus, the exchange of newly synthesized ergosterol between the ER and PM is normal in Δ-s-tether cells.

We considered the possibility that conventional vesicular transport might deliver sterols to the PM to compensate for the possible failure of non-vesicular modes of transport in Δ-s-tether cells. To test this possibility, we constructed a Δ-s-tether *sec18-1*^ts^ strain that eliminates both exocytosis and ER-PM contact at elevated temperatures. Sec18 is required for exocytosis and most modes of vesicular trafficking [56, 57], and the *sec18-1*^ts^ conditional mutation blocks vesicular transport of secretory proteins and lipids to the PM [56–58]. Whether on its own or in the context of the Δ-s-tether mutations, the *sec18-1*^ts^ allele does not allow cells to grow at 37 °C, and Δ-s-tether *sec18-1* cells do not even grow at 30 °C (S8 Fig). However, the combined growth defects of the Δ-s-tether mutations and *sec18-1* at 30 °C are additive, as would be predicted for unrelated pathways, and do not correspond to a synergistic interaction, as would have been observed between mutations disrupting convergent pathways. After culturing at 23 °C, strains were incubated for 20 min at 37 °C and pulse-labeled with [^3^H]methyl-methionine followed by a 15 min chase. The calculated RSRs of [^3^H]ergosterol in PM fractions showed no significant differences between WT, *sec18-1*, Δ-s-tether, and Δ-s-tether *sec18-1* cells, indicating that ergosterol exchange between the ER and PM is unaffected in all of these strains (Fig 4D). These results indicate that secretory vesicles do not provide a compensatory sterol transport mechanism in Δ-s-tether cells.

We conclude that the exchange of newly synthesized ergosterol between the ER and PM does not require ER-PM contact sites. This result has two clear implications. First, the sterol transfer machinery in yeast is either absent from or not uniquely localized to ER-PM MCSs. This suggests that contact site–localized proteins such as Lam1–Lam4 are not essential for sterol exchange between the PM and ER; these proteins may act redundantly with soluble STPs or play other roles in intracellular sterol homeostasis [30]. Second, yeast cells do not possess soluble STPs capable of lowering the energy barrier for sterol desorption by >10 k_B_T, which would make intracellular sterol transport diffusion-limited rather than desorption-limited [12]. Thus, non-vesicular sterol transport in yeast is likely mediated by cytoplasmic STPs that lower the energy barrier for desorption by a more typical 2–3 k_B_T [12] and that are present in a sufficient number per cell to account for the measured sterol exchange rate [12].

### Does Osh4 account for sterol transport in the absence of ER-PM contact sites?

**Fig 5:**
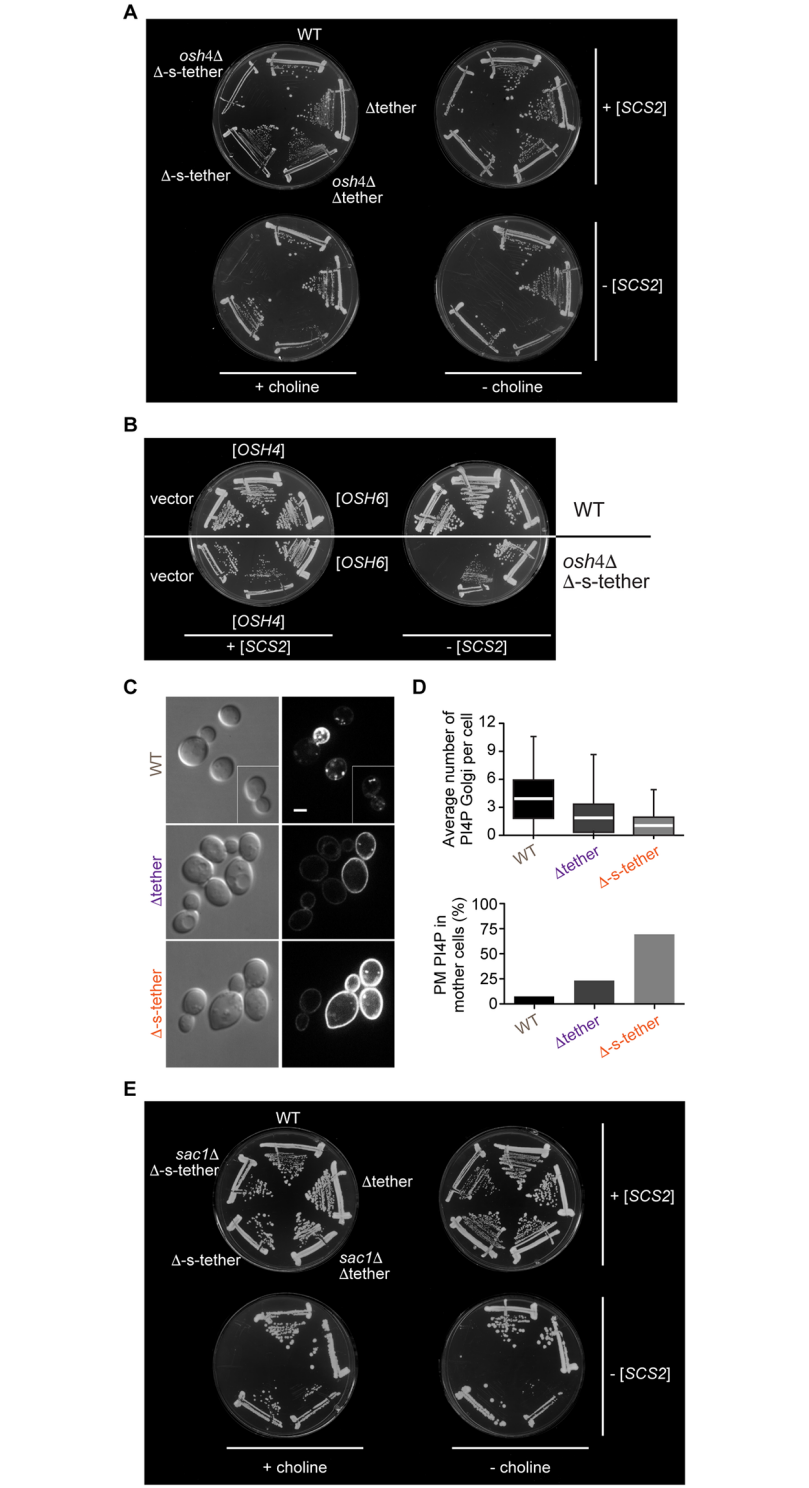
Functional interactions between ER-PM tethers and PI4P regulators. **A.** *OSH4* deletion in Δ-s-tether cells results in synthetic lethality. WT (SEY6210), Δtether (ANDY198), Δ-s-tether (CBY5838), *osh4*Δ Δtether (CBY5940), and *osh4*Δ Δ-s-tether cells (CBY5988) were transformed with an episomal copy of the *SCS2* tether gene (+ [*SCS2*]; pCB1183) and streaked onto selective solid media with and without choline supplementation. The presence of the *SCS2* gene provides an ER-PM tether that confers robust growth, even in the absence of all other tether genes. On growth medium selecting against the *SCS2* plasmid (− [*SCS2*]), *osh4*Δ Δ-s-tether cells were inviable with or without choline. **B.** *OSH6* expression suppresses the synthetic lethality of *osh4*Δ in Δ-s-tether cells. WT and *osh4*Δ Δ-s-tether cells containing an episomal copy of *SCS2* were transformed with either the high-copy vector control (YEplac181), *OSH4* (pCB598), or *OSH6* (pCB1266) and streaked onto solid growth media. On a medium selecting against the *SCS2* plasmid, *OSH4* or *OSH6* expression suppressed *osh4*Δ Δ-s-tether synthetic lethality, whereas vector control did not. **C.** Representative images of WT, Δtether, and Δ-s-tether cells by DIC with corresponding fluorescence microscopy showing the localization of the PI4P sensor GFP-2xPH^*OSH2*^ (pTL511). Scale bar = 2 μm. **D.** Bar graphs quantifying the number of GFP-2xPH^*OSH2*^ fluorescent Golgi spots (lower and upper boundaries of boxes correspond to data quartiles; the white bar indicates the median; lines represent the range of spots/cell) and the percentage of GFP-2xPH^*OSH2*^ fluorescent mothers detected in WT, Δtether, and Δ-s-tether cells. **E.** *SAC1* deletion in Δ-s-tether cells results in a synthetic lethal interaction. WT, Δtether, *sac1*Δ Δtether (CBY6142), Δ-s-tether, and *sac1*Δ Δ-s-tether cells (CBY6146) were transformed with an episomal copy of *SCS2* and streaked onto selective solid media with and without choline supplementation. On a medium that selects against the *SCS2* plasmid, *sac1*Δ Δ-s-tether cells were inviable whether or not choline was added. Numerical data presented in this figure may be found in S1 Data. Δ-s-tether, Δ-super-tether; DIC, differential interference contrast; ER, endoplasmic reticulum; PI4P, phosphatidylinositol-4-phosphate; PM, plasma membrane; WT, wild type.

Osh4 is one subset of Osh proteins capable of binding sterols, and it is present in high levels in yeast at >30,000 copies per cell [59]. Although elimination of Osh4 had no effect on sterol transport, as measured via assays of sterol import [20] or ER-PM sterol exchange (S9 Fig), we tested whether Osh4 might nevertheless provide a compensatory sterol transport mechanism to allow normal ER-PM sterol exchange in Δ-s-tether cells, where the absence of ER-PM contact sites would prevent any putative membrane-bound STPs from reaching their target membrane. Consistent with a possible redundancy between Osh4 and ER-PM contact sites in sterol transport, we discovered that *osh4*Δ Δtether cells grew poorly and *osh4*Δ Δ-s-tether cells were inviable (Fig 5A). In contrast, deletion of the putative sterol transporter encoded by *LAM2* had no impact on Δ-s-tether cells, whether cultured with or without added choline (S10 Fig). This result is consistent with the fact that the Δ-s-tether mutations compromise Lam2 function by eliminating its proximity to the cell cortex (S2 Fig), and therefore no further effect would be anticipated on eliminating expression of the protein itself. Expression of Scs2 from a plasmid rescued *osh4*Δ Δ-s-tether cell lethality, but choline supplementation did not (Fig 5A). This result suggests that an ER-PM tether is required to restore viability to these strains; tether-independent, choline-induced phospholipid synthesis is either irrelevant to *osh4*Δ Δ-s-tether synthetic lethality, or choline supplementation is simply insufficient to overcome the severity of the phospholipid defect.

To test if *osh4*Δ Δ-s-tether synthetic lethality results from defects in intracellular sterol transport, we generated a conditionally viable strain that combines Δ-s-tether mutations with a temperature-sensitive *osh4-1* allele [60]. At 36 °C, *osh4-1 osh4*Δ Δ-s-tether cells do not grow, and so we measured DHE transport from the PM to the ER 1 h after switching to the inactivating temperature. When *OSH4* is inactivated in Δ-s-tether cells in this manner, DHE transfer and esterification were found to be the same as in Δ-s-tether cells (S11 Fig). We conclude that the elimination of *OSH4* has no further impact on sterol transport from the PM to the ER in Δ-s-tether cells.

Each of the seven *OSH* genes can provide the essential requirement for the entire family of *OSH* genes [22]. Even though they are defined as “OSBP homologues,” not all Osh proteins are able to bind sterols, but all likely bind PI4P [61, 62]. Thus, Osh6 binds PI4P and PS in a mutually exclusive fashion but cannot bind sterols [23, 24]. As another way to determine if the *osh4*Δ Δ-s-tether synthetic lethality relates to the sterol- versus PI4P-binding activities of Osh4, we therefore tested if Osh6 could functionally replace Osh4 in this context. As shown in Fig 5B, expression of Osh6 from a multicopy plasmid rescued the growth defect of *osh4*Δ Δ-s-tether cells. Plasmid-based expression of Osh6 was important for growth rescue, as the chromosomally expressed protein, present at fewer than 2,000 copies per cell [59], was not able to support growth. Lipidomics analysis of *osh4*Δ Δ-s-tether cells rescued with multicopy *OSH6* did not reveal an obvious mode of suppression by changes in lipid metabolism (S6C Fig). Compared to Δ-s-tether cells, levels of most sphingolipid precursors and phospholipids (including PS) were unchanged in the *OSH6*-rescued cells or showed minor reductions. The minor reduction in free ergosterol measured in Δ-s-tether cells was restored to WT levels in *OSH6*-rescued *osh4*Δ Δ-s-tether cells, and ergosterol ester levels doubled over WT. These results are consistent with a model in which Osh4 and ER-PM tethers function redundantly and independently, but in an important function revolving around PI4P, with indirect effects on sterol metabolism.

To explore this model further, we used the PI4P marker GFP-PH^Osh2^ to compare the distribution of PI4P in WT and Δ-s-tether cells. It had been previously reported that PI4P was dysregulated in Δtether cells [5] and we anticipated that this phenotype might be exacerbated in Δ-s-tether cells. WT cells showed PI4P concentrated at the PM only in buds and also localized to the Golgi apparatus (Fig 5C and 5D); this distribution was disrupted in Δ-s-tether cells, in which PI4P was evenly distributed throughout the PM in both mother cells and buds (Fig 5C and 5D). The intensity of PI4P staining in the PM of Δ-s-tether mother cells was greater than that seen for Δtether cells (Fig 5C and 5D). To test if the artificial staple could correct the PI4P accumulation/depolarization phenotype, GFP-PH^Osh2^ and the ER-PM staple were both expressed in Δ-s-tether cells (S12 Fig). Although at a gross level, the artificial tether did not restore normal PI4P polarization, PI4P was absent at the immediate cortical sites where the staple interacted with the PM, suggesting a potentially local corrective effect. The addition of choline to Δ-s-tether cells had no impact on GFP-PH^Osh2^ depolarization (100% of Δ-s-tether cells cultured with or without choline had equal GFP-PH^Osh2^ fluorescence in mother and bud PM, compared to 4.7% of WT cells grown with no added choline and 3.6% of WT cells with choline; *n* > 104 cells), indicating that ER-PM MCS regulation of PI4P in the PM is distinct from the role of MCSs in PC metabolism. Taken together, our results suggest that the synthetic lethality of the Δ-s-tether mutations with *osh4*Δ is associated with dysregulation of PI4P homeostasis.

Osh4 and several other Osh proteins have been shown to be upstream regulators of the PI4P phosphatase Sac1, inducing its activity and thereby affecting PI4P levels in several cellular membranes, including the PM [63]. Sac1 is an ER-membrane protein that interacts with most of the ER-PM tethers deleted in the Δ-s-tether strain [5], placing it in a position to act across ER-PM contact sites to dephosphorylate PM-localized PI4P. If Osh4 acts through Sac1 in the same pathway, then *sac1*Δ might also be synthetically lethal in Δ-s-tether cells. This was indeed the case (Fig 5E), consistent with the model that Osh4 and Sac1 function in a PI4P regulatory pathway operating alongside ER-PM tethers. One possibility is that Sac1 itself provides limited tethering, a function that might be induced by the absence of the other tethers in Δ-s-tether cells. However, expressing the soluble enzymatic domain of Sac1 (Sac1^1–522^) without its ER membrane-binding domain suppressed *sac1*Δ Δ-s-tether lethality (S13A Fig). This result indicated that Sac1 does not act as a tether and that Sac1 dependence on MCSs can be partially bypassed if Sac1 is released from the membrane, so that it can access PI4P in the PM. Because Osh4 acts in vitro as a soluble PI4P transport protein [64], it might function in cells to extract and transport PI4P from the PM to Sac1 in the ER. We tested if the requirement for Osh4-mediated PI4P transport could be circumvented if Sac1 was freed from the ER membrane to diffuse to the PM to dephosphorylate PI4P. However, soluble Sac1^1–522^ expressed from a multicopy plasmid did not rescue *osh4*Δ Δ-s-tether lethality, indicating that the requirement for Osh4 cannot be bypassed by liberating Sac1 from the ER (S13B Fig). Thus, in the context of ER-PM MCSs, Osh4 might play an important role in Sac1 regulation, but it clearly has other independent functions as well.

### Altered lipid organization in the PM of Δ-s-tether cells

We have shown that the absence of ER-PM contact sites does not affect sterol exchange between the ER and PM (Fig 4C) and that this lack of effect is not due to compensatory sterol transport by secretory vesicles (Fig 4D) or by the single most abundant sterol-binding protein in yeast, Osh4 (Fig 5B). We can therefore pinpoint the cause of slow transport-coupled esterification of exogenously supplied DHE in Δ-s-tether cells to step 2 in the scheme depicted in Fig 3A, i.e., the exchange of sterol between sterol pools within the PM lipid bilayer. To investigate this point, we tested the growth of Δ-s-tether cells in the presence of three drugs that report on the lipid organization of the PM: nystatin, duramycin, and edelfosine.

**Fig 6:**
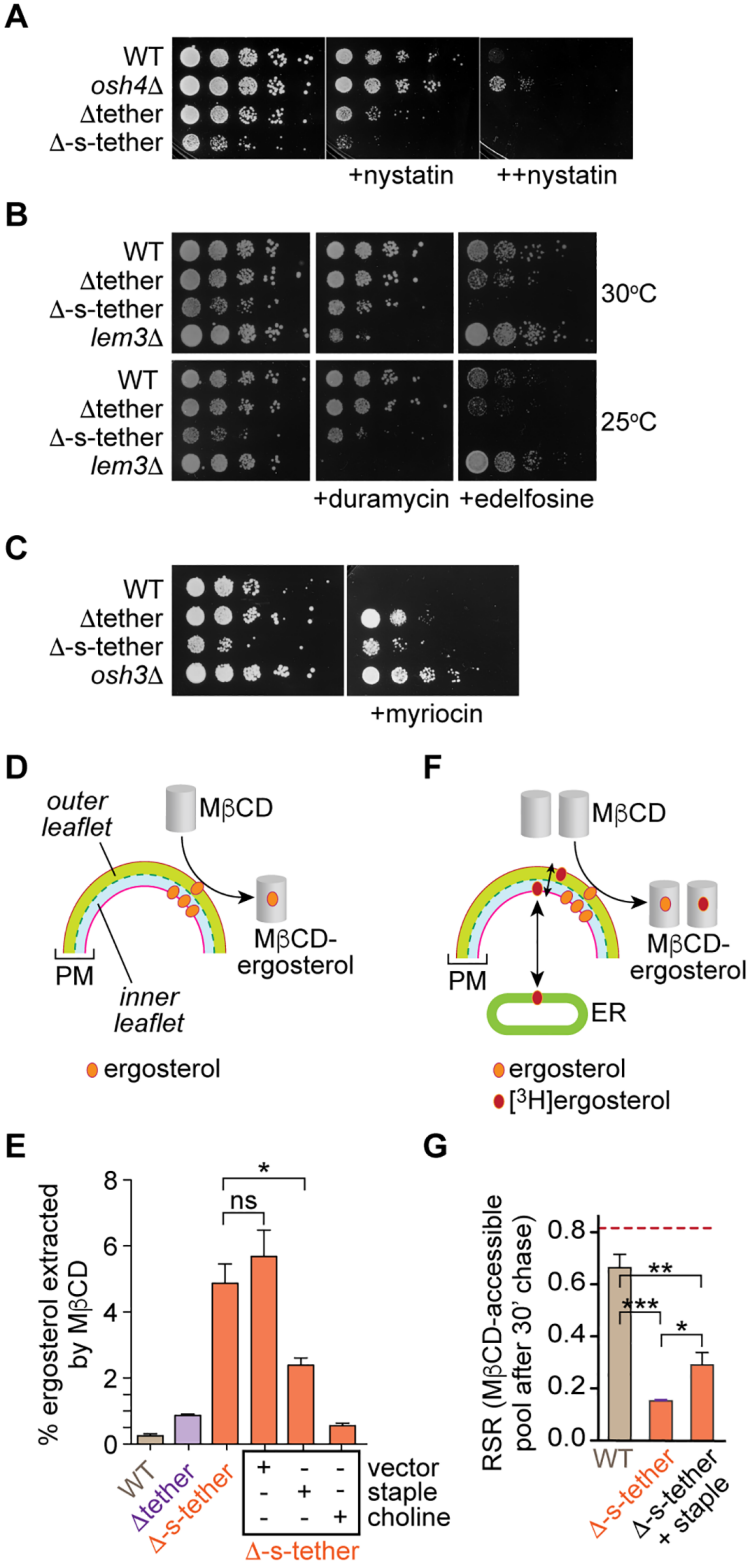
Alterations in ergosterol pools and dynamics at the PM in Δ-s-tether cells. **A.** Sensitivity of Δ-s-tether cells to nystatin. Tenfold serial dilutions of WT (SEY6210), *osh4*Δ (HAB821), Δtether (ANDY198), and Δ-s-tether (CBY5838) cultures spotted onto solid rich medium containing no nystatin, 1.25 μM (+) nystatin, or 2.5 μM (++) nystatin and incubated for 3 d at 30 °C. **B.** Tenfold serial dilutions of WT, Δtether, and Δ-s-tether, *lem3*Δ (CBY5194) cultures were spotted onto solid rich media containing no drug, 5 μM duramycin, or 60 μM edelfosine and incubated for 2 d at 25 °C and 30 °C. The *lem3*Δ strain is known to be duramycin-sensitive and was used as a positive control. **C.** Tenfold serial dilutions of WT, Δtether, Δ-s-tether, and *osh3*Δ (JRY6202) cultures were spotted onto solid rich media containing no drug or 0.5 μg/mL myriocin and incubated for 2 d at 30 °C. The *osh3*Δ strain is known to be myriocin resistant and was used as a positive control. **D.** Assay to measure the proportion of cellular ergosterol that is extracted by MβCD. The PM of a yeast cell is shown, with outer (green) and inner (blue) leaflets delineated. Incubation of cells with MβCD on ice results in extraction of ergosterol from the outer leaflet. The sample is centrifuged to recover MβCD-ergosterol complexes in the supernatant. Ergosterol is extracted from the cell pellet and supernatant with hexane/isopropanol and quantified by HPLC (UV detection). **E.** The MβCD-accessible pool of ergosterol (quantified as in panel D) is about 20-fold greater in Δ-s-tether cells versus WT cells, and partially restored to WT levels in cells expressing the “ER-PM staple.” The statistical significance of the difference between the measurement of WT cells and each of the different Δ-s-tether samples is *p* < 0.0001, and between the Δ-s-tether samples is *p* = 0.0205 (*) and 0.436 (ns). **F.** Assay to measure transport of newly synthesized ergosterol from the ER to the MβCD-accessible pool. Cells are pulse-labeled with [^3^H]methyl-methionine to generate [^3^H]ergosterol in the ER, and chased as described in Fig 3. After a 30 min chase period, energy poisons are added and cells are placed on ice and incubated with MβCD. The ratio of the specific radioactivity of ergosterol in MβCD-ergosterol complexes versus that of the cell homogenate (RSR) provides a measure of transport. **G.** Transport of newly synthesized ergosterol from the ER to the MβCD-accessible pool. The bar chart shows RSR values for the different samples. The dotted line indicates the average RSR (about 0.82, averaged over both WT and Δ-s-tether samples) after 30 min of chase for the PM fraction, as described in Fig 3. The statistical significance was determined by one-way ANOVA (***p = 0.0003, **p = 0.0027, *p = 0.043). Numerical data presented in this figure may be found in S1 Data. Δ-s-tether, Δ-super-tether; ER, endoplasmic reticulum; HPLC, high-performance liquid chromatography; MβCD, methyl-β-cyclodextrin; ns, not significant; PM, plasma membrane; RSR, relative specific radioactivity; UV, ultraviolet; WT, wild type.

Nystatin is an ergosterol-binding polyene antimycotic compound. Nystatin resistance is observed in viable sterol biosynthesis mutants and some mutants, such as *osh4*Δ [51], that disrupt sterol organization within the PM. Conversely, many mutants with altered lipid composition and/or PM organization exhibit nystatin sensitivity [51, 65]. On nystatin-containing medium, Δ-s-tether cells exhibited an exacerbated growth sensitivity compared to Δtether cells, and both strains were more sensitive than WT or nystatin-resistant *osh4*Δ cells (Fig 6A).

Duramycin is a lantibiotic that disrupts cell growth by directly binding PE in the outer leaflet of the PM. As PE is principally located in the cytoplasmic leaflet of the PM, duramycin sensitivity indicates changes in PE bilayer asymmetry, as seen in the phospholipid-flippase mutant *lem3*Δ. Growth of WT, Δtether, and Δ-s-tether cells was not significantly affected by duramycin (Fig 6B), indicating that transbilayer phospholipid asymmetry is unaffected. We next tested edelfosine, a cytotoxic lysophosphatidylcholine analogue whose activity in yeast is modulated by PM phospholipid flippase activity, and by sterol and sphingolipid pathways. A flippase defect confers edelfosine resistance [66], whereas changes in the lipid composition and physical properties of the PM confer edelfosine sensitivity [67]. Δ-s-tether cells displayed acute cytotoxicity to edelfosine compared to WT or even Δtether cells (Fig 6B), consistent with changes in PM properties.

Based on the sensitivity of Δ-s-tether cells to edelfosine (Fig 6B) as well as the significant reductions in their sphingolipid levels revealed by lipidomics analyses (Fig 2G), we considered the possibility that the cells would exhibit a growth phenotype in response to the sphingolipid synthesis inhibitor myriocin (Fig 6C). Indeed, previous work had shown that elimination of the three Tcbs alone causes myriocin sensitivity [35]. Unexpectedly, both Δtether and Δ-s-tether cells were myriocin resistant (Fig 6C). These results suggest that myriocin toxicity in Δ-s-tether cells is mitigated by compensatory alterations either in membrane composition or in the sphingolipid biosynthesis apparatus. Taken together, the results of our drug screening experiments indicate that changes in PC and sphingolipid organization in Δ-s-tether cells might indirectly modulate sterol pools within the PM.

The perturbation in PM lipid organization revealed by drug tests (Fig 6A–6C) was not evident in measurements of the ergosterol “status” of the cell (S14 Fig). Thus, when comparing WT and Δ-s-tether cells, we found no significant difference in the fraction of total cellular ergosterol that was recovered in detergent-insoluble membranes (DIMs) (S14F Fig), a crude readout of the extent to which ergosterol associates with phospholipids and sphingolipids containing saturated acyl chains [14, 21]. Likewise, there were no significant differences in the total ergosterol content of the cells (S14A Fig), the ergosterol/phospholipid ratio (S14B Fig), or the fraction of cellular ergosterol located in the PM (Fig 4B). Because these bulk measurements are unlikely to be responsive to nuanced changes in lipid composition and organization, we chose a more sensitive technique to probe ergosterol organization at the PM.

Methyl-β-cyclodextrin (MβCD) extracts only a very small fraction, <0.5%, of total cellular ergosterol from the outer leaflet of the PM of WT cells under our standard conditions [14, 21], indicative of the unusual physical properties of the yeast PM [14, 21, 68–70]. When PM lipid organization is perturbed, then the amount of MβCD-extractable sterol can increase dramatically, as seen as in *osh*Δ *osh4-1*^*ts*^ cells and sphingolipid-deficient *lcb1-100*^*ts*^ cells at the nonpermissive temperature [14, 21]. We compared the MβCD-extractability of ergosterol in WT cells versus the tether mutants (Fig 6D). As reported previously, the proportion of ergosterol extracted from WT cells by MβCD is about 0.25% of total cellular ergosterol [14, 21]; a similarly low level of extraction (<1%) was obtained with Δtether cells (Fig 6E). However, in Δ-s-tether cells, the MβCD-accessible ergosterol pool in the PM was >5%, about 20-fold greater than for WT cells, consistent with a major change in the PM lipid bilayer that enabled greater extraction of ergosterol. This effect was largely reset by expression of the ER-PM staple and completely restored to WT levels by supplementing the growth medium with choline (Fig 6E). The ability of both the ER-PM staple and choline to restore PM lipid organization, as revealed by MβCD-extractability of ergosterol, parallels their ability to correct the slowdown in retrograde transport of DHE (Fig 3D). Thus, these results are consistent with the idea that the reduced rate of transport-coupled esterification of DHE is due to perturbations of the PM lipid bilayer that delay the access of exogenously supplied DHE to cytoplasmic STPs (Fig 3D, step 2). The ability of choline to provide the same corrective effect as the ER-PM staple without inducing membrane contacts indicates that the role of tethers in this context is to support normal phospholipid and/or sphingolipid homeostasis, and thereby membrane organization.

Although the exchange of ergosterol between the ER and PM as a whole was unchanged in Δ-s-tether cells (Fig 4C and 4D), we investigated if movement of ergosterol within the PM bilayer might be affected. Non-vesicular transport of newly synthesized [^3^H]ergosterol deposits ergosterol molecules in the cytoplasmic leaflet of the PM. At a minimum, these molecules must exchange with the outer leaflet pool of ergosterol before they fully equilibrate with PM ergosterol pools and become accessible to MβCD (Fig 6F). We tested if the exchange of ergosterol within the PM was affected in Δ-s-tether cells by measuring the rate at which newly synthesized ergosterol becomes accessible to MβCD extraction. We used [^3^H]methyl-methionine to pulse-label ergosterol in the ER and then chased the cells for 30 min. The samples were subjected to MβCD extraction and, in parallel, samples were taken for subcellular fractionation to isolate the PM (as in Fig 4B). Both the MβCD extract and the PM fraction were processed with organic solvents to extract ergosterol for HPLC analysis and measurement of RSR. The RSR for the PM fraction after a 30 min chase was about 0.8 for both WT and Δ-s-tether cells (Fig 6G, dashed line), as expected (Fig 4C). However, the RSR for MβCD-extracted ergosterol in WT cells was about 0.65 (Fig 6G, WT), indicating a slight delay in the transport of ergosterol within the PM to the MβCD-accessible pool in the outer leaflet, consistent with our previous report [21]. This delay was considerably greater in Δ-s-tether cells, where the MβCD-extracted ergosterol had an RSR of only about 0.15 after a 30 min chase (Fig 6G, Δ-s-tether). Expression of the ER-PM staple reduced the delay significantly, such that the RSR increased to about 0.3 in Δ-s-tether cells chased for 30 min (Fig 6G, Δ-s-tether + staple). We conclude that (i) the transfer of ergosterol from its site of arrival at the cytoplasmic leaflet of the PM to the outer leaflet pool, from which it can be extracted by MβCD, is slower than the rate at which ergosterol exchanges between the ER and PM as a whole, as reported previously [21], and (ii) the intra-PM movement of ergosterol, from the inner to the outer leaflet, is dramatically slower in Δ-s-tether cells compared with WT cells. Taken together with the fact that the abundance of characteristic PM lipids, e.g., IPC, MIPC, and PS, in Δ-s-tether cells differs significantly from WT cells (Fig 2G), it seems likely that the changes in ergosterol organization in the PM and the rate of exchange between ergosterol pools in the PM are an indirect consequence of changes in PM phospholipid and sphingolipid composition.

### Membrane contacts between the ER and PM proliferate in response to sterol depletion

We have shown that membrane contact between the ER and PM impacts the abundance of PM lipids (sphingolipids, PE, PS [Fig 2G], and PI4P [Fig 5C]), PM lipid organization (Fig 6A, 6B, 6C and 6E), and the intra-PM movement of ergosterol (Fig 6G). In turn, it is known that PM lipids play a role in the establishment of contact sites (Fig 1A); thus, phosphoinositides and PS in the PM provide anchors for ER-localized Tcb1–Tcb3, Ist2, and Scs2 [6, 8, 36–40]. As sterols represent a large fraction of PM lipids and are critical determinants of PM organization [10, 11], we analyzed the potential role of sterols in establishing contact sites between the ER and PM.

**Fig 7:**
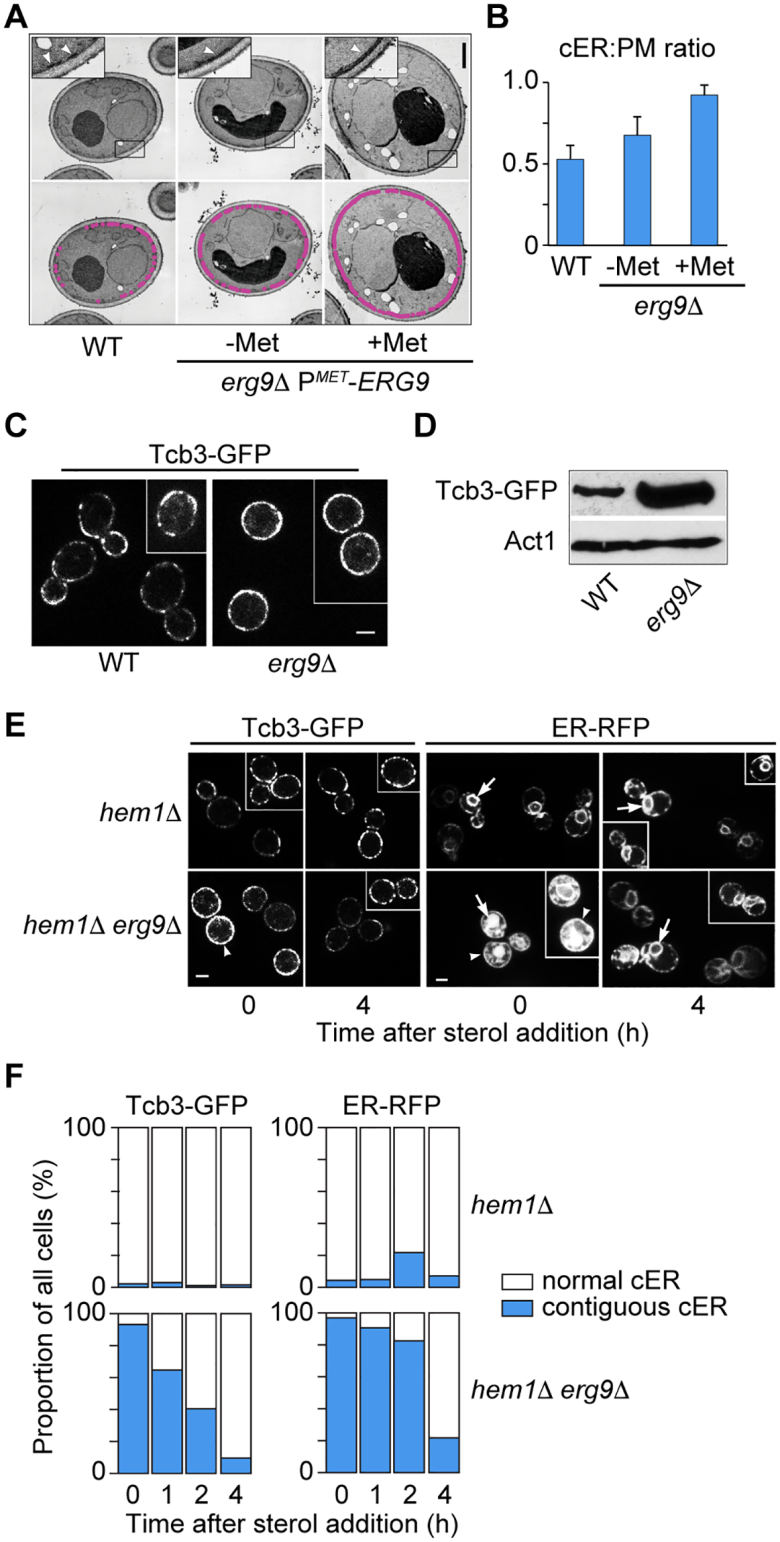
Sterol depletion induces both ER-PM MCS formation and Tcb3 tether expression. **A.** Electron micrographs of WT (CBY858) and *erg9*Δ P^*MET3*^-*ERG9* (CBY745) cells before (− Met) and after (+ Met) methionine repression of P^*MET3*^-*ERG9* synthesis of sterols (methionine was also added to WT). Inserts correspond to magnifications of boxed regions at the cell cortex showing PM-associated ER (arrowheads); cER is highlighted in magenta. Scale bar = 2 μm. **B.** Corresponding to panel **A**, quantification of cER length expressed as a percentage of the total circumference of the PM in each cell section counted (*n* = 25 cells for each strain; error bars show SD; *p* = 7.6 × 10^−25^ for the difference between WT and *erg9*Δ P^*MET3*^-*ERG9* (+ Met)). **C.** WT (CBY5836) and *erg9*Δ (CBY5834) cells with integrated *TCB3-*GFP and P^*MET3*^-*ERG9* constructs in the presence of methionine, which represses *ERG9* expression and sterol synthesis in *erg9*Δ cells. Scale bar = 2 μm. **D.** Corresponding to panel **C**, representative immunoblots probed with anti-GFP and anti-actin antibodies showing Tcb3-GFP levels in WT and sterol-depleted *erg9*Δ P^*MET3*^-*ERG9* cells, as compared to the actin (Act1) control. Relative to WT, Tcb3-GFP levels increased 5.6 ± 1.6 (mean ± SD; *n* = 5)-fold in sterol-depleted *erg9*Δ P^*MET3*^-*ERG9*. **E**. Continuous Tcb3-GFP and ER-RFP fluorescence along the PM (arrowheads) dissipated with the addition of exogenous cholesterol to sterol-depleted *hem1*Δ *erg9*Δ P^*MET3*^-*ERG9* cells (CBY5995 and CBY5842 pCB1024, respectively). Intense ER-RFP nuclear fluorescence (arrows) also diminished after cholesterol addition. The normal discontinuous dashed line of Tcb3-GFP and ER-RFP around the cell cortex was unaffected in sterol-prototrophic *hem1*Δ cells (CBY5993 and CBY5844 pCB1024, respectively); scale bar = 2 μm. **F.** Quantification of contiguous association between cER and the PM after cholesterol addition in *hem1*Δ, *TCB3*-GFP *hem1*Δ cells, and sterol-depleted *hem1*Δ *erg9*Δ P^*MET3*^-*ERG9* cells and *TCB3*-GFP *hem1*Δ *erg9*Δ P^*MET3*^-*ERG9* cells. Following cholesterol addition to sterol-depleted *hem1*Δ *erg9*Δ P^*MET3*^-*ERG9* cells, reductions in cortical Tcb3-GFP localization were detected 1 h after cholesterol addition, with reductions in cER-RFP lagging slightly behind (*n* > 100). Numerical data presented in this figure may be found in S1 Data. cER, cortical ER; ER, endoplasmic reticulum; *ERG9*, squalene synthase; GFP, green fluorescent protein; MCS, membrane contact site; PM, plasma membrane; RFP, red fluorescent protein; Tcb, tricalbin; WT, wild type.

To test the dependence of MCS formation on ergosterol, we depleted yeast cells of sterols and visualized cER-PM association by both transmission electron microscopy and Tcb3-GFP and RFP-ER distribution by fluorescence microscopy. Squalene synthase (Erg9) represents the first sterol-specific enzymatic step in the production of all sterols, and inhibition of Erg9 specifically blocks sterol synthesis without directly affecting other isoprenoids [71]. In *erg9*Δ P^*MET3*^-*ERG9* cells, methionine addition to the growth medium represses Erg9 expression and de novo sterol synthesis stops. To our surprise, electron microscopy showed that sterol depletion in *erg9*Δ P^*MET3*^-*ERG9* cells resulted in a dramatic expansion of cER (Fig 7A), such that the inner face of the PM was nearly completely covered with associated ER membrane (Fig 7B). This finding was confirmed by confocal fluorescence microscopy in live Tcb3-GFP–expressing cells. In sterol-replete WT cells, Tcb3-GFP fluorescence exhibited a characteristic discontinuous stitched pattern around the cortex (Fig 7C) [35]. In about 90% of sterol-depleted *erg9*Δ P^*MET3*^-*ERG9* cells, however, cortical fluorescence was essentially contiguous (Fig 7C). Although sterol-depleted cells accumulate as unbudded cells in the G1-phase of the cell-cycle, G1-arrested *cdc42-101* cells did not induce any change in Tcb3-GFP distribution, indicating that increased ER-PM contact is not due to G1 arrest per se (S15 Fig). These results indicate that ER-PM membrane association is induced when cellular sterol synthesis is blocked.

In addition to its altered distribution along the PM, Tcb3-GFP fluorescence was generally greater in sterol-depleted cells relative to WT, suggesting an induction of Tcb3 protein levels in response to sterol reduction. This point was verified by analyzing cell extracts prepared from both WT and *erg9*Δ P^*MET3*^-*ERG9* sterol-depleted cells expressing Tcb3-GFP and determining relative levels of Tcb3-GFP by SDS-PAGE/immunoblotting using anti-GFP antibodies. When normalized to levels of the actin (Act1) internal control, Tcb3-GFP protein levels were seen to be induced about 6-fold in sterol-depleted cells, compared to similarly treated WT cells (Fig 7D). In genome-wide analyses of gene expression by DNA microarray, sterol depletion had no impact on transcript levels of any of the tether genes; relative to WT cells, methionine repression of de novo sterol synthesis in *erg9*Δ P^*MET3*^-*ERG9* cells showed transcriptional changes between 0.93 and 1.05 ± 0.03 (mean ± SD; independent duplicate trials) for each of the seven tether protein genes. These results indicated that Tcb3 protein levels are posttranscriptionally regulated.

Because of the long half-life of cellular sterols, an extended period is required after *ERG9* repression for complete sterol depletion. To determine how quickly sterol-depleted cells recover their normal distribution of ER-PM association, ER-RFP and Tcb3-GFP redistribution was measured in response to exogenously added cholesterol. Under standard culture conditions, yeast does not import sterols from the medium as discussed above (Fig 3), but the deletion of *HEM1* permits cholesterol uptake [72]. A *hem1*Δ *erg9*Δ P^*MET3*^-*ERG9* strain could grow after sterol depletion, but only when exogenous cholesterol (or δ-aminolevulinic acid [δ-ALA], the product of the Hem1 enzyme) was supplemented to the growth medium (S16 Fig). In *hem1*Δ cells, ER-RFP and Tcb3-GFP distributions were the same with or without cholesterol supplementation (Fig 7E). In sterol-depleted *hem1*Δ *erg9*Δ P^*MET3*^-*ERG9* cells, return to the normal discontinuous stitched fluorescence of cortical Tcb3-GFP commenced 1 h after cholesterol addition, and the characteristic WT pattern was observed after about 4 h (Fig 7E and 7F). In these cells, recovery of normal ER-RFP morphology lagged behind the restoration of the normal Tcb3-GFP distribution (Fig 7F), consistent with the idea that tethering complexes dictate changes in cER association. These results indicate that tethering between the ER and PM responds to cellular sterol pools.

## Discussion

MCSs are widely hypothesized to facilitate non-vesicular lipid exchange. We tested this hypothesis in the context of sterol exchange between the ER and PM in yeast by creating Δ-s-tether cells that lack ER-PM membrane contacts. We now report that these contact sites are not required for ER-PM sterol exchange but rather function as regulators of PM lipid homeostasis, controlling the organization and dynamics of sterols within the PM and acting redundantly with the OSBP homologue Osh4 in an essential pathway related to PI4P homeostasis. We also report our unexpected discovery that in the absence of sterol biosynthesis, ER-PM contact sites proliferate, such that the entirety of the PM is associated with ER because of increased expression of tethers. These results invite a revision of current thinking about the role of contact sites as hubs for lipid transport and reveal a reciprocal relationship between the formation and function of contact sites on the one hand and lipid homeostasis on the other.

### Ice2 is an ER-PM tether

To create Δ-s-tether cells, we eliminated *ICE2* in the previously described Δtether strain. The role of Ice2 in distributing ER along the PM between mother and daughter cells during mitosis is well established [33, 44], hinting that it may play a direct role in tethering ER to the PM. Ice2 is a polytopic ER membrane protein with a single prominent cytoplasmic loop that has been implicated in associating the ER with lipid droplets during the stationary phase of growth, and potentially channeling DAG to the phospholipid biosynthetic machinery in the ER as cells resume growth [45]. In analogy to its proposed tethering role in stationary phase cells, we speculate that Ice2 may play a role in bridging the ER and PM in rapidly dividing cells. Indeed, fluorescence microscopy reveals that Ice2 is located at the cell cortex in Δtether cells (S3 Fig). As a potential tether protein, the cytoplasmic loop of Ice2 may interact directly in *trans* with the cytosolic face of the PM or, similar to the Scs2 tether [5, 73], Ice2 might form a bridge across the ER-PM interface via an interaction with another protein. If the latter scenario is correct, then the mechanism of tethering by both Ice2 and Scs2 would differ from that of the autonomous membrane attachments conferred by Ist2 and the E-Syt homologues Tcb1–Tcb3 (Fig 1A). Nevertheless, eliminating Ice2 in the context of Δtether cells results in quantifiable reductions in ER-PM association beyond those previously reported for Δtether cells (Fig 1E and S1 Fig), leading to clear functional outcomes. For example, synthetic lethality of Δ-s-tether with *osh4*Δ or *sac1*Δ was not manifested in the progenitor Δtether strain and only occurred with the additional deletion of *ICE2*. Likewise, slowing of transport-coupled esterification of DHE (Fig 3C) and increased extractability of ergosterol by MβCD (Fig 6E) were observed only after deletion of *ICE2* in Δtether cells. Taken together, these findings show that Ice2 is an important contributor to ER-PM tethering and associated functions.

### Normal ER-PM sterol exchange the absence of contact sites

We found that bidirectional sterol exchange between the ER and PM occurs at the same rate in Δ-s-tether and WT cells, indicating that ER-PM contact sites do not contribute quantitatively to the mechanism of sterol movement between these two membranes. If any elements of the sterol transport machinery are localized to ER-PM MCSs, then their function must be subsumed by other sterol transport mechanisms in Δ-s-tether cells. However, we show that in and of themselves, neither secretory vesicles (Fig 4D) nor the cytoplasmic sterol-binding protein Osh4 (Fig 5A and 5B) or the ER-anchored Lam2 protein (S10 Fig) provide this putative compensatory mechanism in Δ-s-tether cells. Our results also make it clear that yeast cells do not possess STPs with the ability to lower the energy barrier for sterol desorption to the point at which transport becomes a diffusion-limited rather than desorption-limited process [12]. Thus, non-vesicular sterol transport in yeast is likely mediated by unremarkable cytoplasmic STPs (i.e., STPs that are able to lower the energy barrier for sterol desorption by only 2–3 k_B_T) present in a sufficient number per cell to account for the measured sterol exchange rate [12]. The identification of these STPs is a focus of future work.

### Altered sterol organization and dynamics in the PM of Δ-s-tether cells

Even though ER-PM sterol exchange was unaffected by the lack of ER-PM contact sites, trafficking of exogenously supplied DHE to the ER was unexpectedly slow in Δ-s-tether cells compared with WT cells (Fig 3C). Careful analysis of the various mechanistic steps of the transport process (Fig 3A) revealed that the slowdown could be linked to a dramatic lowering of the rate at which sterols equilibrate between the inner and outer leaflet of the PM in Δ-s-tether cells (Fig 6F and 6G). While this slowdown should not affect the equilibration of DHE with PM sterol pools during the extended hypoxic loading period used for this assay (Fig 3A, steps 1 and 2), it would affect the rate at which newly synthesized ergosterol displaces DHE from the PM during the aerobic chase, thereby resulting in a slower rate of transport-coupled DHE esterification. We previously reported that the appearance of newly synthesized ergosterol in the MβCD-extractable ergosterol pool in the outer leaflet of the PM lags behind its arrival at the PM in WT cells (Fig 6D and 6E), suggesting that equilibration of sterol across the yeast PM is considerably slower than that seen for cholesterol flip-flop in synthetic, liquid crystalline membranes and in red blood cells [74–76]. This may be a consequence of the unusual properties of the yeast PM, exemplified by the slow lateral diffusion of both lipids and proteins [69, 70] and the organization of PM proteins into a mosaic of domains [77]. In the case of Δ-s-tether cells, the rate of transbilayer sterol equilibration was about 5-fold slower than for WT cells (Fig 6G), and this was reflected in changes in PM bilayer organization, as manifested in the nystatin and edelfosine sensitivity of these cells and the greater accessibility of sterols to MβCD extraction (Fig 6A, 6B and 6E). Quantification of cellular lipids revealed reductions in PE, PS, and the sphingolipids IPC and MIPC (Fig 2G). As these lipids generally reflect PM composition [78], we propose that relative changes in lipid levels are the underlying cause of the disturbance in the PM bilayer, resulting in a change in ergosterol dynamics.

### PM phospholipid dysregulation in Δ-s-tether cells

How do ER-PM contact sites affect PM lipid organization and intra-PM ergosterol dynamics? Because the growth defect of Δ-s-tether cells was rescued by choline supplementation (Fig 2E), which increases flux through phospholipid biosynthetic pathways [79] without inducing contact site formation (Fig 2F), the primary defect in cells lacking ER-PM contact sites appears to involve phospholipid regulation. Remarkably, both choline and the artificial staple corrected most defects inherent to Δ-s-tether cells (i.e., slow growth [Fig 2B], slow transport-coupled DHE esterification [Fig 3D], high MβCD-extractability of ergosterol [Fig 6E], and slow rate of ergosterol exchange between PM pools [Fig 6G]). These results indicate that the function of the endogenous tethers, even those such as Tcb1, Tcb2, and Tcb3, with lipid-transporting SMP domains [48] might be largely structural in this context, i.e., the tethers provide a means of mechanical attachment of the ER to the PM, thereby enabling ER-localized proteins, such as the PC-synthesizing phospholipid methyltransferase Opi3, to act in *trans*.

An exception to this general conclusion is that neither choline nor the artificial staple was able to re-establish normal PI4P polarization in Δ-s-tether cells (Fig 5C and 5D and S12 Fig). PI4P dephosphorylation at the PM is proposed to be due to the ER-localized Sac1 phosphatase acting in *trans* at ER-PM contact sites [80], although the protein clearly also acts in *cis* [81]. The inability of the artificial staple to facilitate PM access for Sac1 might stem from either (i) an insufficient number or improper positioning of membrane contacts established by the staple and/or (ii) the inability of the staple to provide a specific requirement for Sac1 activation, which is otherwise provided by endogenous tethers (almost all tethers were identified by virtue of specific interactions with Sac1 [5], whereas the artificial tether would lack this ability). Our data suggest that PM PI4P is reduced in the immediate vicinity of contact sites generated by the artificial staple (S12B Fig), indicating that Sac1 might access PI4P locally at those points. In contrast, endogenous tethers and their ancillary factors might further expand cER-associated regions of the PM that are accessible to Sac1.

In yeast, all phospholipids are synthesized from phosphatidic acid (PA) via the CDP-DAG pathway; PE and PC are also synthesized by the Kennedy pathway using DAG and salvaged or exogenously supplied ethanolamine and choline [79, 82] (S17 Fig). PA levels and the DAG:PA ratio are critical in determining the amount of CDP-DAG available for phospholipid synthesis (S17 Fig). The mole percentage of PA in Δ-s-tether cells is 20% lower than that in WT cells, and the DAG:PA ratio is 2-fold greater (Fig 2G), indicating dysregulation of phospholipid synthesis in the absence of ER-PM contact sites. Consistent with this, levels of PS and PI-derived sphingolipids are much lower in Δ-s-tether than in WT cells (Fig 2G), although PI levels are unaffected (see below). As previously shown [31], *ice2*Δ *scs2*Δ cells have a diminished ability to convert PS to PC via Opi3-mediated phospholipid methylation at ER-PM contact sites, necessitating choline supplementation for normal growth. In Δ-s-tether cells, choline supplementation would not only bypass the need for Opi3 but also compensate for the lower overall rate of phospholipid synthesis resulting from decreased PA levels by generating PC via the Kennedy pathway for membrane growth. Lipidomic analysis of Δ-s-tether cells cultured with choline did in fact show restoration of PC to levels comparable to WT (S6A Fig). These results are also consistent with the choline-reversible synthetic growth defects observed when either *OPI3* or *CHO2* is deleted in Δ-s-tether cells (S4A Fig). Thus, ER-PM contact sites may function as regulatory interfaces that coordinate the CDP-DAG and Kennedy pathways to balance convergent mechanisms for phospholipid synthesis.

Δ-s-tether cells have a normal mole percentage of PI and increased PI4P, yet their content of inositol sphingolipids is about 60% lower than in WT cells (Fig 2G), indicating dysregulation of phosphoinositide homeostasis because of loss of ER-PM contacts. Total cellular PI is generated predominantly by the CDP-DAG pathway and Sac1-mediated dephosphorylation of PI4P (itself generated from PI) in separate cellular locations, with both routes providing the biosynthetic precursor for complex inositol sphingolipids (S17 Fig). Brice and colleagues [83] reported that disruption of *SAC1* alone reduces PI levels dramatically and IPC and MIPC levels by >70%. The Δ-s-tether mutations would reduce the Sac1-mediated route for PI production by distancing the enzyme from its substrate in the PM (Fig 5C). However, *sac1*Δ and Δ-s-tether mutations are lethal when combined, likely due to limiting PI levels, indicating that Δ-s-tether mutations must also inhibit PI production from the CDP-DAG pathway. Normal PI levels in Δ-s-tether cells may therefore be a consequence of preserving this lipid at the expense of inositol sphingolipid production. It should be noted that Sac1 is also a component of the SPOTS (SPT, Orm1/2, Tsc3, and Sac1) complex that regulates early steps in sphingolipid synthesis [84, 85]. However, levels of ceramide, an early precursor in sphingolipid synthesis, were essentially normal in Δ-s-tether cells (Fig 2G). It therefore seems unlikely that the SPOTS complex plays a direct role in ER-PM contact site regulation of inositol sphingolipids.

### Intersection of Osh4, Sac1, and ER-PM MCSs in PI4P homeostasis

While investigating whether normal ER-PM sterol transport in Δ-s-tether cells could be due to compensatory activity of soluble or membrane-bound STPs, we discovered that the deletion of *OSH4* was synthetically lethal with Δ-s-tether mutations (Fig 5A). Lethality was not due to a sterol-related process because Osh6, which does not bind sterols, could rescue *osh4*Δ Δ-s-tether growth defects (Fig 5B). Consistent with previous proposals that Osh proteins represent important regulators of PI4P [15, 80], the deletion of *SAC1* in Δ-s-tether cells also resulted in synthetic lethality (Fig 5E). Based on these findings, we propose that Osh4 (and Sac1) functions in a parallel pathway alongside ER-PM contact sites for PI4P regulation. However, expression of the soluble enzymatic domain of Sac1 did not suppress *osh4*Δ Δ-s-tether lethality (S13 Fig), suggesting that the downstream regulation of Sac1 is not the only role Osh4 plays at ER-PM MCSs. The availability of Δ-s-tether cells now allows further interrogation of the mechanism by which ER-PM MCSs function as interfaces for regulating PI4P signaling and phospholipid metabolism.

Our results are consistent with the hypothesis that ER-PM contact sites constitute a regulatory nexus to balance sterol and phospholipid concentrations to maintain PM structure. In the absence of contact sites, the ratio of sterols to specific phospholipids and sphingolipids is uncoupled, which negatively impacts PM organization, intra-PM sterol dynamics, and PI4P levels. When sterols become limiting, posttranscriptional induction of Tcb3 increases the extent of ER-PM association, potentially facilitating compensatory changes to phospholipid synthesis to re-establish bilayer stability. Although the mechanisms that control Tcb3 levels in sterol-replete or sterol-depleted cells are not known, it is interesting to speculate that the induction of a tether protein may represent a new homeostatic mechanism for regulating PM composition and structure.

## Materials and methods

### Strains, plasmids, and microbial and genetic techniques

Yeast strains and plasmids are listed in the Supporting information (S1 and S2 Tables, respectively). Unless otherwise stated, yeast cultures were grown in synthetic complete or YPD rich media at 30 °C. All temperature-sensitive alleles were cultured at permissive growth temperatures (30 °C unless otherwise stated) and shifted to the restrictive temperature of 37 °C, as specified. DNA cloning and bacterial and yeast transformations were carried out using standard techniques [86, 87].

For the choline and ethanolamine supplementation growth assays, yeast strains were cultured in synthetic minimal media for 48 h, then streaked onto solid synthetic complete media containing 1 mM choline chloride or 1 mM ethanolamine (Sigma-Aldrich Chemicals, St. Louis, MO). Growth in response to inositol supplementation was tested on synthetic minimal media containing 75 μM myo-inositol (Sigma-Aldrich Chemicals). For the nystatin sensitivity plate assay, 10-fold serial dilutions of yeast cultures were spotted onto solid synthetic media containing 2.5 μM nystatin (Sigma-Aldrich Chemicals). Cell growth was also tested on solid rich media containing 60 μM edelfosine (Cayman Chemical, Ann Arbor, MI), 5 μM duramycin (Sigma-Aldrich Chemicals), or 0.5 μg/mL myriocin (Sigma-Aldrich Chemicals). To select against *URA3*-marked plasmids (e.g., pCB1183), yeast cultures were grown on rich growth media and then streaked onto a synthetic solid growth medium containing 1 g/L 5-fluoroorotic acid (Gold Biotechnology, St. Louis, MO). For assays of cholesterol uptake by cells lacking *HEM1*, yeast cultures were spotted onto a solid synthetic medium lacking methionine, containing 25 μg/mL cholesterol in 1% (vol/vol) Tween 80–ethanol (1:1 [vol/vol]) and 50 μg/mL δ-ALA (Sigma-Aldrich Chemicals). For sterol depletion assays, *erg9Δ* P^*MET3*^-*ERG9* cells were grown at 30 °C for 10 h in synthetic media lacking methionine and grown to mid-log phase before adding 100 mg/L methionine.

DNA cloning and bacterial and yeast transformations were carried out using standard techniques [86, 87]. The functional artificial tether fusion plasmids pCB1185 and pCB1188 were derived from pRS416-P^*YSP1*^-eGFP-Myc-HMH-RitC, a kind gift from Tim Levine (UCL Institute of Ophthalmology). To construct pCB1185, coding sequences from pRS416-P^*YSP1*^-eGFP-Myc-HMH-RitC were amplified using CACTCGAGTTATGGAGCAAAAGCTCATTTCTGAAGAG and CAGGTACCCTATACTGAATCCTTTTTCTTACGGAAT primers, and the product was digested with *Xho*I/*Kpn*I for subcloning in frame with GFP under the control of an *ACT1* promoter in a YCplac111 vector. To construct pCB1188, coding sequences from pRS416-P^*YSP1*^-eGFP-Myc-HMH-RitC were amplified using the primers: CATCCGGACTTATGGAGCAAAAGCTCATTTCTGAAGAG and CATCTAGACTATACTGAATCCTTTTTCTTACGGAATGG. The amplified product was digested with *Kpn*I/*Xba*I and subcloned in frame with coding sequences for mCherry under the control of an *ACT1* promoter in a YCplac111 vector.

All genomic manipulations were performed by integration of PCR amplified product as previously described [88]. All natMX4 and hphMX4 deletions were generated by homologous recombination into the yeast genome of targeted P4339 and pAG32 amplified products; transformants were selected for on YPD media containing 100 mg/L nourseothricin (Gold Biotechnology) and 400 mg/L hygromycin B (Toku-E, Bellingham, WA), respectively. For growth of *hem1*Δ::natMX4 cells, selective growth media contained 50 μg/mL δ-ALA. The *sec18-1*:*URA3* temperature-sensitive allele strains were isolated at 23 °C after genomic recombination of the *sec18-1*:*URA3* gene cassette amplified from CBY2853 genomic DNA. All transformants were confirmed by genomic PCR or genetic complementation assays.

### Transmission electron microscopy

Yeast cells were grown to mid-log phase and prepared (fixation, dehydration, infiltration/embedding) as previously described [89]. Minor changes were made to the infiltration schedule as follows: ethanol:resin (2:1) was incubated overnight while ethanol:resin (1:1) was incubated for 5 h. For calculations of cER abundance in electron micrographs, the ratio between PM and the length of PM associated with cER were determined using ImageJ (www.imagej.nih.gov/ij/index.html); cER was assigned as previously described [90].

### FIB-SEM

The resin block was microtomed to expose a clean face and then attached to a metal SEM stub with carbon tape. The sides were coated with silver paint to increase conductivity. The block was then sputter coated with a thin coat of Au/Pd and inserted into the FIB-SEM. Areas of interest were identified by viewing with the electron beam at 25 keV. Serial block-face imaging: the area of interested was coated with 1 μm-thick Pt in the microscope using the Pt deposition needle. The sample was tilted to 52 degrees and an approximately 30 μm trench was cut in front of the area. The FEI Slice and View G2 program was used for data collection, with the following parameters: imaging at 2 keV; 50 pA current; 30 μs dwell time; horizontal field width, 17.74 μm; tilt angle, 60 degrees (cross-sectional viewing angle, −30 degrees); working distance, 2.5 mm; and TLD detector set to −245 V suction tube voltage for backscatter imaging. The slice thickness was set at 20 nm, so the final voxel size was 8.66 nm in X, 10 nm in Y, and 20 nm in Z. Image processing: raw images were aligned using the xfalign tool in IMOD [91]. Images were then corrected for density gradients using ImageJ software [92]. The aligned and corrected tiff images were imported into Amira for density-guided segmentation (FEI Software, Hillsboro, OR) and display.

### Fluorescence microscopy and live-cell imaging

Confocal fluorescence microscopy was performed as previously described [93]. For all experiments, yeast cells were grown to mid-log phase before visualization. GFP-Staple (pCB1185) and mCherry-Staple (pCB1188) fusion proteins were imaged using 150 and 750 ms exposures, respectively. RFP-ER (pCB1024 and pCB1277) and RFP-RAS2 (pCB1204) were imaged using a 750 ms exposure on the confocal. GFP-2xPH^*OSH2*^ (pTL511) was imaged by confocal microscopy using a 250 ms exposure. Tcb3p-GFP and Ice2p-GFP were imaged by confocal microscopy using 350 ms and 1.5 s exposures, respectively.

Widefield fluorescence microscopy was performed as previously described [94]. GFP-2xPH^*OSH2*^ (pTL511) imaged by widefield epifluorescence was acquired using a 200 ms exposure, 30% arc lamp intensity, and analog gain set to full. Bleed-through between fluorescence channels was undetectable under the conditions used for image acquisition. All contrast enhancement was kept constant for each series of images.

### Sterol transport assays

Transport of exogenously supplied DHE from the PM to the ER was determined as previously described [21, 51]. Briefly, DHE was loaded into the PM of cells under hypoxic conditions, and its transport to the ER upon subsequent aerobic chase was monitored by fluorescence microscopy and quantified by lipid extraction and HPLC to determine the extent of conversion to DHE-ester. To quantify the initial fluorescence of DHE-loaded cells, individual cells were outlined using ImageJ; then, the corresponding area and integrated density were measured to determine the corrected total cell fluorescence (CTCF), as previously described [95]; CTCF = (integrated density − (area of selected cell × mean background fluorescence)). At least 40 cells were counted (from 4 individual fields) for each strain. The ACAT activity of the cells was assayed with microsomes using a modification of a published procedure [96], as described [21, 51].

Biosynthetic sterol transport was measured using a pulse-chase labeling procedure as previously described [21, 51]. Briefly, cells were labeled with [^3^H]methyl-methionine for 4 min to generate a pulse of [^3^H]ergosterol in the ER and subsequently chased with unlabeled methionine. Transport was assessed after subcellular fractionation to isolate the PM or after MβCD extraction to sample ergosterol in the outer leaflet of the PM. Ergosterol in cells, subcellular fractions, and MβCD extracts were solubilized using organic solvents and quantified by HPLC.

### Lipidomics

For lipidomics analysis, cells were grown to about OD_600_ 0.8 and lipids were extracted with chloroform:methanol (2:1). Yeast lipid extracts were prepared using a standard chloroform-methanol mixture, spiked with appropriate internal standards, and analyzed using a 6490 Triple Quadrupole LC/MS system (Agilent Technologies, Santa Clara, CA) [97]. Glycerophospholipids and sphingolipids were separated with normal-phase HPLC as described before [97], with a few changes. An Agilent Zorbax Rx-Sil column (inner diameter 2.1 × 100 mm) was used under the following conditions: mobile phase A (chloroform:methanol:1 M ammonium hydroxide, 89.9:10:0.1, v/v) and mobile phase B (chloroform:methanol:water:ammonium hydroxide, 55:39.9:5:0.1, v/v); 95% A for 2 min, linear gradient to 30% A over 18 min and held for 3 min, and linear gradient to 95% A over 2 min and held for 6 min. Sterols and glycerolipids were separated with reverse-phase HPLC using an isocratic mobile phase as before [97], except with an Agilent Zorbax Eclipse XDB-C18 column (4.6 × 100 mm).

Quantification of lipid species was accomplished using multiple reaction monitoring (MRM) transitions [97, 98] in conjunction with the referencing of appropriate internal standards: PA 17:0/14:1, PC 17:0/20:4, PE 17:0/14:1, PG 17:0/20:4, PI 17:0/20:4, PS 17:0/14:1, LPC 17:0, LPE 14:0, Cer d18:1/17:0, D7-cholesterol, cholesteryl ester (CE) 17:0, 4ME 16:0 diether DG, D5-TG 16:0/18:0/16:0 (Avanti Polar Lipids, Alabaster, AL). Quality and batch controls [99] were included to assess instrument stability and reproducibility and allow for correction of drift and other systematic noise, e.g., biases correlated with analysis order and/or sample preparation. Values are represented as mole fraction with respect to total lipid (mole percentage) [97]. All lipid species and subclasses were analyzed with one-way ANOVA followed by a post hoc Bonferroni test.

### Immunoblots

For analysis of Tcb3 protein expression, 10 OD_600_ units of Tcb3p-GFP–expressing cells post sterol depletion were prepared as described by Ohashi and colleagues [100]. Pellets were resuspended in SDS sample buffer and boiled for 5 min before SDS-PAGE. Protein transfer to nitrocellulose membranes and immunoblot conditions were as previously described [101]. To detect Tcb3p-GFP, immunoblots were incubated with a 1:1,000 anti-GFP antibody (ThermoFisher Scientific Inc., Waltham, MA) followed with 1:10,000 anti-rabbit-HRP secondary antibody (Bio-Rad Laboratories, Mississauga, ON). Actin was detected using 1:1,000 anti-actin antibody (Cedarlane, Burlington, ON) followed with 1:10,000 anti-mouse-HRP secondary antibody (ThermoFisher Scientific Inc.).

For analysis of Ysp2 protein expression, 10 OD_600_ units of GFP-Ysp2–expressing cells were prepared and proteins extracted as above. To detect GFP-Ysp2, immunoblots were incubated with 1:2,000 anti-GFP antibody (Sigma-Aldrich Chemicals) followed by 1:10,000 anti-rabbit-HRP secondary antibody (Promega, Madison, WI). GAPDH was detected using 1:10,000 anti-GAPDH antibody (ThermoFisher Scientific Inc.) followed with 1:10,000 anti-mouse-HRP secondary antibody (Promega).

### Estimate of the random chance of finding ER at the cell cortex in cells lacking tethers

We derive a rough estimate of the chance of finding cER at the cell cortex in yeast cells that lack the ability to tether the ER to the PM as follows. Assuming that a yeast cell has a volume of 65 μm^3^ (radius = 2.5 μm) [102, 103], we estimate the volume of a cortical shell defined by the reported distance (30 nm) at which the ER is retained at the PM by tethers as 1.9 μm^3^. As about 45% of the PM is associated with ER in WT cells (Fig 1E and references [5, 32]), the volume of the cortical shell that is occupied by ER is 0.9 μm^3^. If this amount of ER were to become untethered, then it could be found anywhere in the total volume of the cell. Approximately 65% of the total cell volume is available for this purpose, i.e., 42 μm^3^, as the rest is occupied by the nucleus and organelles [104]. Thus, the random chance of finding the dispersed complement of cER anywhere in the cell, including the cell cortex, is 0.9/42 = 0.02, or about 2%.

## Supporting information

S1 Table Yeast strains. (DOCX)

S2 Table Plasmids. (DOCX)

S1 Data Numerical data presented in Supporting figures. (XLSX)

S1 Fig Histograms of the cER/PM ratio for WT, Δtether, and Δ-s-tether cells. WT (SEY6210), Δtether (ANDY198), and Δ-s-tether (CBY5838) cells were processed for electron microscopy and the cER/PM ratio was measured for each cell as described in Fig 1D and 1E. Frequency distributions were obtained using 10 bins in each case. Panel **A** compares all three strains, whereas panel **B** expands the region 0 < cER/PM < 0.15 to highlight significant differences between the tether mutants. Note that the cumulative distributions shown in Fig 1E are derived from the raw, unbinned data, with *N* = 41 for both mutants. The Kolmogorov-Smirnov test gave Dmax = 0.41 and *p* = 0.001. The Wilcoxon Rank Sum test gave *U* = 1,681 and a two-tailed *p*-value of 0.0006. Δ-s-tether, Δ-super-tether; cER, cortical ER; PM, plasma membrane; WT, wild type.
(TIF)

S2 Fig Localization of Lam2 in WT and tether mutants. **A.** GFP-Lam2 was expressed from a plasmid (pGFP-Lam2) in WT (SEY6210), Δtether (ANDY198), and Δ-s-tether (CBY5838) cells. The cells were stained with CellMask Orange for 5 min to mark the PM before fluorescence microscopy. Arrowhead: fluorescence in the cell interior. Scale bar = 5 μm. **B.** Frequency distribution indicating the number of fluorescent cortical dots in WT, Δtether, and Δ-s-tether cells expressing GFP-Lam2. More than 90 cells of each strain were scored and the distribution was plotted using a bin size of 2. The average numbers of cortical dots per cell were 16, 14, and 4 for WT, Δtether, and Δ-s-tether, respectively. **C.** Anti-GFP immunoblots indicated that GFP-Lam2 levels were unaffected in Δtether and Δ-s-tether cells as compared to wild type, using anti-GAPDH as the loading control. Δ-s-tether, Δ-super-tether; GAPDH, glyceraldehyde 3-phosphate dehydrogenase; GFP, green fluorescent protein; PM, plasma membrane; WT, wild type.
(TIF)

S3 Fig Ice2 localizes to the ER and is found at cortical ER sites. **A.** Ice2-GFP in Δtether cells (CBY6220) was observed at nuclear ER and in ER tubules that extend to the cell periphery, as demarcated by RFP-Ras2 (pCB1204). In this strain, the remaining cortical attachments of ER to the PM contain Ice2-GFP (arrowheads). **B.** Serial optical sections focused at the top, middle, and bottom of Δtether cells expressing Ice2-GFP and the ER marker RFP-ER (pCB1024). The merged images of Ice2-GFP and RFP-ER fluorescence superimposed onto corresponding DIC whole cell images indicate complete colocalization. At optical sections near the cell cortex, Ice2-GFP was present as discrete spots (arrows) in equal or greater fluorescence relative to RFP-ER, consistent with ER-PM MCSs. DIC, differential interference contrast; ER, endoplasmic reticulum; GFP, green fluorescent protein; MCS, membrane contact site; RFP, red fluorescent protein.
(TIF)

S4 Fig Opi3 and Cho2 affect choline-sensitive growth defects of Δ-s-tether cells. **A.** Deletion of *CHO2* or *OPI3* in Δ-s-tether cells results in synthetic growth defects when the cells are cultured without addition of choline to the growth medium. WT (SEY6210), Δ-s-tether (CBY5838), *cho2*Δ Δ-s-tether (CBY6267), and *opi3*Δ Δ-s-tether cells (CBY6271) were streaked onto selective solid media, with or without 1 mM choline, and incubated for 2 d at 30 °C. **B.** Increased expression of Opi3 suppresses choline-sensitive Δ-s-tether growth defects. WT and Δ-s-tether cells transformed with either the vector control (pRS416) or a plasmid expressing *OPI3* from a constitutively active promoter (pOPI3) were streaked onto solid growth media supplemented with or without 1 mM choline, as indicated, and incubated for 2 d at 25 °C. Δ-s-tether, Δ-super-tether; WT, wild type.
(TIF)

S5 Fig Ethanolamine and inositol supplementation do not suppress Δ-s-tether growth defects. WT (SEY6210), Δtether (ANDY198), and Δ-s-tether (CBY5838) cells were streaked onto solid growth media supplemented with 1 mM ethanolamine (A) or 75 μM inositol (B), as indicated, and incubated at 30 °C for 2 or 3 d, respectively. Δ-s-tether, Δ-super-tether; WT, wild type.
(TIF)

S6 Fig Lipidomics analyses of Δ-s-tether cells. **A.** Comparison of the lipid composition of Δ-s-tether cells grown in the absence or presence of 1 mM choline. **B.** Comparison of the lipid composition of Δ-s-tether cells and Δ-s-tether cells expressing an artificial tether (“staple”). The cells were grown in synthetic medium without choline. **C.** Comparison of the lipid composition of Δ-s-tether cells and *osh4*Δ Δ-s-tether expressing Osh6. In all panels, lipid compositions are presented as a normalized mole percentage relative to WT (blue dotted line set to 1.0). The data represent the mean ± SEM derived from the analysis of five independent samples. Δ-s-tether, Δ-super-tether; Cer, ceramide; DG, diacylglycerol; EE, ergosteryl ester; Erg, ergosterol; IPC, inositol-phosphoceramide; LPC, lyso PC; LPE, lyso PE; LPI, lyso PI; MAG, monoacylglycerol; MIPC, mannosylinositol phosphoceramide; mmPE, dimethyl PE; mPE, monomethyl PE; Osh, OSBP homologue; PA, phosphatidic acid; PC, phosphatidylcholine; PCe, ether phosphatidylcholine; PE, phosphatidylethanolamine; PG, phosphatidylglycerol; PI, phosphatidylinositol; PS, phosphatidylserine; TG, triacylglycerol; WT, wild type.
(TIF)

S7 Fig Retrograde transport of exogenously supplied DHE in Δ-s-tether cells supplemented with choline. **A.** Representative images of choline-grown WT and Δ-s-tether cells obtained immediately after DHE loading (chase time = 0 h) and 2 h after incubation under aerobic conditions. The punctae seen in the 2 h chase images correspond to LDs. **B.** DHE esters were quantified at different times during the aerobic chase period by analyzing hexane/isopropanol extracts of the cells by HPLC equipped with an in-line UV detector. The data are represented as percentage of DHE ester recovered (= DHE ester/(DHE + DHE ester)). **C.** Incorporation of DHE into the PM of choline-grown cells, quantified using fluorescence images acquired immediately after the hypoxic incubation period, as detailed in Fig 3E. Fifty cells were analyzed. The box and whiskers plot shows the mean of the measurements, with whiskers ranging from the minimum to the maximum value measured. **D.** Microsomes from choline-grown WT and Δ-s-tether cells were assayed for their ability to esterify [^3^H]cholesterol on the addition of oleoyl-CoA, as described in Fig 3F. The bar chart shows the mean ± SEM (*n* = 3) of ACAT activity as the rate of production of CE per mg microsomal protein per minute. **E.** The amount of ergosterol in choline-grown WT and Δ-s-tether cells (nmol per OD_600_ of cell suspension) was measured by lipid extraction and HPLC at the start and end of the aerobic chase period. Each data point represents a triplicate measurement (the error bars are contained within the symbol used for plotting). Δ-s-tether, Δ-super-tether; ACAT, acetyl-CoA acetyltransferase; CoA, coenzyme A; CE, cholesteryl ester; DHE, dehydroergosterol; HPLC, high-performance liquid chromatography; LD, lipid droplet; OD, optical density; PM, plasma membrane; UV, ultraviolet; WT, wild type.
(TIF)

S8 Fig Growth of strains carrying the *sec18-1*^*ts*^ allele. Tenfold serial dilutions of WT (SEY6210), *sec18-1*^*ts*^ (CBY2859), Δtether (ANDY198), Δ-s-tether (CBY5988), and Δ-s-tether *sec18-1*^*ts*^ (CBY5851) cultures were spotted on synthetic complete medium and incubated at the indicated temperatures for 3 d. Δ-s-tether, Δ-super-tether; WT, wild type.
(TIF)

S9 Fig Normal transport of newly synthesized ergosterol to the PM in *osh4*Δ cells. Transport of newly synthesized ergosterol to the PM in WT (SEY6210) and *osh4*Δ (HAB821) cells were measured by pulse-chase radiolabeling as described in Fig 3, using MβCD extraction rather than PM isolation to quantify transport. A pulse of [^3^H]ergosterol was generated in the ER by labeling cells for 4 min with [^3^H]methyl-methionine. Samples were chased for the indicated times. At each chase point, an aliquot of cells was removed, dosed with energy poisons, placed on ice, and incubated with MβCD. Following incubation, the sample was centrifuged and the MβCD-containing supernatant was removed from the cell pellet. Ergosterol was recovered from MβCD-ergosterol complexes as well as from the cell pellet by extraction with hexane/isopropanol, and its specific radioactivity was determined by HPLC (UV detection). The ratio of the specific radioactivity of ergosterol in MβCD-ergosterol complexes versus the cell homogenate RSR provides a measure of transport. Data points represent the mean ± SEM of three independent experiments, each of which comprised duplicate measurements at the indicated time points. ER, endoplasmic reticulum; HPLC, high-performance liquid chromatography; MβCD, methyl-β-cyclodextrin; PM, plasma membrane; RSR, relative specific radioactivity; UV, ultraviolet; WT, wild type.
(TIF)

S10 Fig *LAM2* deletion does not impact growth of Δtether or Δ-s-tether. WT (SEY6210), Δtether (ANDY198), Δ-s-tether (CBY5838), *lam2*Δ Δtether (CBY6150), and *lam2*Δ Δ-s-tether cells (CBY6150) were streaked onto selective solid media with and without 1 mM choline and incubated for 2 d at 30 °C. Δ-s-tether, Δ-super-tether; WT, wild type.
(TIF)

S11 Fig Elimination of Osh4 function in the Δ-s-tether strain does not exacerbate the slow rate of esterification of exogenously supplied DHE. WT (SEY6210), Δ-s-tether (CBY5838), and *osh4-1 osh4*Δ Δ-s-tether (CBY6031) were inoculated from a saturated overnight culture into fresh media (complete synthetic media for WT and Δ-s-tether and the same medium without leucine for *osh4-1 osh4*Δ Δ-s-tether) supplemented with 20 μg/mL DHE and 0.5% Tween:ethanol. The cells were incubated under hypoxic conditions for 36 h at 30 °C before being transferred to 37 °C for 1 h (continuing in hypoxic conditions) and then chased aerobically for the indicated time points at 37 °C. DHE esterification was measured as described in Fig 3. Δ-s-tether, Δ-super-tether; DHE, dehydroergosterol; WT, wild type.
(TIF)

S12 Fig Altered PI4P distribution in Δ-s-tether cells is not corrected by expression of the artificial ER-PM staple. **A.** Wild-type (SEY6210) cells expressing an mCherry-tagged version of the artificial RFP-staple (RFP-staple; pCB1188) and cotransformed with the PI4P sensor GFP-2xPH^*OSH2*^ (pTL511), as shown by DIC and fluorescence confocal microscopy. Scale bar = 5 μm. **B.** Fluorescent images of Δ-s-tether cells (CBY5838) co-expressing GFP-2xPH^*OSH2*^ and the artificial RFP-staple. The boxed region represents an enlarged region shown in the inset, where gaps in the uniform PM PI4P fluorescence coincide with the presence of the artificial RFP-staple. Scale bar = 5 μm. **C.** Quantification of mother cell GFP-2xPH^*OSH2*^ fluorescence at the PM observed as a percentage of all wild-type and Δ-s-tether cells (*n* > 100 cells). Δ-s-tether, Δ-super-tether; DIC, differential interference contrast; ER, endoplasmic reticulum; PI4P, phosphatidylinositol-4-phosphate; PM, plasma membrane; RFP, red fluorescent protein.
(TIF)

S13 Fig The membrane-detached enzymatic SAC1^1–522^ domain suppresses the synthetic lethality of *sac1*Δ Δ-s-tether but not *osh*Δ Δ-s-tether cells. (A) WT (SEY6210) and *sac1*Δ Δ-s-tether (CBY6146) cells expressing an episomal copy of SCS2 (pSCS2) were transformed with the vector control (YCplac111) or plasmids expressing *SAC1* (pRS415 SAC1), *SAC1*^*1–522*^ (pRS415 *SAC1*^*1–522*^), or *SAC1*^*1–522*^ expressed from a high-copy plasmid (pRS425 *SAC1*^*1–522*^). Cells were streaked onto solid growth media containing 5′-FOA (to select against strains that cannot growth without SCS2), supplemented with and without 1 mM choline, for 3 d at 30 °C; *SAC1* and high-copy *SAC1*^*1–522*^ suppressed *sac1*Δ Δ-s-tether synthetic lethality, regardless of choline addition. (B) WT and *osh4*Δ Δ-s-tether (CBY5988) cells containing an episomal copy of *SCS2* were transformed with the vector control or plasmids expressing *SAC1*, *SAC1*^*1–522*^, or high-copy *SAC1*^*1–522*^. Cells were streaked onto solid 5′-FOA–containing media with and without 1 mM choline and incubated for 3 d at 30 °C. In the absence of SCS2, neither *SAC1* nor *SAC1*^*1–522*^ expression suppressed *osh4*Δ Δ-s-tether synthetic lethality. Δ-s-tether, Δ-super-tether; WT, wild type.
(TIF)

S14 Fig Analysis of bulk lipids in WT, Δtether, and Δ-s-tether cells. **A–C.** Quantification of ergosterol, ergosterol:phospholipid molar ratio, and ergosteryl ester in WT (SEY6210), Δtether (ANDY198), and Δ-s-tether (CBY5838) cells. Lipids were extracted and quantified as described in Materials and methods. **D.** Quantification of the number of lipid droplets per cell (*n* > 100 cells counted for each strain). **E.** The SR of ergosterol after labeling cells for 4 min with [^3^H]methyl-methionine was determined by HPLC, as described in Fig 3, and normalized to that of WT cells (set arbitrarily to 1.0). **F.** DIMs were prepared by incubating cells with ice-cold Triton X-100. The proportion of ergosterol in DIMs versus whole cells was quantified by solvent extraction, followed by HPLC analysis. Δ-s-tether, Δ-super-tether; DIM, detergent-insoluble membrane; HPLC, high-performance liquid chromatography; SR, specific radioactivity; WT, wild type.
(TIF)

S15 Fig ER-PM MCSs do not increase in unbudded arrested cells. **A.** Discontinuous cortical Tcb3-GFP distribution was observed in WT (CBY5942) and *cdc42-101*^ts^ (CBY5944) cells incubated 1 h at 37 °C, or at 30 °C. Scale bar = 2 μm. **B.** Percentage of cells with normal discontinuous Tcb3-GFP distribution versus continuous cortical localization along the PM in WT and *cdc42-101*^ts^ cells. Even after G1-arrest for 5 h at 37 °C, Tcb3-GFP in *cdc42-101*^ts^ cells was indistinguishable from WT. ER, endoplasmic reticulum; GFP, green fluorescent protein; MCS, membrane contact site; PM, plasma membrane; Tcb, tricalbin; WT, wild type.
(TIF)

S16 Fig Rescue of sterol-depleted *hem1*Δ *erg9*Δ P^*MET*^-*ERG9* cells with exogenous cholesterol. Tenfold serial dilutions of WT (with an integrated P^*MET*^-*ERG9* construct; CBY918), *erg9*Δ P^*MET*^-*ERG9* (CBY745), and *hem1*Δ *erg9*Δ P^*MET*^-*ERG9* (CBY5844) cultures on synthetic solid medium with (+Met) or without (−Met) methionine, containing (as shown) cholesterol, +δ-ALA, or neither. In the absence of δ-ALA supplementation, all *hem1*Δ strains require methionine for growth, and cholesterol uptake cannot occur under aerobic conditions without the *hem1*Δ mutation. In the presence of methionine, which represses P^*MET*^-*ERG9* expression and sterol synthesis, *hem1*Δ *erg9*Δ P^*MET*^-*ERG9* cells grow (albeit slowly) with 25 μg/mL cholesterol supplementation. δ-ALA, δ-aminolevulinic acid; WT, wild type.
(TIF)

S17 Fig Phospholipid and sphingolipid biosynthetic pathways in yeast. See text for details.
(TIF)

## Abbreviations

δ-ALA: δ-aminolevulinic acid
Δ-s-tether: Δ-super-tether
ABC: ATP-binding cassette
ACAT: acetyl-CoA acetyltransferase
ADP: adenosine diphosphate
CDP-DAG: cytidine diphosphate DAG
CE: cholesteryl ester
cER: cortical ER
CPY*: carboxypeptidase Y*
cpm: counts per minute
CTCF: corrected total cell fluorescence
C2: protein kinase C conserved region 2
DAG: diacylglycerol
DHE: dehydroergosterol
DIC: differential interference contrast
DIM: detergent-insoluble membrane
ER: endoplasmic reticulum
ERAD: ER-associated degradation
Erg9: squalene synthase
E-Syt: extended synaptotagmin
FFAT: two phenylalanines in an acidic tract
FIB-SEM: focused ion beam-scanning electron microscope
GFP: green fluorescent protein
HPLC: high-performance liquid chromatography
IPC: inositol-phosphoceramide
Lam: lipid transfer proteins anchored at MCSs
MβCD: methyl-β-cyclodextrin
MCS: membrane contact site
MIPC: mannosylinositol phosphoceramide
mmPE: dimethyl PE
mPE: monomethyl PE
MRM: multiple reaction monitoring
MSP: major sperm protein
OSBP: oxysterol-binding protein
Osh: OSBP homologue
PA: phosphatidic acid
PC: phosphatidylcholine
PCe: ether phosphatidylcholine
PE: phosphatidylethanolamine
PG: phosphatidylglycerol
PH: Pleckstrin homology
PI: phosphatidylinositol
PIP: phosphatidylinositol phosphate
PI4P: phosphatidylinositol-4-phosphate
PM: plasma membrane
PS: phosphatidylserine
RFP: red fluorescent protein
RitC: C-terminal polybasic region from mammalian Rit1
RSR: relative specific radioactivity
SMP: synaptotagmin-like mitochondrial-lipid-binding protein
SPOTS complex: SPT-Orm1/2-Tsc3-Sac1 complex
SR: specific radioactivity
StAR: steroidogenic acute regulatory
StART: steroidogenic acute regulatory protein–related lipid transfer
STP: sterol transport protein
Tcb: tricalbin
TG: triacylglycerol
UV: ultraviolet
VAMP: vesicle-associated membrane protein
VAP: vesicle-associated membrane protein–associated protein
WT: wild type
