# Supplementary material for: Endoplasmic reticulum-plasma membrane contact sites integrate sterol and phospholipid regulation
Source: PLoS Biol. 2018 May 21;16(5):e2003864. doi: 10.1371/journal.pbio.2003864 (PMC5983861; doi:10.1371/journal.pbio.2003864)
Supplement: S1 Table — (DOCX) [file pbio.2003864.s001.docx]

**S1 Table. Yeast strains.**

Unless otherwise referenced, all strains were created as part of this study.

| Strain | Genotype | Reference |
| --- | --- | --- |
| ANDY198 | *MAT*a *leu2*‐*3,112 ura3*‐*52 his3*Δ*200 trp1*Δ*901 lys2*‐*801 suc2*Δ*9* *ist2*Δ::hisMX6 *scs2*Δ::*TRP1 scs22*Δ::hisMX6 *tcb1*Δ::kanMX6 *tcb2*Δ::kanMX6 *tcb3*Δ::hisMX6 | [[1](#_ENREF_1)] |
| BY4741 | *MAT*a *leu2*Δ0 *ura3*Δ0 *his3*Δ*0 met15*Δ*0* |  |
| CBY745 | *MAT*α *leu2*∆0 *ura3*∆0 *lys2*∆0 *erg9*∆::kanMX4 *HIS3*::P*^MET3^*-*ERG9* | [[2](#_ENREF_2)] |
| CBY858 | *MAT*α *leu2*∆0 *ura3*∆0 *lys2*∆0 *his3*Δ::kanMX4 | [[2](#_ENREF_2)] |
| CBY918 | *MAT*α *leu2*∆0 *ura3*∆0 *lys2*∆0 *ERG9* *HIS3*::P*^MET3^*-*ERG9* |  |
| CBY2859 | SEY6210 *sec18-1*:*URA3* |  |
| CBY5194 | BY4741 *lem3*∆::kanMX4 |  |
| CBY5804 | SEY6210 *ice2*∆::natMX4 |  |
| CBY5834 | CBY745 *TCB3-*GFP:*URA3* |  |
| CBY5836 | CBY918 *TCB3-*GFP:*URA3* |  |
| CBY5838 | ANDY198 *ice2*∆::natMX4 |  |
| CBY5842 | CBY745 *hem1*∆::natMX4 |  |
| CBY5844 | CBY918 *hem1*∆::natMX4 |  |
| CBY5851 | CBY5838 *sec18-1*:*URA3* |  |
| CBY5940 | ANDY198 *osh4*∆::*URA3* |  |
| CBY5942 | *MAT*a *leu2-3,112 ura3‐52 his3*Δ*200 lys2‐801 CDC42*:*LEU2 TCB3-*GFP:*URA3* |  |
| CBY5944 | *MAT*a *leu2-3,112 ura3‐52 his3*Δ*200 lys2‐801 cdc42-101*:*LEU2 TCB3-*GFP:*URA3* |  |
| CBY5980 | ANDY198 *osh4*∆::hphMX4 pCB1183 |  |
| CBY5988 | ANDY198 *osh4*∆::hphMX4 *ice2*∆::natMX4 pCB1183 |  |
| CBY5993 | CBY5844 *TCB3-*GFP*:URA3* |  |
| CBY5995 | CBY5842 *TCB3*-GFP*:URA3* |  |
| CBY6031 | ANDY198 *osh4*∆::hphMX4 *ice2*∆::natMX4 pCB1157 |  |
| CBY6140 | ANDY198 *osh4*∆::hphMX4 *ice2*∆::natMX4 pCB1266 |  |
| CBY6142 | ANDY198 *sac1*∆::hphMX4 |  |
| CBY6146 | ANDY198 *sac1*∆::hphMX4 *ice2*∆::natMX4 pCB1183 |  |
| CBY6150 | ANDY198 *lam2*∆::hphMX4 |  |
| CBY6153 | ANDY198 *lam2*∆::hphMX4 *ice2*∆::natMX4 |  |
| CBY6220 | ANDY198 *ICE2-GFP*:*URA3* |  |
| CBY6267 | ANDY198 *cho2*∆::hphMX4 *ice2*∆::natMX4 |  |
| CBY6271 | ANDY198 *opi3*∆::hphMX4 *ice2*∆::natMX4 |  |
| HAB821 | SEY6210 *osh4*∆*::HIS3* | [[3](#_ENREF_3)] |
| JRY6202 | SEY6210 *osh3*∆::*LYS2* | [[4](#_ENREF_4)] |
| SEY6210 | *MAT*α *leu2*-*3,112 ura3*-*52 his3*Δ*200 trp1*Δ*901 lys2*-*801 suc2*Δ*9* | [[5](#_ENREF_5)] |

**References**

1. Manford AG, Stefan CJ, Yuan HL, Macgurn JA, Emr SD. ER-to-plasma membrane tethering proteins regulate cell signaling and ER morphology. Dev Cell. 2012;23(6):1129-40. Epub 2012/12/15. doi: 10.1016/j.devcel.2012.11.004

S1534-5807(12)00524-2 [pii]. PubMed PMID: 23237950.

2. Beh CT, Rine J. A role for yeast oxysterol-binding protein homologs in endocytosis and in the maintenance of intracellular sterol-lipid distribution. J Cell Sci. 2004;117(Pt 14):2983-96. Epub 2004/06/03. doi: 10.1242/jcs.01157

jcs.01157 [pii]. PubMed PMID: 15173322.

3. Jiang B, Brown JL, Sheraton J, Fortin N, Bussey H. A new family of yeast genes implicated in ergosterol synthesis is related to the human oxysterol binding protein. Yeast. 1994;10(3):341-53. doi: 10.1002/yea.320100307. PubMed PMID: 8017104.

4. Beh CT, Cool L, Phillips J, Rine J. Overlapping functions of the yeast oxysterol-binding protein homologues. Genetics. 2001;157(3):1117-40. Epub 2001/03/10. PubMed PMID: 11238399.

5. Robinson JS, Klionsky DJ, Banta LM, Emr SD. Protein sorting in Saccharomyces cerevisiae: isolation of mutants defective in the delivery and processing of multiple vacuolar hydrolases. Mol Cell Biol. 1988;8(11):4936-48. PubMed PMID: 3062374; PubMed Central PMCID: PMCPMC365587.
