# Supplementary material for: Endoplasmic reticulum-plasma membrane contact sites integrate sterol and phospholipid regulation
Source: PLoS Biol. 2018 May 21;16(5):e2003864. doi: 10.1371/journal.pbio.2003864 (PMC5983861; doi:10.1371/journal.pbio.2003864)
Supplement: S2 Table — (DOCX) [file pbio.2003864.s002.docx]

## **S2 Table. Plasmids**

Unless otherwise referenced, all plasmids were created as part of this study.

| **Plasmid** | **Description** | **Source/Reference** |
| --- | --- | --- |
| p4339 | pCRII-TOPO natMX4 | C. Boone (University of Toronto) |
| pAG32 | pFA6 hphMX4 | [1] |
| pCB598 | YEplac181 *OSH4* |  |
| pCB1024 (RFP-ER) | pRS416 P*^PHO5^*-RFP*-SCS2^220-244^* | [2] |
| pCB1157 | YCplac111 *osh4-1* |  |
| pCB1185 | YCplac111 P*^ACT1^*-GFP-Myc-HMH-RitC |  |
| pCB1188 | YCplac111 P*^ACT1^*-mCherry-Myc-HMH-RitC |  |
| pCB1204 | YCplac111 RFP-*RAS2* |  |
| pCB1266 | YEplac181 *OSH6* |  |
| pCB1277 | YCplac111 P*^PHO5^*-RFP*-SCS2^220-244^* |  |
| pGFP-Lam2 | pRS416 GFP-*LAM2* |  |
| pOPI3 | pRS416 P*^PHO5^*-Myc-*OPI3­* | [3] |
| pRS416 | *URA3 CEN* | [4] |
| pRS415 *SAC1* | pRS415 *SAC1* | [5] |
| pRS415 *SAC1^1-522^* | pRS415 P*^CPS^ -*SAC1^1-522^ | [5] |
| pRS425 *SAC1^1-522^* | pRS425 P*^CPS^ -*SAC1^1-522^ | [5] |
| pSCS2 | pRS416 P*^PHO5^*-Myc-*SCS2* | [3] |
| pTL511 | pRS416 P^CPY^*-*GFP-2xPH*^OSH2^* | [6] |
| YCplac111 | *LEU2 CEN* | [7]] |
| YEplac181 | *LEU2 2μ* | [7] |

**References**

1. Goldstein AL, McCusker JH. Three new dominant drug resistance cassettes for gene disruption in Saccharomyces cerevisiae. Yeast. 1999;15(14):1541-53. doi: 10.1002/(SICI)1097-0061(199910)15:14<1541::AID-YEA476>3.0.CO;2-K. PubMed PMID: 10514571.

2. Loewen CJ, Young BP, Tavassoli S, Levine TP. Inheritance of cortical ER in yeast is required for normal septin organization. J Cell Biol. 2007;179(3):467-83. Epub 2007/11/07. doi: jcb.200708205 [pii]

10.1083/jcb.200708205. PubMed PMID: 17984322; PubMed Central PMCID: PMC2064793.

3. Tavassoli S, Chao JT, Young BP, Cox RC, Prinz WA, de Kroon AI, et al. Plasma membrane--endoplasmic reticulum contact sites regulate phosphatidylcholine synthesis. EMBO Rep. 2013;14(5):434-40. Epub 2013/03/23. doi: 10.1038/embor.2013.36

embor201336 [pii]. PubMed PMID: 23519169; PubMed Central PMCID: PMC3642376.

4. Sikorski RS, Hieter P. A system of shuttle vectors and yeast host strains designed for efficient manipulation of DNA in Saccharomyces cerevisiae. Genetics. 1989;122(1):19-27. Epub 1989/05/01. PubMed PMID: 2659436; PubMed Central PMCID: PMC1203683.

5. Foti M, Audhya A, Emr SD. Sac1 lipid phosphatase and Stt4 phosphatidylinositol 4-kinase regulate a pool of phosphatidylinositol 4-phosphate that functions in the control of the actin cytoskeleton and vacuole morphology. Mol Biol Cell. 2001;12(8):2396-411. PubMed PMID: 11514624; PubMed Central PMCID: PMCPMC58602.

6. Roy A, Levine TP. Multiple pools of phosphatidylinositol 4-phosphate detected using the pleckstrin homology domain of Osh2p. J Biol Chem. 2004;279(43):44683-9. doi: 10.1074/jbc.M401583200. PubMed PMID: 15271978.

7. Gietz RD, Sugino A. New yeast-Escherichia coli shuttle vectors constructed with in vitro mutagenized yeast genes lacking six-base pair restriction sites. Gene. 1988;74(2):527-34. PubMed PMID: 3073106.
