# Supplementary figures and images for: Endoplasmic reticulum-plasma membrane contact sites integrate sterol and phospholipid regulation
Source: PLoS Biol. 2018 May 21;16(5):e2003864. doi: 10.1371/journal.pbio.2003864 (PMC5983861; doi:10.1371/journal.pbio.2003864)

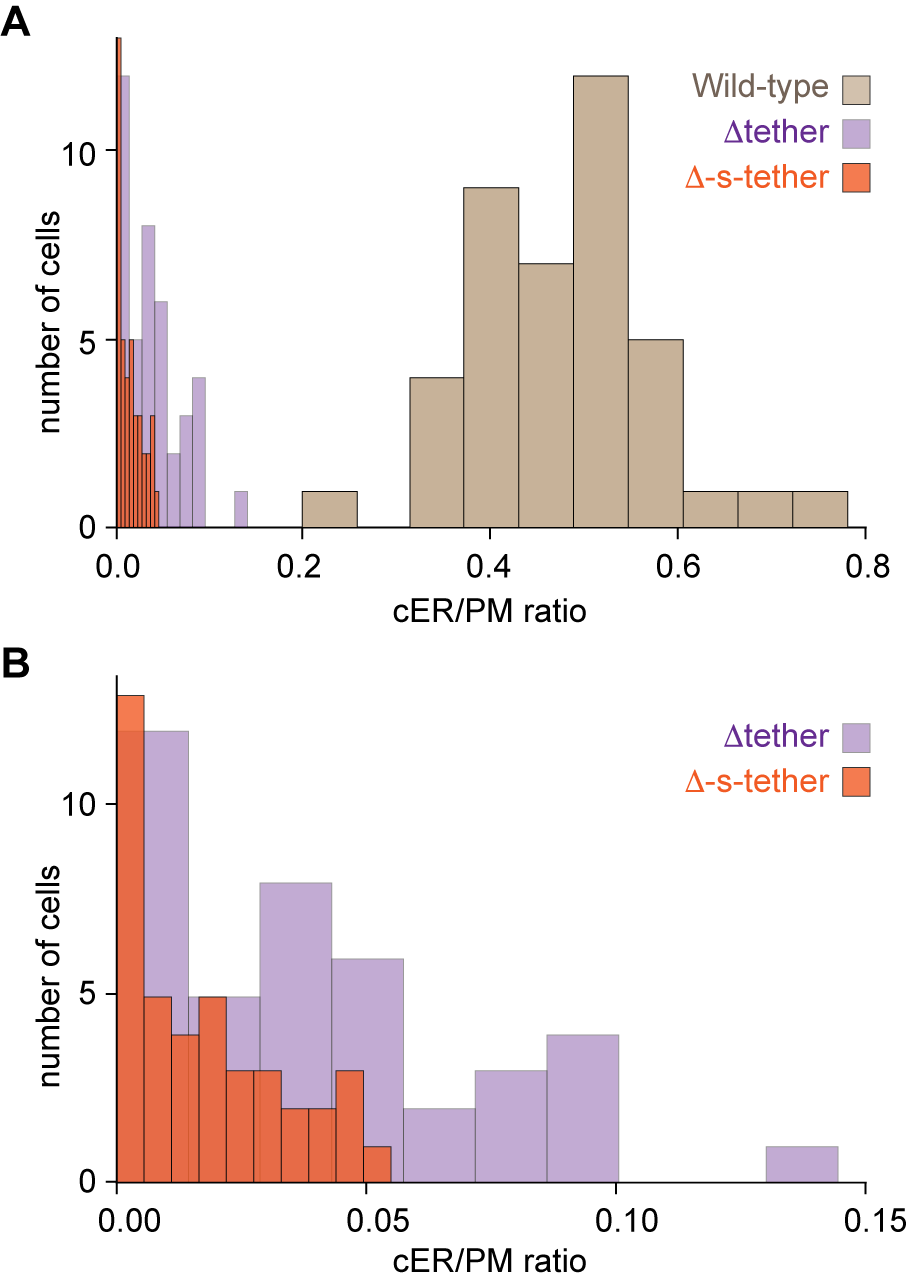

Supplement: S1 Fig — WT (SEY6210), Δtether (ANDY198), and Δ-s-tether (CBY5838) cells were processed for electron microscopy and the cER/PM ratio was measured for each cell as described in Fig 1D and 1E. Frequency distributions were obtained using 10 bins in each case. Panel A compares all three strains, whereas panel B expands the region 0 < cER/PM < 0.15 to highlight significant differences between the tether mutants. Note that the cumulative distributions shown in Fig 1E are derived from the raw, unbinned data, with N = 41 for both mutants. The Kolmogorov-Smirnov test gave Dmax = 0.41 and p = 0.001. The Wilcoxon Rank Sum test gave U = 1,681 and a two-tailed p-value of 0.0006. Δ-s-tether, Δ-super-tether; cER, cortical ER; PM, plasma membrane; WT, wild type. (TIF) [file pbio.2003864.s004.tif]

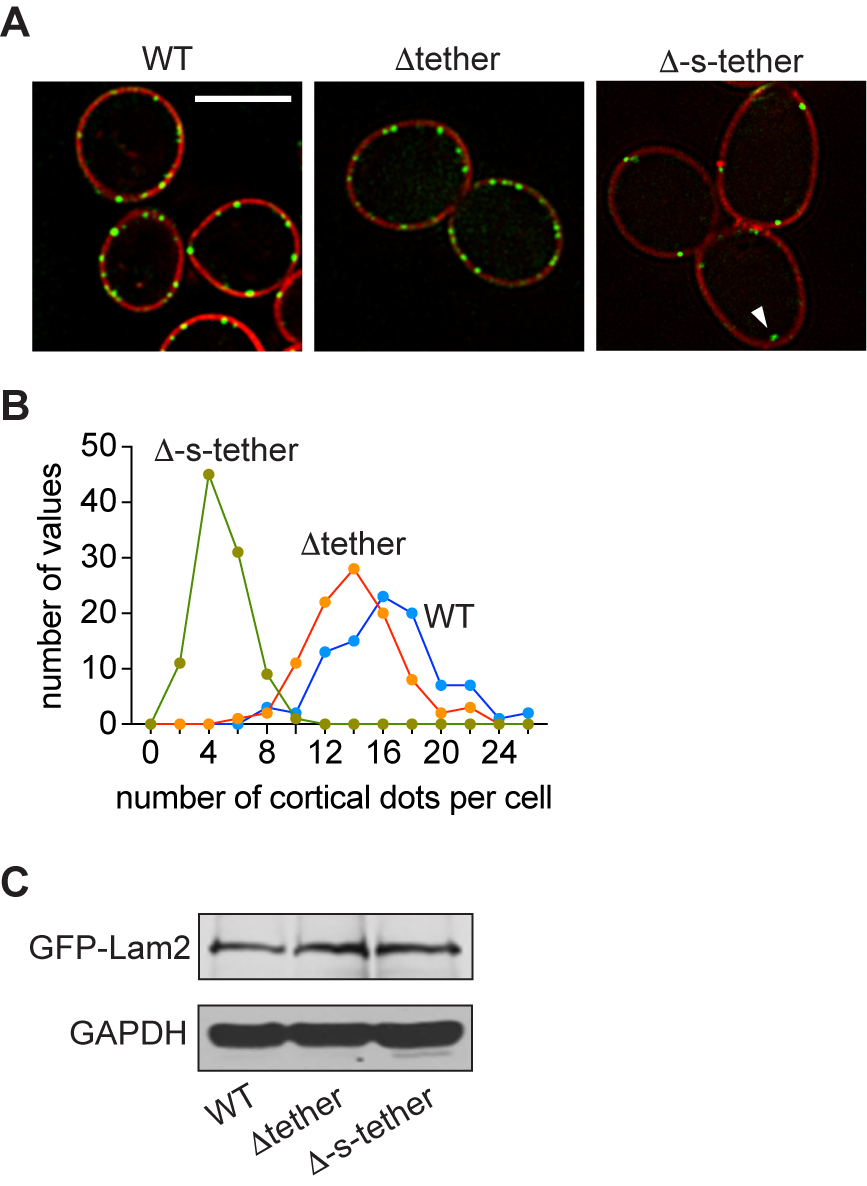

Supplement: S2 Fig — A. GFP-Lam2 was expressed from a plasmid (pGFP-Lam2) in WT (SEY6210), Δtether (ANDY198), and Δ-s-tether (CBY5838) cells. The cells were stained with CellMask Orange for 5 min to mark the PM before fluorescence microscopy. Arrowhead: fluorescence in the cell interior. Scale bar = 5 μm. B. Frequency distribution indicating the number of fluorescent cortical dots in WT, Δtether, and Δ-s-tether cells expressing GFP-Lam2. More than 90 cells of each strain were scored and the distribution was plotted using a bin size of 2. The average numbers of cortical dots per cell were 16, 14, and 4 for WT, Δtether, and Δ-s-tether, respectively. C. Anti-GFP immunoblots indicated that GFP-Lam2 levels were unaffected in Δtether and Δ-s-tether cells as compared to wild type, using anti-GAPDH as the loading control. Δ-s-tether, Δ-super-tether; GAPDH, glyceraldehyde 3-phosphate dehydrogenase; GFP, green fluorescent protein; PM, plasma membrane; WT, wild type. (TIF) [file pbio.2003864.s005.tif]

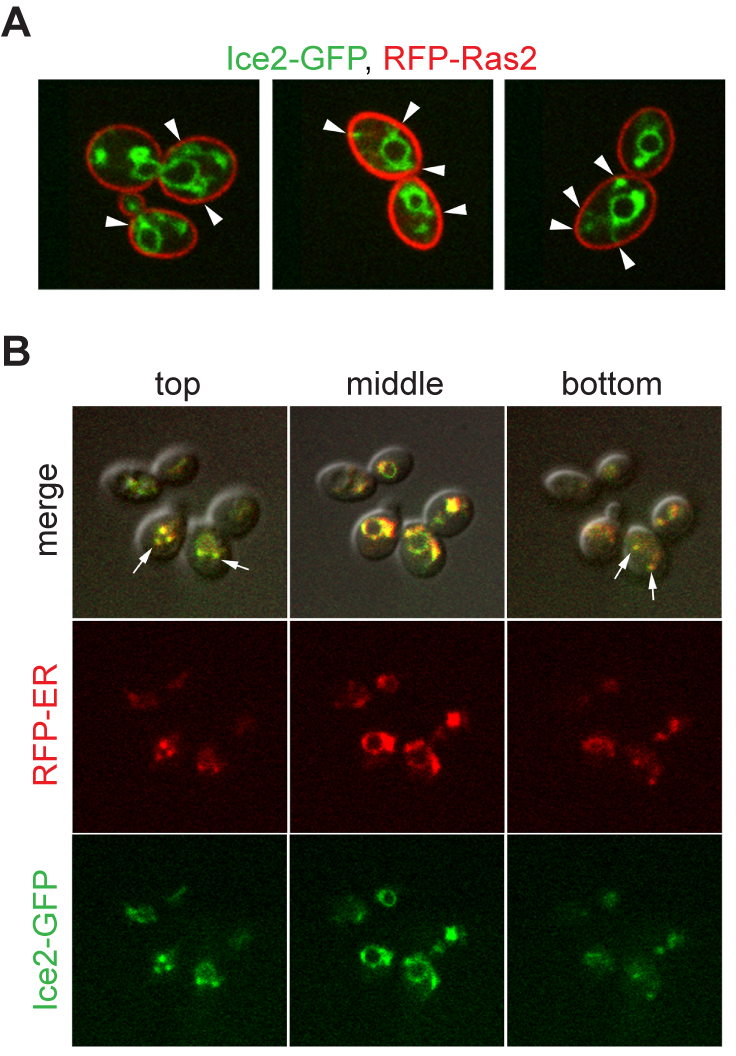

Supplement: S3 Fig — A. Ice2-GFP in Δtether cells (CBY6220) was observed at nuclear ER and in ER tubules that extend to the cell periphery, as demarcated by RFP-Ras2 (pCB1204). In this strain, the remaining cortical attachments of ER to the PM contain Ice2-GFP (arrowheads). B. Serial optical sections focused at the top, middle, and bottom of Δtether cells expressing Ice2-GFP and the ER marker RFP-ER (pCB1024). The merged images of Ice2-GFP and RFP-ER fluorescence superimposed onto corresponding DIC whole cell images indicate complete colocalization. At optical sections near the cell cortex, Ice2-GFP was present as discrete spots (arrows) in equal or greater fluorescence relative to RFP-ER, consistent with ER-PM MCSs. DIC, differential interference contrast; ER, endoplasmic reticulum; GFP, green fluorescent protein; MCS, membrane contact site; RFP, red fluorescent protein. (TIF) [file pbio.2003864.s006.tif]

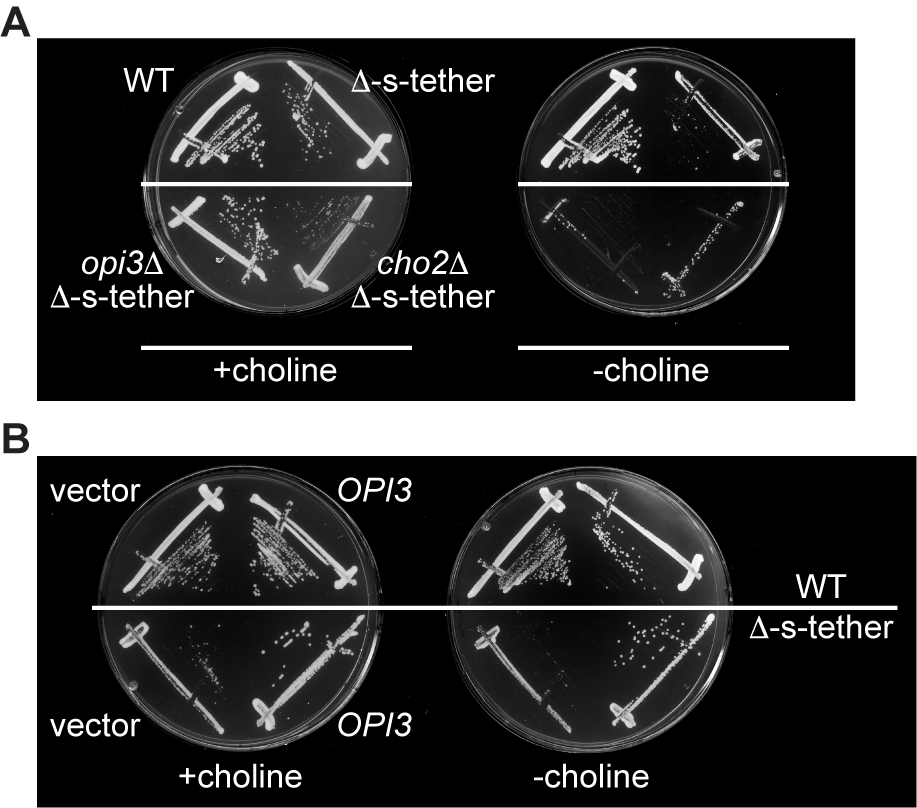

Supplement: S4 Fig — A. Deletion of CHO2 or OPI3 in Δ-s-tether cells results in synthetic growth defects when the cells are cultured without addition of choline to the growth medium. WT (SEY6210), Δ-s-tether (CBY5838), cho2Δ Δ-s-tether (CBY6267), and opi3Δ Δ-s-tether cells (CBY6271) were streaked onto selective solid media, with or without 1 mM choline, and incubated for 2 d at 30 °C. B. Increased expression of Opi3 suppresses choline-sensitive Δ-s-tether growth defects. WT and Δ-s-tether cells transformed with either the vector control (pRS416) or a plasmid expressing OPI3 from a constitutively active promoter (pOPI3) were streaked onto solid growth media supplemented with or without 1 mM choline, as indicated, and incubated for 2 d at 25 °C. Δ-s-tether, Δ-super-tether; WT, wild type. (TIF) [file pbio.2003864.s007.tif]

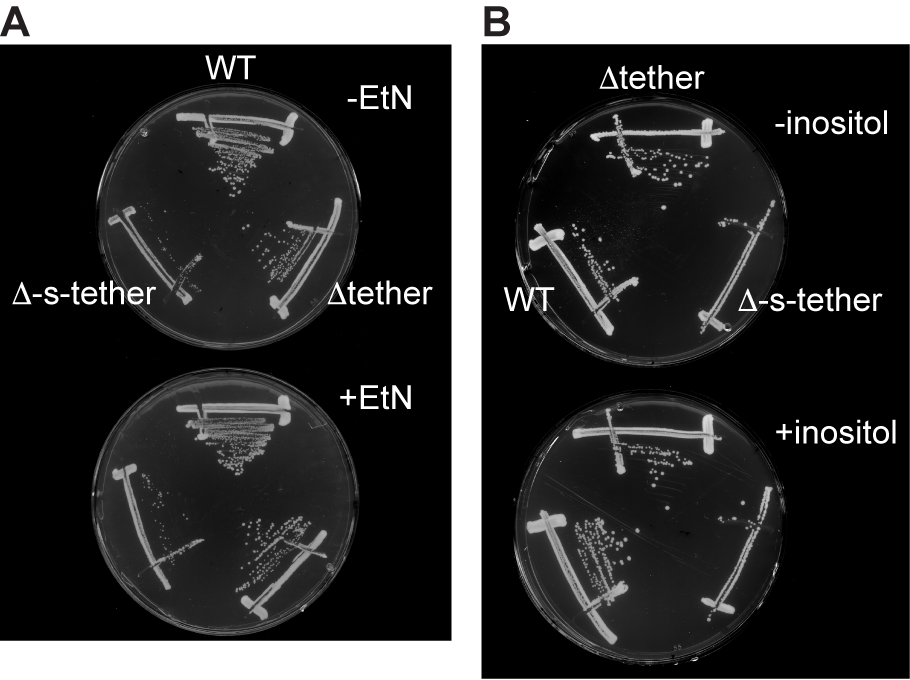

Supplement: S5 Fig — WT (SEY6210), Δtether (ANDY198), and Δ-s-tether (CBY5838) cells were streaked onto solid growth media supplemented with 1 mM ethanolamine (A) or 75 μM inositol (B), as indicated, and incubated at 30 °C for 2 or 3 d, respectively. Δ-s-tether, Δ-super-tether; WT, wild type. (TIF) [file pbio.2003864.s008.tif]

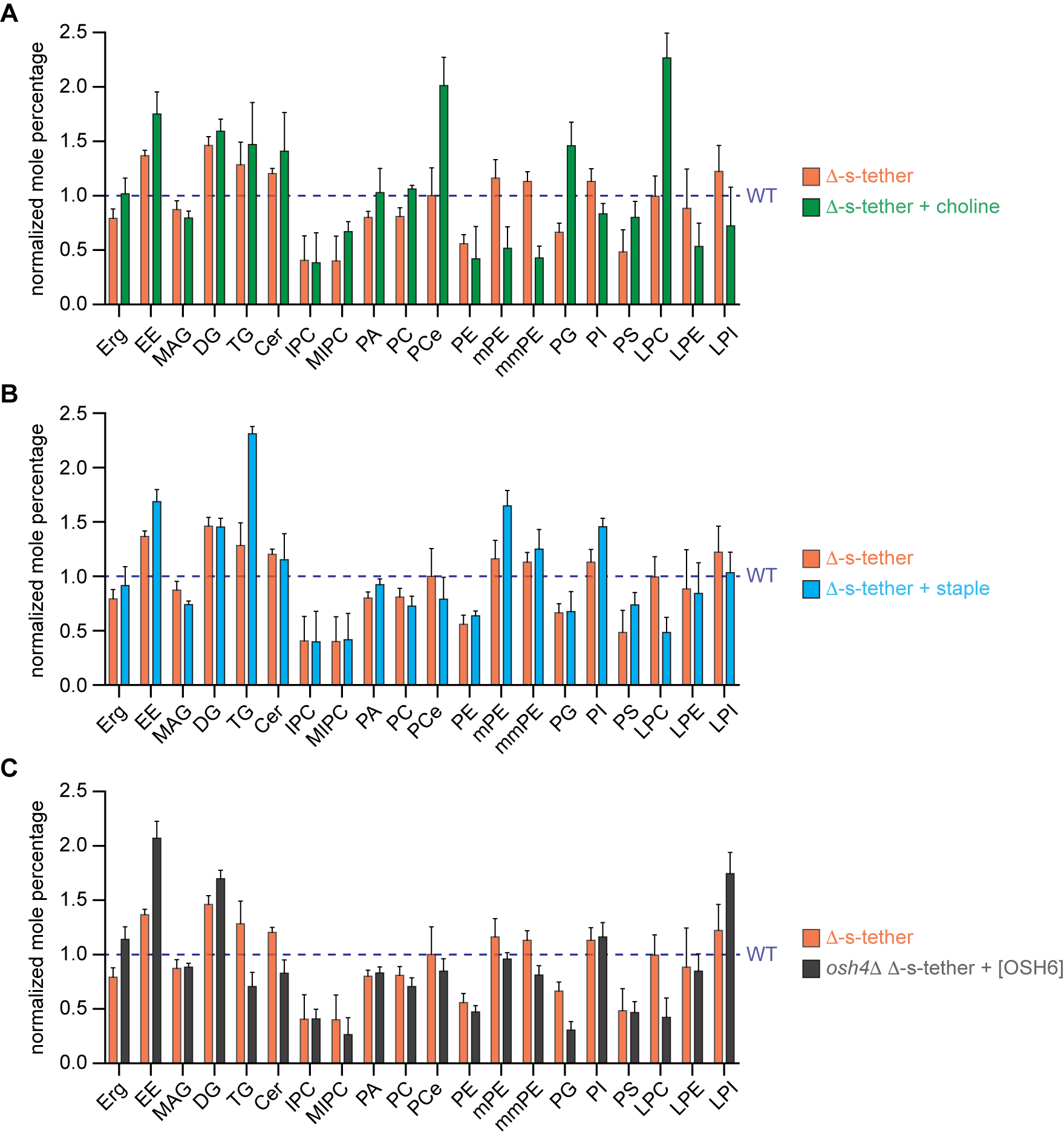

Supplement: S6 Fig — A. Comparison of the lipid composition of Δ-s-tether cells grown in the absence or presence of 1 mM choline. B. Comparison of the lipid composition of Δ-s-tether cells and Δ-s-tether cells expressing an artificial tether (“staple”). The cells were grown in synthetic medium without choline. C. Comparison of the lipid composition of Δ-s-tether cells and osh4Δ Δ-s-tether expressing Osh6. In all panels, lipid compositions are presented as a normalized mole percentage relative to WT (blue dotted line set to 1.0). The data represent the mean ± SEM derived from the analysis of five independent samples. Δ-s-tether, Δ-super-tether; Cer, ceramide; DG, diacylglycerol; EE, ergosteryl ester; Erg, ergosterol; IPC, inositol-phosphoceramide; LPC, lyso PC; LPE, lyso PE; LPI, lyso PI; MAG, monoacylglycerol; MIPC, mannosylinositol phosphoceramide; mmPE, dimethyl PE; mPE, monomethyl PE; Osh, OSBP homologue; PA, phosphatidic acid; PC, phosphatidylcholine; PCe, ether phosphatidylcholine; PE, phosphatidylethanolamine; PG, phosphatidylglycerol; PI, phosphatidylinositol; PS, phosphatidylserine; TG, triacylglycerol; WT, wild type. (TIF) [file pbio.2003864.s009.tif]

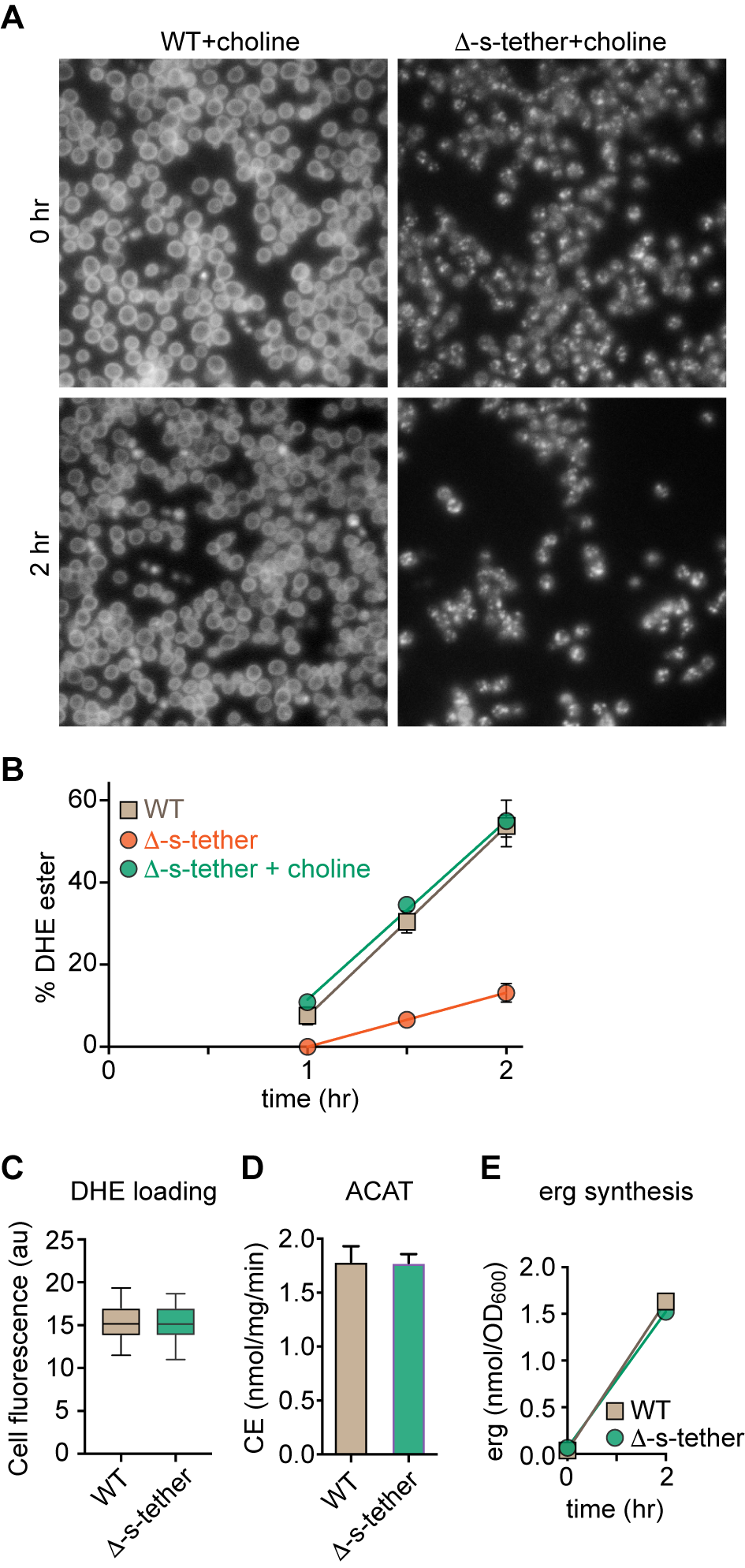

Supplement: S7 Fig — A. Representative images of choline-grown WT and Δ-s-tether cells obtained immediately after DHE loading (chase time = 0 h) and 2 h after incubation under aerobic conditions. The punctae seen in the 2 h chase images correspond to LDs. B. DHE esters were quantified at different times during the aerobic chase period by analyzing hexane/isopropanol extracts of the cells by HPLC equipped with an in-line UV detector. The data are represented as percentage of DHE ester recovered (= DHE ester/(DHE + DHE ester)). C. Incorporation of DHE into the PM of choline-grown cells, quantified using fluorescence images acquired immediately after the hypoxic incubation period, as detailed in Fig 3E. Fifty cells were analyzed. The box and whiskers plot shows the mean of the measurements, with whiskers ranging from the minimum to the maximum value measured. D. Microsomes from choline-grown WT and Δ-s-tether cells were assayed for their ability to esterify [3H]cholesterol on the addition of oleoyl-CoA, as described in Fig 3F. The bar chart shows the mean ± SEM (n = 3) of ACAT activity as the rate of production of CE per mg microsomal protein per minute. E. The amount of ergosterol in choline-grown WT and Δ-s-tether cells (nmol per OD600 of cell suspension) was measured by lipid extraction and HPLC at the start and end of the aerobic chase period. Each data point represents a triplicate measurement (the error bars are contained within the symbol used for plotting). Δ-s-tether, Δ-super-tether; ACAT, acetyl-CoA acetyltransferase; CoA, coenzyme A; CE, cholesteryl ester; DHE, dehydroergosterol; HPLC, high-performance liquid chromatography; LD, lipid droplet; OD, optical density; PM, plasma membrane; UV, ultraviolet; WT, wild type. (TIF) [file pbio.2003864.s010.tif]

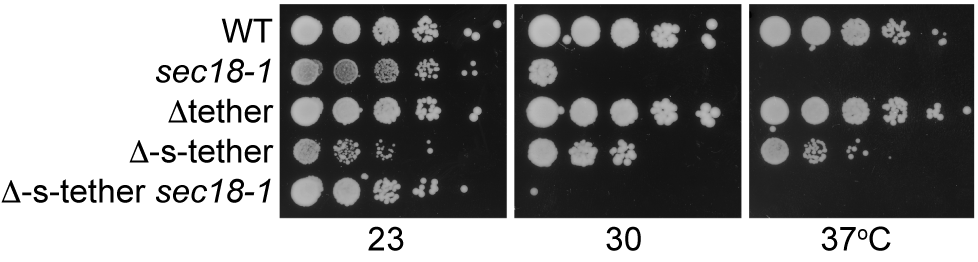

Supplement: S8 Fig — Tenfold serial dilutions of WT (SEY6210), sec18-1ts (CBY2859), Δtether (ANDY198), Δ-s-tether (CBY5988), and Δ-s-tether sec18-1ts (CBY5851) cultures were spotted on synthetic complete medium and incubated at the indicated temperatures for 3 d. Δ-s-tether, Δ-super-tether; WT, wild type. (TIF) [file pbio.2003864.s011.tif]

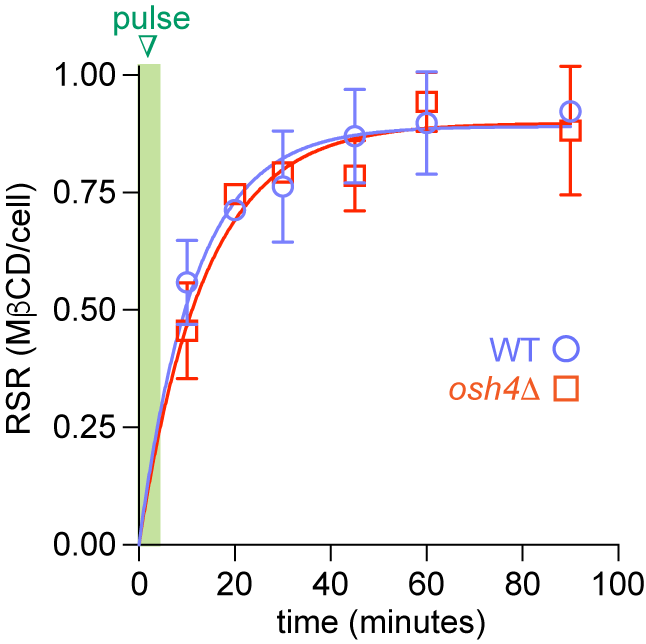

Supplement: S9 Fig — Transport of newly synthesized ergosterol to the PM in WT (SEY6210) and osh4Δ (HAB821) cells were measured by pulse-chase radiolabeling as described in Fig 3, using MβCD extraction rather than PM isolation to quantify transport. A pulse of [3H]ergosterol was generated in the ER by labeling cells for 4 min with [3H]methyl-methionine. Samples were chased for the indicated times. At each chase point, an aliquot of cells was removed, dosed with energy poisons, placed on ice, and incubated with MβCD. Following incubation, the sample was centrifuged and the MβCD-containing supernatant was removed from the cell pellet. Ergosterol was recovered from MβCD-ergosterol complexes as well as from the cell pellet by extraction with hexane/isopropanol, and its specific radioactivity was determined by HPLC (UV detection). The ratio of the specific radioactivity of ergosterol in MβCD-ergosterol complexes versus the cell homogenate RSR provides a measure of transport. Data points represent the mean ± SEM of three independent experiments, each of which comprised duplicate measurements at the indicated time points. ER, endoplasmic reticulum; HPLC, high-performance liquid chromatography; MβCD, methyl-β-cyclodextrin; PM, plasma membrane; RSR, relative specific radioactivity; UV, ultraviolet; WT, wild type. (TIF) [file pbio.2003864.s012.tif]

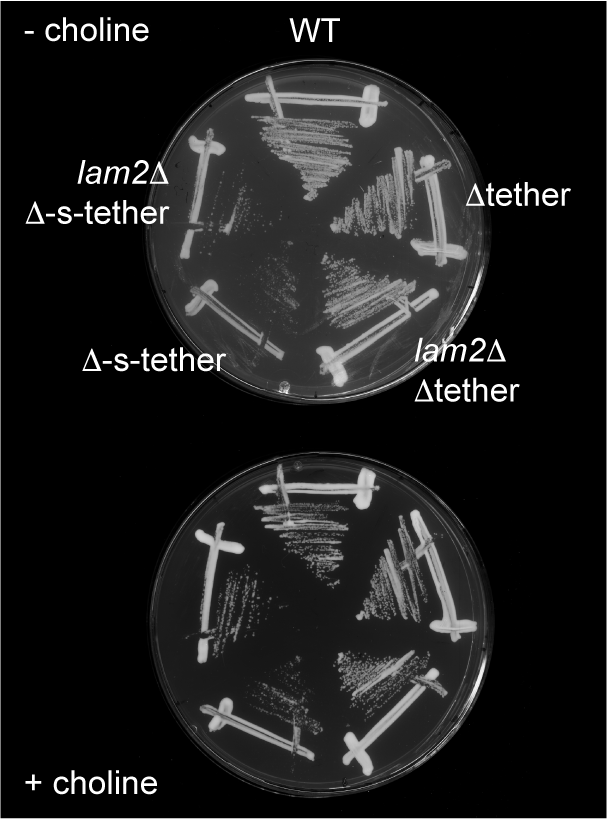

Supplement: S10 Fig — WT (SEY6210), Δtether (ANDY198), Δ-s-tether (CBY5838), lam2Δ Δtether (CBY6150), and lam2Δ Δ-s-tether cells (CBY6150) were streaked onto selective solid media with and without 1 mM choline and incubated for 2 d at 30 °C. Δ-s-tether, Δ-super-tether; WT, wild type. (TIF) [file pbio.2003864.s013.tif]

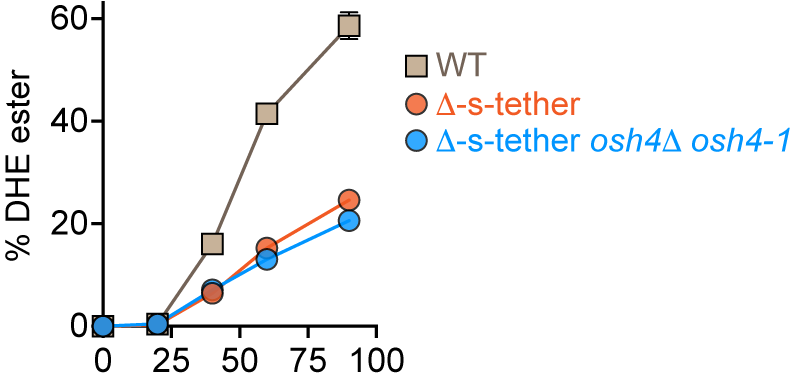

Supplement: S11 Fig — WT (SEY6210), Δ-s-tether (CBY5838), and osh4-1 osh4Δ Δ-s-tether (CBY6031) were inoculated from a saturated overnight culture into fresh media (complete synthetic media for WT and Δ-s-tether and the same medium without leucine for osh4-1 osh4Δ Δ-s-tether) supplemented with 20 μg/mL DHE and 0.5% Tween:ethanol. The cells were incubated under hypoxic conditions for 36 h at 30 °C before being transferred to 37 °C for 1 h (continuing in hypoxic conditions) and then chased aerobically for the indicated time points at 37 °C. DHE esterification was measured as described in Fig 3. Δ-s-tether, Δ-super-tether; DHE, dehydroergosterol; WT, wild type. (TIF) [file pbio.2003864.s014.tif]

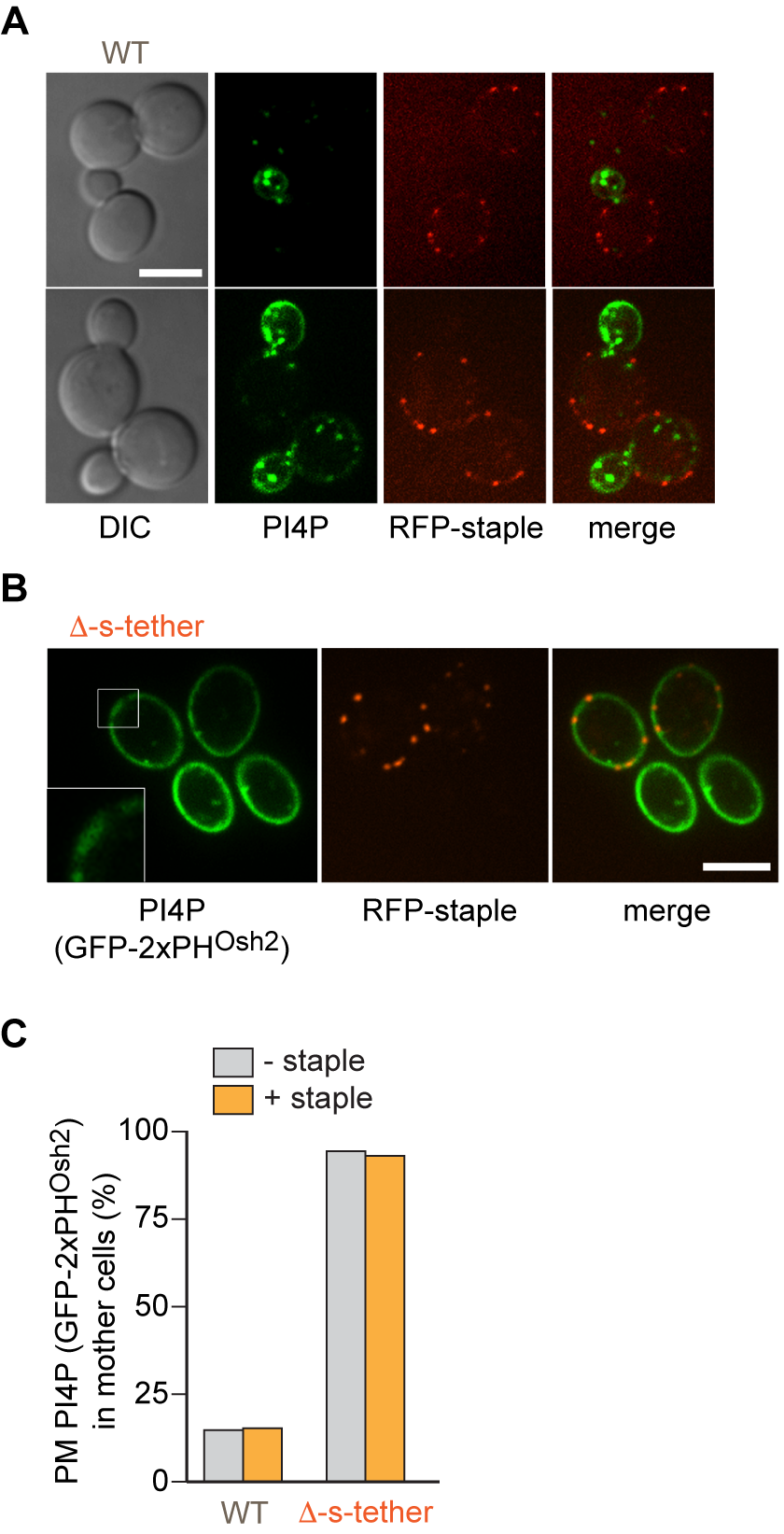

Supplement: S12 Fig — A. Wild-type (SEY6210) cells expressing an mCherry-tagged version of the artificial RFP-staple (RFP-staple; pCB1188) and cotransformed with the PI4P sensor GFP-2xPHOSH2 (pTL511), as shown by DIC and fluorescence confocal microscopy. Scale bar = 5 μm. B. Fluorescent images of Δ-s-tether cells (CBY5838) co-expressing GFP-2xPHOSH2 and the artificial RFP-staple. The boxed region represents an enlarged region shown in the inset, where gaps in the uniform PM PI4P fluorescence coincide with the presence of the artificial RFP-staple. Scale bar = 5 μm. C. Quantification of mother cell GFP-2xPHOSH2 fluorescence at the PM observed as a percentage of all wild-type and Δ-s-tether cells (n > 100 cells). Δ-s-tether, Δ-super-tether; DIC, differential interference contrast; ER, endoplasmic reticulum; PI4P, phosphatidylinositol-4-phosphate; PM, plasma membrane; RFP, red fluorescent protein. (TIF) [file pbio.2003864.s015.tif]

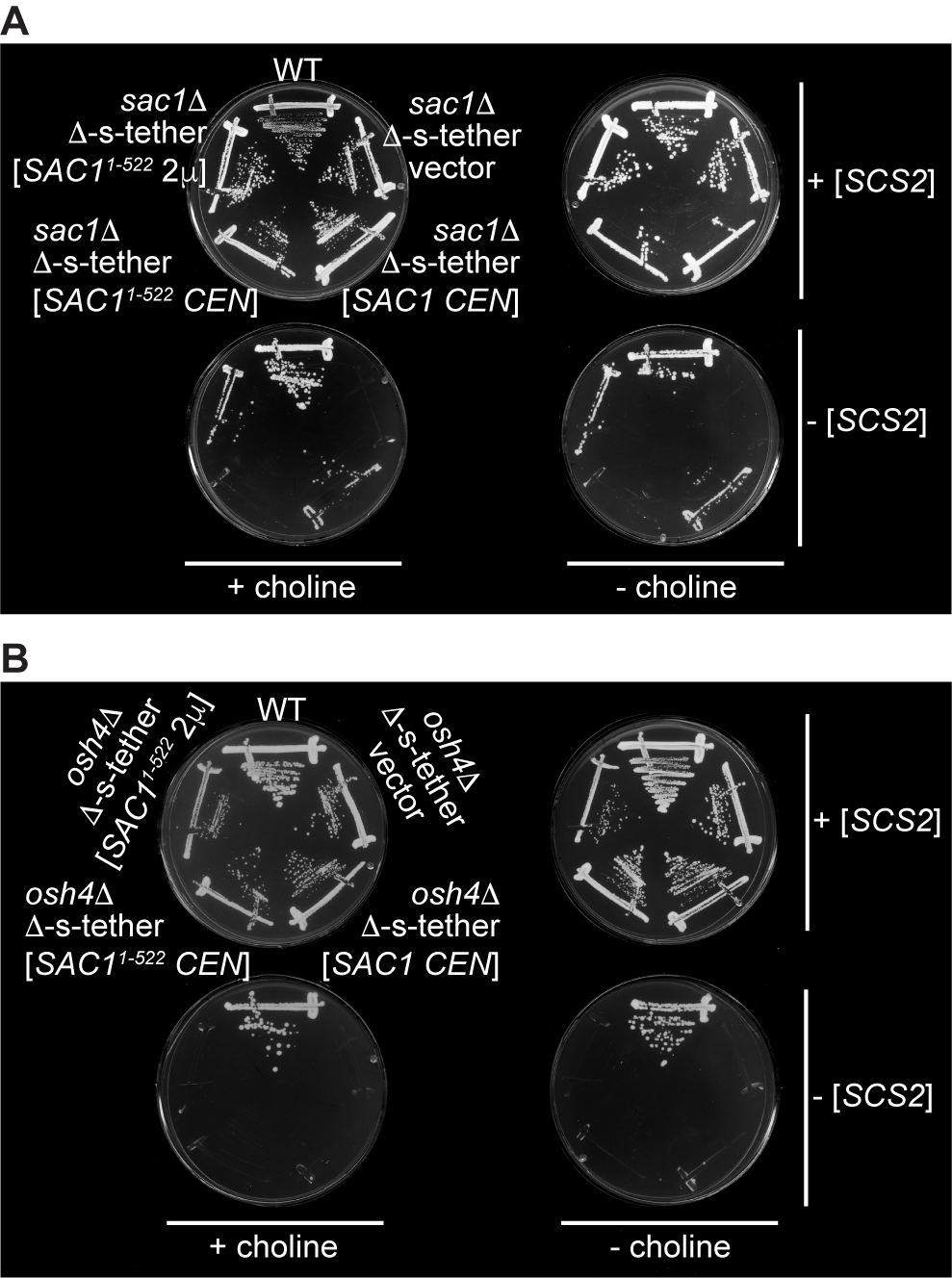

Supplement: S13 Fig — (A) WT (SEY6210) and sac1Δ Δ-s-tether (CBY6146) cells expressing an episomal copy of SCS2 (pSCS2) were transformed with the vector control (YCplac111) or plasmids expressing SAC1 (pRS415 SAC1), SAC11–522 (pRS415 SAC11–522), or SAC11–522 expressed from a high-copy plasmid (pRS425 SAC11–522). Cells were streaked onto solid growth media containing 5′-FOA (to select against strains that cannot growth without SCS2), supplemented with and without 1 mM choline, for 3 d at 30 °C; SAC1 and high-copy SAC11–522 suppressed sac1Δ Δ-s-tether synthetic lethality, regardless of choline addition. (B) WT and osh4Δ Δ-s-tether (CBY5988) cells containing an episomal copy of SCS2 were transformed with the vector control or plasmids expressing SAC1, SAC11–522, or high-copy SAC11–522. Cells were streaked onto solid 5′-FOA–containing media with and without 1 mM choline and incubated for 3 d at 30 °C. In the absence of SCS2, neither SAC1 nor SAC11–522 expression suppressed osh4Δ Δ-s-tether synthetic lethality. Δ-s-tether, Δ-super-tether; WT, wild type. (TIF) [file pbio.2003864.s016.tif]

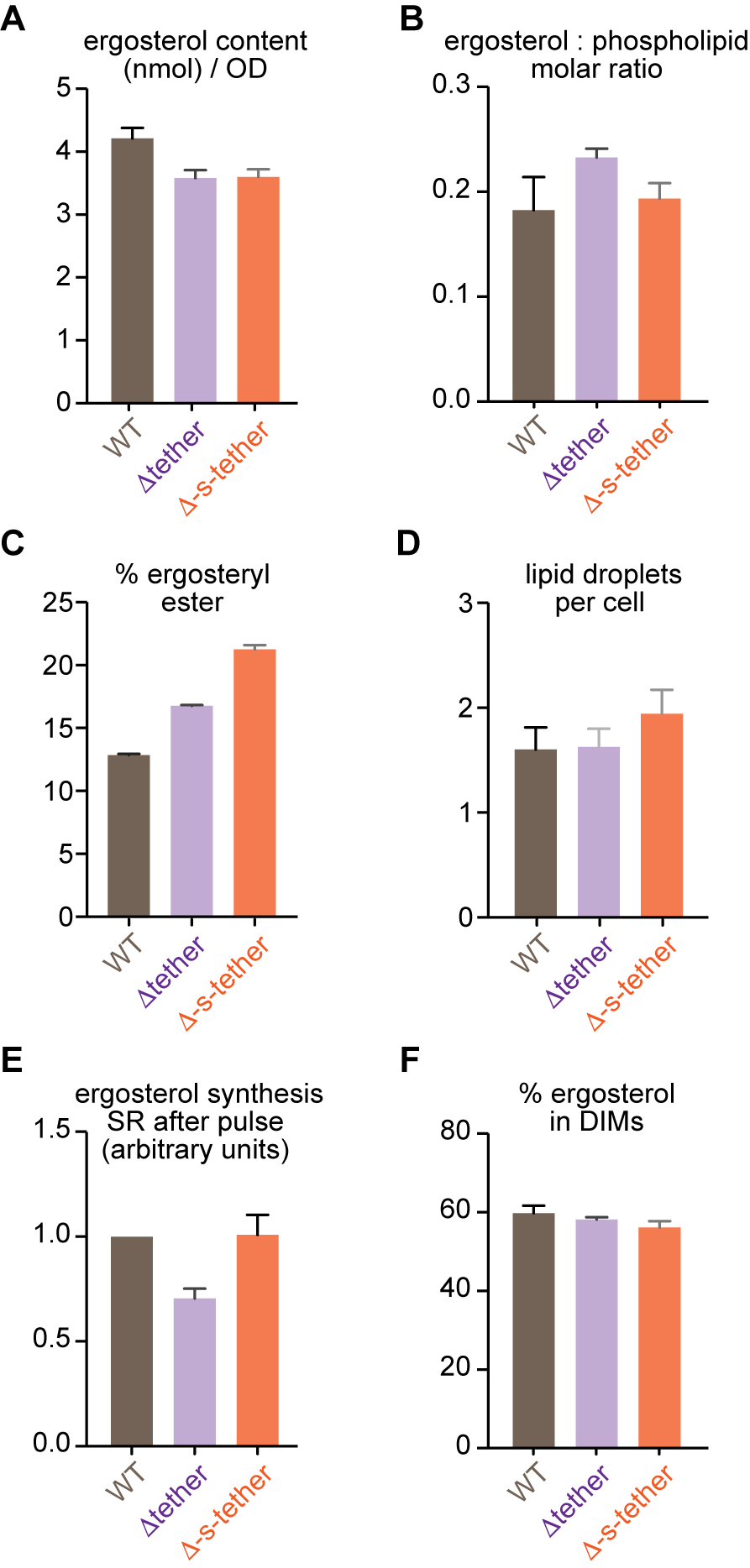

Supplement: S14 Fig — A–C. Quantification of ergosterol, ergosterol:phospholipid molar ratio, and ergosteryl ester in WT (SEY6210), Δtether (ANDY198), and Δ-s-tether (CBY5838) cells. Lipids were extracted and quantified as described in Materials and methods. D. Quantification of the number of lipid droplets per cell (n > 100 cells counted for each strain). E. The SR of ergosterol after labeling cells for 4 min with [3H]methyl-methionine was determined by HPLC, as described in Fig 3, and normalized to that of WT cells (set arbitrarily to 1.0). F. DIMs were prepared by incubating cells with ice-cold Triton X-100. The proportion of ergosterol in DIMs versus whole cells was quantified by solvent extraction, followed by HPLC analysis. Δ-s-tether, Δ-super-tether; DIM, detergent-insoluble membrane; HPLC, high-performance liquid chromatography; SR, specific radioactivity; WT, wild type. (TIF) [file pbio.2003864.s017.tif]

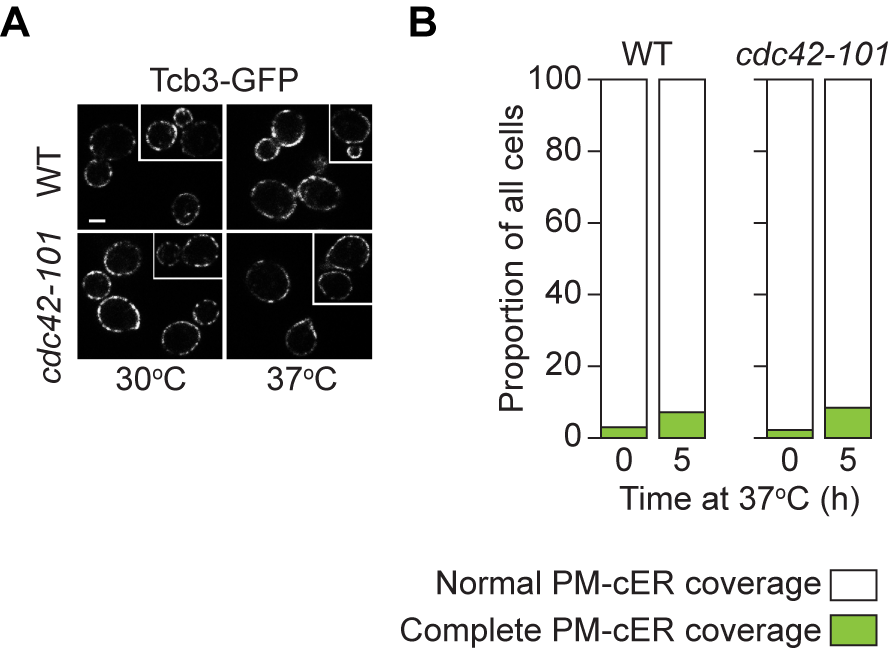

Supplement: S15 Fig — A. Discontinuous cortical Tcb3-GFP distribution was observed in WT (CBY5942) and cdc42-101ts (CBY5944) cells incubated 1 h at 37 °C, or at 30 °C. Scale bar = 2 μm. B. Percentage of cells with normal discontinuous Tcb3-GFP distribution versus continuous cortical localization along the PM in WT and cdc42-101ts cells. Even after G1-arrest for 5 h at 37 °C, Tcb3-GFP in cdc42-101ts cells was indistinguishable from WT. ER, endoplasmic reticulum; GFP, green fluorescent protein; MCS, membrane contact site; PM, plasma membrane; Tcb, tricalbin; WT, wild type. (TIF) [file pbio.2003864.s018.tif]

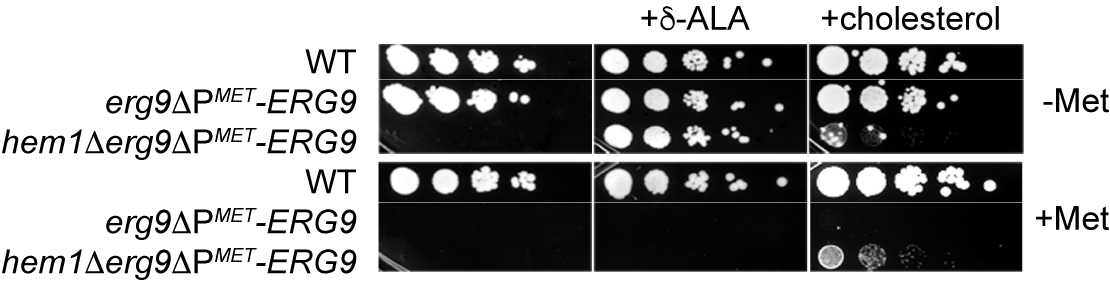

Supplement: S16 Fig — Tenfold serial dilutions of WT (with an integrated PMET-ERG9 construct; CBY918), erg9Δ PMET-ERG9 (CBY745), and hem1Δ erg9Δ PMET-ERG9 (CBY5844) cultures on synthetic solid medium with (+Met) or without (−Met) methionine, containing (as shown) cholesterol, +δ-ALA, or neither. In the absence of δ-ALA supplementation, all hem1Δ strains require methionine for growth, and cholesterol uptake cannot occur under aerobic conditions without the hem1Δ mutation. In the presence of methionine, which represses PMET-ERG9 expression and sterol synthesis, hem1Δ erg9Δ PMET-ERG9 cells grow (albeit slowly) with 25 μg/mL cholesterol supplementation. δ-ALA, δ-aminolevulinic acid; WT, wild type. (TIF) [file pbio.2003864.s019.tif]

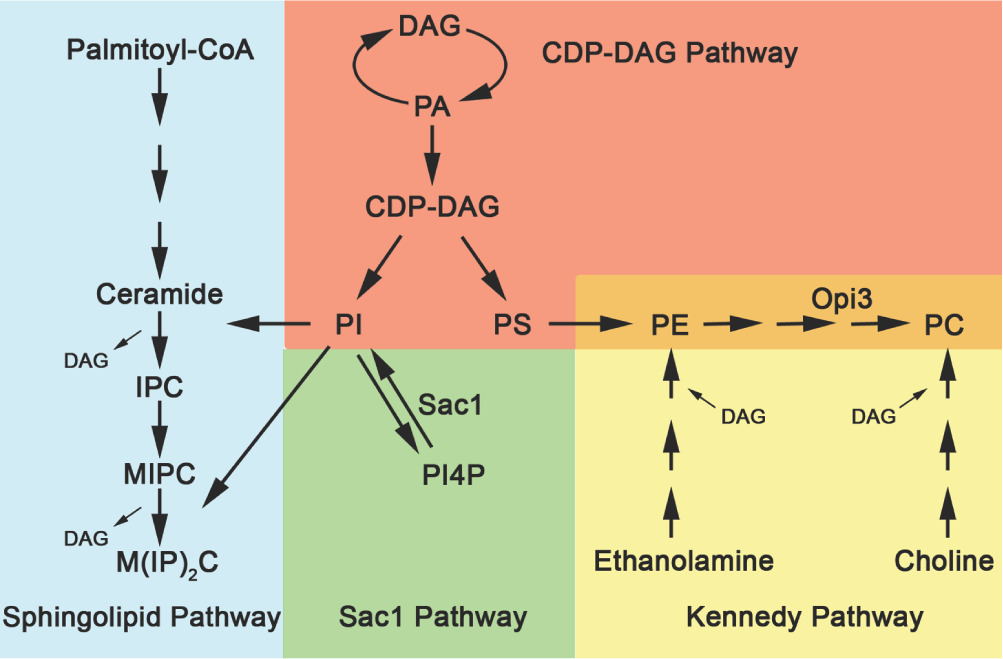

Supplement: S17 Fig — See text for details. (TIF) [file pbio.2003864.s020.tif]
